# A Comparative Study of Common Nature-Inspired Algorithms for Continuous Function Optimization

**DOI:** 10.3390/e23070874

**Published:** 2021-07-08

**Authors:** Zhenwu Wang, Chao Qin, Benting Wan, William Wei Song

**Affiliations:** 1 Department of Computer Science and Technology, China University of Mining and Technology, Beijing 100083, China; sqt1900405077@student.cumtb.edu.cn; 2 School of Software and IoT Engineering, Jiangxi University of Finance & Economics, Nanchang 330013, China; wanbenting@jxufe.edu.cn; 3 Department of Information Systems, Dalarna University, S-791 88 Falun, Sweden

**Keywords:** nature-inspired algorithm, meta-heuristic algorithm, swarm intelligence algorithm, bio-inspired algorithm, black-box optimization benchmarking, statistical test

## Abstract

Over previous decades, many nature-inspired optimization algorithms (NIOAs) have been proposed and applied due to their importance and significance. Some survey studies have also been made to investigate NIOAs and their variants and applications. However, these comparative studies mainly focus on one single NIOA, and there lacks a comprehensive comparative and contrastive study of the existing NIOAs. To fill this gap, we spent a great effort to conduct this comprehensive survey. In this survey, more than 120 meta-heuristic algorithms have been collected and, among them, the most popular and common 11 NIOAs are selected. Their accuracy, stability, efficiency and parameter sensitivity are evaluated based on the 30 black-box optimization benchmarking (BBOB) functions. Furthermore, we apply the Friedman test and Nemenyi test to analyze the performance of the compared NIOAs. In this survey, we provide a unified formal description of the 11 NIOAs in order to compare their similarities and differences in depth and a systematic summarization of the challenging problems and research directions for the whole NIOAs field. This comparative study attempts to provide a broader perspective and meaningful enlightenment to understand NIOAs.

## 1. Introduction

Nature-inspired optimization algorithms (NIOAs), defined as a group of algorithms that are inspired by natural phenomena, including swarm intelligence, biological systems, physical and chemical systems and, etc. [1]. NIOAs include bio-inspired algorithms and physics- and chemistry-based algorithms; the bio-inspired algorithms further include swarm intelligence-based and evolutionary algorithms [1]. NIOAs are an important branch of artificial intelligence (AI), and NIOAs have made significant progress in the last 30 years. Thus far, a large number of common NIOAs and their variants have been proposed, such as genetic algorithm (GA) [2], particle swarm optimization (PSO) algorithm [3], differential evolution (DE) algorithm [4], artificial bee colony (ABC) algorithm [5], ant colony optimization (ACO) algorithm [6], cuckoo search (CS) algorithm [7], bat algorithm (BA) [8], firefly algorithm (FA) [9], immune algorithm (IA) [10], grey wolf optimization (GWO) [11], gravitational search algorithm (GSA) [12] and harmony search (HS) algorithm [13]. In addition to the theoretical studies of NIOAs, many previous works have made an in-depth investigation on how the NIOAs are applied to various domains. Single NIOAs have been reviewed comprehensively [14,15,16,17,18,19,20,21,22,23,24,25], which present the algorithms and their variants at a good breadth and depth. In the rest of this chapter, we summarize the current survey work of the NIOAs, discuss our motivations for this survey, present our research methodologies and scope of this work and finally, describe our contributions to this field.

### 1.1. Summary of the Current Survey Work

From our observation, although reviews of specific NIOAs [14,15,16,17,18,19,20,21,22,23,24,25] are very common, there have not been many attempts to compare various NIOAs in terms of the general criteria. Only a few surveys [26,27,28,29,30] (named horizontal NIOAs reviews) adopted the narrative literature review approach to discuss a series of NIOAs, including their basic principles, variants and application domains. Specifically, Chakraborty [26] discussed eight bio-inspired optimization algorithms (BIOAs) that can be divided into insect-based algorithms, inspired by ants, bees, fireflies and glow-worms and animal-based algorithms, inspired by bats, monkeys, lions and wolves; Kar [28] detailed the principles, developments and applications of 12 BIOAs, including the neural networks, GA, PSO, ACO, ABC, bacterial foraging (BFO) algorithm, CS, FA, shuffled frog leaping algorithm (SFLA), BA, flower pollination (FP) algorithm and artificial plant optimization algorithm (APOA); Parpinelli [30] summarized the principles, application fields and meta-heuristics information of nine BIOAs, such as bees algorithm, ABC, marriage in honey-bees optimization (MBO) algorithm, BFO, glow-worm swarm optimization algorithm (GSOA), FA, slime mold optimization algorithm (SMOA), roach infestation optimization (RIO) algorithm and BA. In addition to the above reviews, some literature compared the performance of NIOAs via a series of benchmark functions. Through a number of statistical tests, Ab Wahab [27] compared seven BIOAs, including GA, ACO, PSO, DE, ABC, GSOA and CS; Chu [29] only analyzed three BIOAs, including PSO, ACO and ABC, on three benchmark functions.

In all, all the above survey works provide good references for NIOAs, but these reviews are not comprehensive and in-depth. For example, the survey work [26,28,30] merely introduces the principles, variants and applications for different BIOAs, without involving their performance comparison, which provides an important basis to the improvement and application for BIOAs. Some reviews [27,29] discuss the performance comparison of BIOAs. However, the comparison in [27] for the seven BIOAs is inadequate because the chosen BIOAs are incomplete, most benchmark functions are low-dimensional and the experimental results are only represented by mean error (comparison of convergence speed is not considered). The review in [29] only compares three BIOAs on three benchmark functions; the comparison algorithms and experimental work are quite narrow. Besides the above shortcomings, these problems also exist: selected NIOAs are not popular, and the criterion of selection is not clear. Furthermore, common challenging problems of NIOAs have not been extracted and discussed in all the above survey work [26,27,28,29,30]. These are, for instance, the common characteristics and differences for the NIOAs, the challenges and future directions for the NIOAs field and the systematic summary of improvement methods for all the chosen NIOAs, just to mention a few.

### 1.2. Motivations

As discussed in Section 1.1, the current horizontal NIOAs reviews still have some important issues that have not been discussed at a sufficient depth; we think it worthwhile to make a comprehensive comparison and analysis study of the common NIOAs for the following four reasons.

1. Thus far, questions such as how many NIOAs have been proposed and which NIOAs are the research hotspots that should be addressed and discussed. It is necessary to distinguish the hotspots of NIOAs, but it is very difficult to collect all the NIOAs in an all-around way. With our best effort, we search for NIOAs that we can reach and identify the hotspot ones from our observation. To our knowledge, no similar work has been completed, and for existing horizontal NIOAs reviews, either the selection criteria for NIOAs [27,28,29,30] are not clear or the selected algorithms are not common [31,32,33].

2. Different proposals of the NIOAs for different purposes have created great confusion as to which method fits what situation, and it is strongly required to understand what the common characteristics are of these hotspots algorithms and what the differences are. To study and compare the characteristics of the hotspot NIOAs can provide not only a broader perspective to the improvement of the current NIOAs but also a solid and feasible cornerstone for building up the new problem-oriented NIOAs.

3. Hybridization is an important method to improve the performance of NIOAs. When considering a hybridization of different NIOAs, many proposers more often than not claim that the selected NIOAs have shortcomings to be improved, for example, being easy to fall into local optimum and having a slow convergence speed, on the one hand, and consider that some algorithms have advantages that could be utilized as necessary complements, such as rapid convergence speed and good ability of global exploration, on the other hand. Thus, in order to validate and compare the performance of these common NIOAs, a construction of comprehensive experiments of common NIOAs is indispensable.

4. To our knowledge, most survey work focuses on introducing NIOAs’ principles, variants and application domains and little work has so far been completed to summarize the general improvement methods for all the chosen NIOAs and to analyze their challenges and future directions for the whole field of NIOAs, and all these issues are very important and critical to the development of NIOAs.

It should be noted that some so-called “novel” NIOAs that have been proposed from time to time are actually ”the same old stuff with a new label” and deteriorate the research atmosphere of NIOAs. However, this issue is not the scope of this paper. It is undeniable that there are many excellent works in the field of NIOAs, which have greatly promoted the development of NIOAs. The main purpose of this work is to objectively analyze the existing commonly used NIOAs and discuss their characteristics, performance comparison, challenges and future directions.

### 1.3. Research Methodology

**Figure 1:**
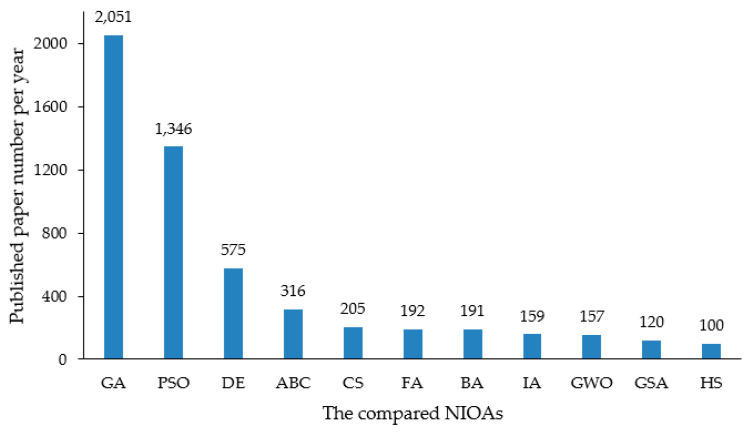
Number of papers published per year until 13 October 2020 (from *Web of Science* and *Scopus* databases).

**Figure 2:**
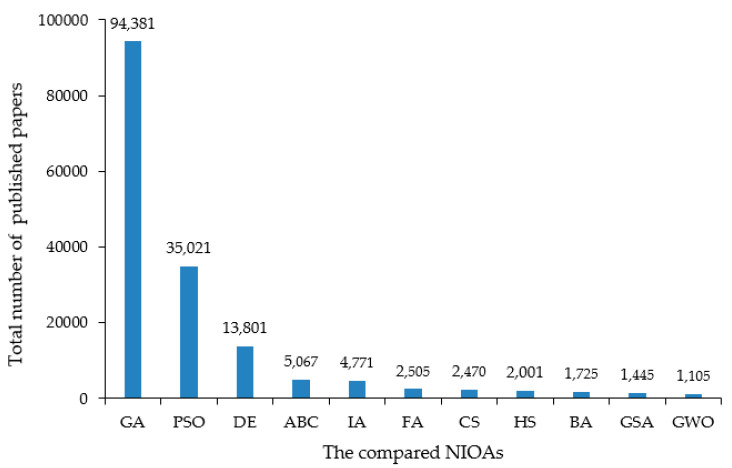
Total number of papers published until 13 October 2020 (from *Web of Science* and *Scopus* databases).

The research is conducted in multiple stages. Firstly, the meta-heuristic algorithms (MHAs) that are to be scrutinized are identified, which include NIOAs and non-nature-inspired optimization algorithms (NNIOAs). There are numerous MHAs, and they are being developed continuously. Most of the MHAs are independently developed and conducted and are labeled under the terms of swarm intelligence (SI), BIOA, NIOA and NNIOA. In this work, we search for MHAs as many as possible from the *Web of Science*, *Google Scholar dictionary* and *Scopus* database by using specific keywords, such as *swarm intelligence optimization*, *intelligent algorithm*, *heuristic*, *meta-heuristic*, *bio-inspired algorithm* and *nature-inspired algorithm*. After identifying these algorithms, we adopted the *Google engine* to confirm whether they are MHAs or not. Then, some “common” (or “hotspot”) NIOAs are selected to conduct the comparison task, and how to judge whether the algorithm is “common” has become another problem. In order to identify the “most common” NIOAs, we compute the average number of articles per year and the total number of published articles for a candidate NIOA. The total article number of a certain NIOA is computed through searching algorithm name as *TITLE* in *Web of Science* and *Scopus* databases. The advantage of this method is to ensure that classic NIOAs, such as GA, PSO, DE, are included in the comparison work, while those that, compared with the contemporary algorithms are relatively seldom used in the whole scientific community, are excluded: for example, the self-organizing migrating algorithm, spiral millipede-inspired routing algorithm and benchmarking-based optimization algorithm. According to this approach, more than 120 MHAs (see Table S1 in Supplementary Material A) are identified, and among them, 11 NIOAs are selected for scrutiny in this survey work. Through the aforementioned calculation methods, we include a NIOA which has been discussed in more than 100 published papers per year and in more than 1000 papers in total (described in Figure 1 and Figure 2, respectively). The statistical performance comparison of these NIOAs is with the BBOB functions; compared with the functions described by explicit equations, they have uncertainty and noise, which can ensure the fairness of experimental results.

### 1.4. Scope of Discussion

This survey work focuses on the single objective numerical optimization algorithms, which are the basis of the more complex optimization algorithms, such as the multi-objective optimization algorithms and the constrained optimization algorithms. We exclude the ant colony optimization (ACO) algorithm, ranked 4 in Table S1 of Supplementary Material A, from this survey study because it is designed to solve combinatorial optimization problems (COPs) [6] and is not in the scope of this work. We exclude the “Biogeography-Based Optimization” algorithm in Table S1 of Supplementary Material A, ranked thirteenth from this survey, since its total number of published articles 950, and the average number of articles published annually 73 both are lower than the values set for this selection of NIOAs. Finally, we consider that the selected 11 algorithms are reasonable. In this paper, we do not involve the variant methods and the applications of 11 NIOAs, since they have been discussed sufficiently in many reviews [14,15,16,17,18,19,20,21,22,23,24,25]. The selection method of NIOAs can identify the NIOAs of continuous hotspot research; some less studied algorithms are excluded, although they were brought up a long time ago (see Table S1 in Supplementary Material A).

### 1.5. Our Contributions

The contributions of this paper are as follows.

1. We present a comprehensive list of more than 120 MHAs, and make a preliminary statistical analysis of the basic information for the chosen NIOAs, which can provide a panoramic view for NIOAs study. It is the first attempt to systematically study the existing NIOAs, even though it is very hard work.

2. We analyze and summarize the common characteristics and differences of all the chosen NIOAs to provide a clear insight into the construction, design and application of NIOAs.

3. We compare and analyze the accuracy, the stability, the efficiency and the parameter sensitivity of the chosen NIOAs with different function features under high and low dimensional spaces, respectively, which can reflect the essential characteristics of each algorithm.

4. We discuss the challenges and future directions of the whole NIOAs field, which can provide a referencing framework for the future research of NIOAs.

### 1.6. Structure of the Paper

The rest of this paper is organized as follows. In Section 2, we extracted a unified representation as a foundation of comparison for the 11 NIOAs, and under this representation, the principles of 11 NIOAs have been discussed. In Section 3, the common characteristics and differences of 11 NIOAs have been analyzed and summarized. Section 4 focuses on the comparative study of the accuracy, the stability, the efficiency and the parameter sensitivity of these NIOAs with the 30 BBOB functions, and the Friedman test and Nemenyi test are constructed to analyze the performance of the compared NIOAs. In addition, the 11 NIOAs are applied to solve a constrained engineering optimization problem in Section 4. We discuss the challenges and future direction of the NIOAs in Section 5 and conclude this paper in Section 6.

## 2. Common NIOAs

Actually, most of the NIOAs have a similar structure, although they are defined in various forms. In this section, first, the common process will be extracted to offer a unified description for the NIOAs, and then the principles of the 11 NIOAs will be outlined and discussed under this unified structure. The unified representation makes it convenient to analyze the similarity and dissimilarity of these algorithms.

### 2.1. The Common Process for the 11 NIOAs

**Figure 3:**
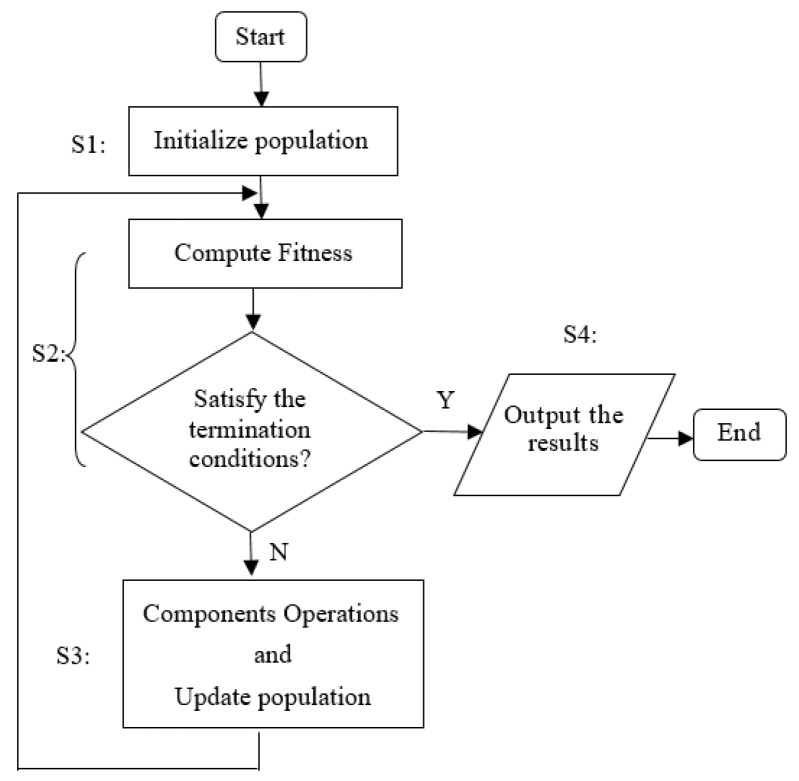
The common process of NIOAs.

The common process of most of NIOAs is described in Figure 3, which can be divided into four steps. In step S1, the population and related parameters are initialized. Usually, the initial population is generated by random methods, which ensure it covers as much solution space as possible; the population size is selected based on expert experience and specific requirements, and generally, it should be as large as possible. Most NIOAs use iterative methods, and the maximum iteration times and precision threshold are two common conditions of algorithm termination, which should also be initialized in step S1.

The fitness function is the unique indicator that reflects the performance of each individual solution, and it is designed by the target function (i.e., the BBOB functions will be described in Section 4.1), which usually has a maximum or minimum value. Generally, an individual has its own local optimal solution, and the whole population has a global optimum. In step S2, the fitness values of the population in each iteration are computed, and if the global best solution satisfies the termination conditions, NIOAs will output the results (in step S4). Otherwise, step S3 is implemented, which performs the key operations (defined by various components or operators) to exchange information among the whole population in order to evolve excellent individuals. Then, the population is updated, and the workflow jumps to step S2 to execute the next iteration. According to the above process, a set of commonly used symbols are given in Table 1 as a unified description for the 11 NIOAs, where *D* represents the dimension number of objective functions, *M* is the individual number of each NIOA and *N* the total iterative times.

### 2.2. The Principles of the 11 NIOAs

#### 2.2.1. Genetic Algorithm (GA)

Holland [2] proposed the GA algorithm, which is based on natural selection (named “selection operator $s_{o}$”), genetic (named “crossover operator $c_{o}$”) and mutation (named “mutation operator $m_{o}$”) mechanisms. The encoding method of the GA algorithm is decided by the specific problems, and common encoding schemes include binary, natural number, real number, matrix, tree and quantum. There are many types of selection, crossover and mutation operators, such as roulette wheel selection, stochastic universal sampling, local selection and tournament selection for $s_{o}$, one-point crossover, two-point crossover, multi-point crossover and uniform crossover for $c_{o}$ and the basic mutation operator (that chooses one or more genes to randomly change), the inversion operator (that randomly chooses two gene points to inverse the genes between two points), for $m_{o}$. The types of three operators are associated with the encoding schemes. Supposing $\vartheta_{1}$,$\;\vartheta_{2}$ and $\vartheta_{3}$ are the probabilities of selection, crossover and mutation, respectively, the steps of the GA algorithm are described as Algorithm 1.
**Algorithm 1** GAInput: the parameters *M*, *N*, δ, $\vartheta_{1}$,$\;\vartheta_{2}$ and $\vartheta_{3}$BeginS1: encode and initialize *M* individuals $x_{i}\left(t\right)\;$randomly, 0<i≤M, iterative times *t* = 1;S2: compute f(i), 0<i≤M, update$\;\;p_{g}\left(t\right)$, if it satisfies (*t* > *N* or precision ≤δ), then go to step S4; otherwise, go to step S3;S3: execute$\;s_{o}$,$\;c_{o}$ and $m_{o}\;$operations to generate new solutions according to $\vartheta_{1}$,$\;\vartheta_{2}$ and $\vartheta_{3}$, iterative times *t* = *t* + 1; go to step S2;S4: output the optimized results.End

#### 2.2.2. Particle Swarm Optimization (PSO) Algorithm

Kennedy [3] put forward the PSO algorithm, which simulated the bird swarm behavior. The movement method, represented by the position and velocity of the *i^th^* individual in the *d^th^* dimension for the (*t* + 1)*^th^* iteration, is described in Equation (1).

(1) $$\begin{array}{c} v_{i,d}\left(t+1\right)=v_{i,d}\left(t\right)+c_{1}*rand_{1}*\left(p_{i,d}\left(t\right)-x_{i,d}\left(t\right)\right)+c_{2}*rand_{2}*\left(p_{g,d}\left(t\right)-x_{i,d}\left(t\right)\right)\\ x_{i,d}\left(t+1\right)=x_{i,d}\left(t\right)+v_{i,d}\left(t+1\right) \end{array}\;$$

where$\;c_{1}$ and $c_{2}$ are the learning factors,$\;rand_{1}$ and $rand_{2}$ are random numbers uniformly distributed in the range [0, 1] and the velocity $v_{i}\left(t\right)$ is defined as $v_{i}\left(t\right)=\left(v_{i,1}\left(t\right),\ldots ,v_{i,d}\left(t\right),\ldots ,v_{i,D}\left(t\right)\right)$. The steps of the PSO algorithm are described as Algorithm 2.
**Algorithm 2** PSOInput: the parameters *M*, *N*, δ, $c_{1}$ and $c_{2}$BeginS1: initialize $x_{i}\left(t\right)\;$and $v_{i}\left(t\right)$ randomly, 0<i≤M, iterative times *t* = 1;S2: compute f(i) ,0<i≤M, update $p_{i}\left(t\right)$ and $p_{g}\left(t\right)$, if it satisfies (*t* > *N* or precision ≤δ), then go to step S4; otherwise, go to step S3;S3: update $x_{i}\left(t\right)\;$and $v_{i}\left(t\right)\;$according to Equation (1), iterative times *t* = *t* + 1; go to step S2;S4: output the optimized results.End

#### 2.2.3. Artificial Bee Colony (ABC) Algorithm

Karaboga [5] presented the ABC algorithm, and there are three kinds of bees: employed foragers, scouts and onlookers. An employed forager associates with one food source and shares it with other bees by certain probability; scouts are in charge of searching new food sources and onlookers find food sources through sharing information with employed foragers. The position of the *i^th^* individual on the *t^th^* iteration is $x_{i}\left(t\right)$ that is generated by Equation (2).

(2) $$x_{i,d}\left(t\right)=L_{d}+rand\left(0,1\right)*\left(U_{d}-L_{d}\right)\;$$

Here, $L_{d}$ and $U_{d}$ are the lower and upper bounds in the *d^th^* dimensional space, respectively; *rand* (0, 1) is the random number uniformly distributed in the range (0, 1). Employed foragers search for new food sources according to Equation (3).

(3) $$v_{i,d}\left(t+1\right)=x_{i,d}\left(t\right)+\varphi \left(x_{i,d}\left(t\right)-x_{j,d}\left(t\right)\right)\;$$

where 0<i, j≤M, i≠j, φ is the random number uniformly distributed in the range (0, 1). Onlookers choose food sources according to Equations (4) and (5),

(4) $$p_{i}=\frac{fit_{i}}{\sum_{i=1}^{N}fit_{i}}\;$$

(5) $$fit_{i}=\{\begin{array}{c} \frac{1}{1+f\left(i\right)},f\left(i\right)\geq 0\\ 1+abs\left(f\left(i\right)\right),otherwise \end{array}\;$$

If a food source cannot be updated after Limit times searches, the ABC algorithm deletes it and the corresponding employed forager changes to the scout; supposing there are *F* employed foragers initially, the steps of the ABC algorithm are described as Algorithm 3.
**Algorithm 3** ABCInput: the parameters *M*, *N*, δ, F, LimitBeginS1: initialize *M* individuals $x_{i}\left(t\right)$ randomly by Equation (2), and appoint $\frac{M}{2}$ bees to the employed foragers, iterative times *t* = 1;S2: compute f(i), 0<i≤M, update $p_{g}\left(t\right)$; if it satisfies (*t* > *N* or precision ≤δ), then go to step S4; otherwise, go to step S3;S3: employed foragers search new food sources by Equation (3) and compute f(i); update the food sources if the new one is better than the old one; onlookers choose food sources of employed foragers according to Equations (4) and (5), and generate new food sources by Equation (3); update the food sources if the new one is better than the old one; if there are some food sources which need to be given up (cannot be optimized after Limit times searches); the corresponding bees become the scouts, and generate new sources by Equation (2); increase times *t* = *t* + 1, go to step S2;S4: output the optimized results.End

#### 2.2.4. Bat Algorithm (BA)

Yang [8] presented the BA algorithm that is based on the echolocation behavior of bats. Suppose the frequency of a sound wave is $Freq\in \left[Freq_{min},Freq_{max}\right]$, $Freq_{min}$ and $Freq_{max}$ lower and upper bound, respectively. The sound intensity and pulse emissivity are defined as $A\in \left[A_{min},A_{max}\right]$ and r, respectively; the individuals update their positions by Equation (6).

(6) $$Freq_{i}=Freq_{min}+\left(Freq_{max}-Freq_{min}\;\right)*\beta_{i}\;v_{i}\left(t+1\right)=v_{i}\left(t\right)+\left(x_{i}\left(t\right)-p_{g}\left(t\right)\right)*Freq_{i}\;x_{i}\left(t+1\right)=x_{i}\left(t\right)+v_{i}\left(t+1\right)$$

where $\beta_{i}$ is the random number uniformly distributed in the range [0,1]. The bat generates a new solution by Equation (7),

(7) $$x_{new}=x_{old}+\varepsilon *\bar{A}\;$$

where ε is the random number in the range [−1, 1], $\bar{A}\;$is the average sound intensity of all the bats. $A_{i}$ and $r_{i}$ are updated by Equations (8) and (9):

(8) $$A_{i}\left(t+1\right)=\alpha *A_{i}\left(t\right)\;$$

(9) $$r_{i}\left(t+1\right)=r_{i}^{0}*\left[1-exp\left(-\gamma *t\right)\right]\;$$

where α and γ are two constants, $r_{i}^{0}\;$is the initialized value of r, the steps of the BA algorithm are described as Algorithm 4.
**Algorithm 4** BAInput: the parameters *M*, *N*, δ, Freq, A, α and γBeginS1: initialize *M* individuals $x_{i}\left(t\right)\;$randomly, 0<i≤M, iterative times *t* = 1;S2: compute f(i), 0<i≤M, update $p_{g}\left(t\right)$; if it satisfies (*t* > *N* or precision ≤δ), then go to step S4; otherwise, go to step S3;S3: update Freq,$\;x_{i}\left(t\right)$ and $v_{i}\left(t\right)$ by Equation (6), generate a random number $rand_{1}$, if $rand_{1}>r$, the bat with the global optimum generates a new solution by Equation (7). Bats generate new solutions randomly, and BA generates a random number$\;rand_{2}$; if $rand_{2}<A$ and the new solution is better than the old one, BA updates the corresponding position by Equation (7), and updates $A_{i\;}$and $r_{i\;}$ by Equations (8) and (9); iterative times *t* = *t* + 1, go to step S2;S4: output the optimized results.End

#### 2.2.5. Immune Algorithm (IA)

In 1958, Burnet [34] presented clonal selection theory, and Bersini [10] first used an artificial immune system to solve the discrete problem. Generally speaking, the common immune algorithm (IA) adopts the learning strategy similar to GA, while IA uses the affinity to guide the searching process [35]. The affinity is defined by information entropy; the average information entropy H(i,j) of antibodies i and j is described as follows.

(10) $$H\left(i,j\right)=\frac{1}{D}\overset{D}{\underset{l=1}{\sum }}H_{l}\left(i,j\right)\;$$

where $H_{l}\left(i,j\right)=\overset{s}{\underset{z=1}{\sum }}-p_{zl}log\;p_{zl}$, indicates the information entropy of the *l^th^* bit of genes for antibodies i and j. $\;p_{zl}$ is the probability of regarding the *l^th^* bit of genes for antibodies i and j as the gene letter $K_{z}$*,* $K_{z}\in \left\{K_{1},K_{2},\ldots ,K_{s}\right\}$; s is the number of gene letters. The affinity between antibodies i and j reflects the similarity of two antibodies, which is defined as follows.

(11) $$A_{i,j}=\frac{1}{1+H\left(i,j\right)}\;$$

The concentration of antibodies reflects the diversity of the whole population. The density of the *i^th^* antibody is described as follows.

(12) $$Con_{i}=\frac{1}{M}\overset{M}{\underset{j=1}{\sum }}C_{ij}\;C_{ij}=\{\begin{array}{c} 1\\ 0 \end{array}\;\;\begin{array}{c} A_{i,j}\geq h_{1}\\ A_{i,j}<h_{1} \end{array}\;,j=1,2,\ldots ,M$$

where $h_{1}$ is the threshold of the affinity. The activity degree refers to the comprehensive ability of the antibody to respond to antigen and be activated by other antibodies; generally, the antibody with large affinity and small concentration will have a large activity degree. The activity degree of the *i^th^* antibody is defined as follows.

(13) $$Act_{i}=\{\begin{array}{c} \frac{fit_{i}\left(1-Con_{i}\right)}{\sum_{i=1}^{M}fit_{i}*Con_{i}},\;\;Den_{i}\geq h_{2}\\ \frac{fit_{i}}{\sum_{i=1}^{M}fit_{i}*Con_{i}},\;\;Den_{i}<h_{2} \end{array}\;$$

where $fit_{i}$ is the fitness value of the *i^th^* antibody, $Con_{i}$ is the concentration of the *i^th^* antibody, and $h_{2}$ is the threshold of antibody density. The steps of IA are described as Algorithm 5.
**Algorithm 5** IAInput: the parameters *M*, *N*, δ,$\;P_{c}$, $P_{m}$BeginS1: initialize *M* antibodies $x_{i}\left(t\right)$ randomly, 0<i≤M, iterative times *t* = 1;S2: compute f(i) ,0<i≤M, update $p_{g}\left(t\right)$; if it satisfies (*t* > *N* or precision ≤δ), then go to step S4; otherwise, go to step S3;S3: compute the affinity, the concentration and activity degree according to Equations (11), (12) and (13); the selection operation is executed by the roulette method to choose antibodies with large activity degree, and then execute crossover and mutation operations according to the probabilities $P_{c}$ and $P_{m}$, respectively; iterative time *t* = *t* + 1, go to step S2;S4: output the optimized results.End

#### 2.2.6. Firefly Algorithm (FA)

The FA [9] algorithm was proposed by Yang Xin-She, and its main operations are the update of firefly luminance I, the computation of firefly attraction degree β and the update of firefly position. Suppose the attraction factor is γ, maximum luminance is $I_{0}$, the maximum attraction degree is $\beta_{0}$ and the step factor is step. The luminance is defined as Equations (14) and (15).

(14) $$I=I_{0}*exp\left(-\gamma *r_{i,j}\right)\;$$

(15) $$r_{i,j}=\sqrt{\overset{D}{\underset{k=1}{\sum }}{\left(x_{i,k}-x_{j,k}\right)}^{2}}\;$$

The attraction degree is described as Equation (16).

(16) $$\beta \left(r_{i,j}\right)=\beta_{0}*exp\left(-\gamma *r_{i,j}^{2}\right)\;$$

Equation (17) defines the position updating of the *i^th^* firefly when it moves toward the *j^th^* firefly. Here, $x_{i}\left(t\right)$ and $x_{j}\left(t\right)$ are the positions of the *i^th^* firefly and the *j^th^* firefly, respectively, at the iteration *t*. For convenience, we use $x_{i}\left(t\right)$ to represent the *i^th^* firefly.

(17) $$x_{i}\left(t+1\right)=x_{i}\left(t\right)+\beta \left(r_{i,j}\right)*\left(x_{j}\left(t\right)-x_{i}\left(t\right)\right)+step*\varepsilon_{i}\;$$

where $\varepsilon_{i}$ is the random number that follows Gaussian distribution or uniform distribution. The steps of FA are described as Algorithm 6.
**Algorithm 6** FAInput: the parameters *M*, *N*, δ, $I_{0}$, $\beta_{0}$ and stepBeginS1: initialize *M* individuals $x_{i}\left(t\right)\;$randomly, 0<i≤M, iterative times *t* = 1;S2: compute f(i),0<i≤M, if *t* > 1 and the new position is better than the old one, update $x_{i}\left(t\right)$ and $p_{g}\left(t\right)$; if it satisfies (*t* > *N* or precision ≤δ), then go to step S4; otherwise, go to step S3;S3: for the *i^th^* firefly $x_{i}\left(t\right)$, FA searches another firefly (suppose the *j^th^* firefly $x_{j}\left(t\right)$, and i≠j) in the population that has the luminance calculated with Equation (14). If the luminance of $x_{j}\left(t\right)$ is larger than that of $x_{i}\left(t\right)$, $x_{i}\left(t\right)$ moves toward $x_{j}\left(t\right)$ by Equation (17), iterative times *t* = *t* + 1; go to step S2;S4: output the optimized results.End

#### 2.2.7. Cuckoo Search (CS) Algorithm

The CS [7] algorithm was also put forward by Yang Xin-She, based on the brood parasitism of certain cuckoos species and the Lévy flights characteristics. The CS algorithm follows three idealized rules: (1) each cuckoo lays one egg at a time and dumps its egg in the randomly selected nest; (2) the best nest with the highest quality of eggs will carry over to the next generations; (3) the number of available host nests is fixed, and the egg laid by a cuckoo would be discovered by the host bird with a probability $p_{a}\in \left[0,1\right]$, that is, the fraction $p_{a}$ of M nests would be replaced by the new nests. The *i^th^* individual updates its host nest $x_{i}\left(t\right)$ by Equation (18),

(18) $$x_{i}\left(t+1\right)=x_{i}\left(t\right)+\alpha ⊗Levy\left(\lambda \right)\;$$

where α is the scaling factor of step size and α usually equals 1. The product ⊗ means entrywise multiplications and Levy*(*λ*)* indicates that the Lévy flight draws from the Lévy distribution. It is difficult to satisfy the real Lévy distribution, and Equation (19) is usually used to approximate Lévy flight:

(19) $$s=\frac{u}{{\left|v\right|}^{\frac{1}{\beta }}}\;$$

where u and v follow the Guassian distribution, $u\sim N\left(0,\sigma^{2}\right),\;v\sim N\left(0,1\right),\;\sigma ={\left\{\frac{\Gamma \left(1+\beta \right)sin\left(\frac{\pi \beta }{2}\right)}{\beta \Gamma \left(\frac{1+\beta }{2}\right)2^{\frac{\beta -1}{2}}}\right\}}^{\frac{1}{\beta }}$, β=1.5. Some cuckoo eggs may be found and discarded by the host, and the probability of abandonment is$\;p_{a}$. When a cuckoo egg is abandoned, the cuckoo needs to find a new boarding site and update it with the following Equation (20):

(20) $$x_{i}\left(t+1\right)=x_{i}\left(t\right)+\alpha s⊗H\left(p_{a}-\epsilon \right)⊗\left(x_{j}\left(t\right)-x_{k}\left(t\right)\right)\;$$

where $x_{j}\left(t\right)$ and $x_{k}\left(t\right)$ are two different solutions selected randomly by random permutation, *H* () is a Heaviside function, ϵ is a random number drawn from a uniform distribution, *s* is the step size that is defined as a random number in the scope of (0, 1) and α is a scaling factor of step size. The steps of the CS algorithm are described as Algorithm 7.
**Algorithm 7** CSInput: the parameters *M*, *N*, δ, α and $p_{a}$BeginS1: initialize *M* host nests $x_{i}\left(t\right)$ randomly, 0<i≤M, iterative times *t* = 1;S2: compute f(i),0<i≤M, update$\;p_{g}\left(t\right)$; if it satisfies (*t* > *N* or precision ≤δ), then go to step S4; otherwise, go to step S3;S3: choose a cuckoo randomly to generate a new solution by Equation (18); choose a nest among *M* individuals randomly; if the new solution is better than the chosen nest, replace it; a fraction ($p_{a}$) of the worst nests are replaced by the new nests by Equation (20), iterative times *t* = *t* + 1; go to step S2;S4: output the optimized results.End

#### 2.2.8. Differential Evolution (DE) Algorithm

The DE [4] algorithm, proposed by Storn, has three operations: mutation, crossover and selection, but is different from the GA algorithm. The main differences are: (1) in GA, two sub-individuals are generated by crossing two parent individuals, whereas in DE, new individuals are generated by perturbing the different vectors of several individuals; (2) in GA, the progeny individual replaces the parent individual with a certain probability, while in DE, the individual is only updated when the new individual is better than the old one. More specifically, the basic strategy of DE can be described as follows.

1. Mutation operation

Each individual $x_{i}\left(t\right)$ can execute the mutation operation according to Equation (21),

(21) $$v_{i,j}\left(t\right)=x_{r_{1},j}\left(t\right)+F\left(x_{r_{2},j}\left(t\right)-x_{r_{3},j}\left(t\right)\right)\;$$

where $x_{r_{1}}\left(t\right)$,$\;x_{r_{2}}\left(t\right)\;$and $x_{r_{3}}\left(t\right)$ are three individuals selected randomly from the whole population,$\;r_{1}\neq r_{2}\neq r_{3}\neq i$ and F∈[0, 2] is the mutation factor.

2. Crossover operation

The crossover individual $u_{i}\left(t\right)=\left(u_{i,1}\left(t\right),u_{i,2}\left(t\right),\ldots ,u_{i,D}\left(t\right)\right)$ can be generated by the mutation individual $v_{i}\left(t\right)$ and its parent individual $x_{i}\left(t\right)$, as described in Equation (22),

(22) $$u_{i,j}\left(t\right)=\{\begin{array}{cc} v_{i,j}\left(t\right) & if\;rand\;\leq CR\;or\;j=j\_rand\\ x_{i,j}\left(t\right) & if\;rand>CR\;and\;j\neq j\_rand \end{array}\;$$

where rand is a random number in the range [0, 1], CR is the crossover factor and it is a constant in the range [0, 1]; j_rand is an integer selected randomly from the range [1, D].

3. Selection operation

The DE algorithm adopts the “greedy” strategy; the next-generation individual is chosen between parent individual $x_{i}\left(t\right)$ and the crossover individual $u_{i}\left(t\right)$, which has the better fitness value, as described in Equation (23).

(23) $$x_{i}\left(t\right)=\{\begin{array}{cc} x_{i}\left(t\right) & if\;f\left(x_{i}\left(t\right)\right)\;\mathrm{is}\;\mathrm{better}\;\mathrm{than}\;f\left(u_{i}\left(t\right)\right)\\ u_{i}\left(t\right) & \mathrm{otherwise} \end{array}\;$$

The steps of the DE algorithm are described as Algorithm 8.
**Algorithm 8** DEInput: the parameters *M*, *N*, δ, F and *CR*BeginS1: initialize *M* individuals $x_{i}\left(t\right)\;$randomly, 0<i≤M, iterative times *t* = 1;S2: compute f(i), 0<i≤M, update$\;p_{g}\left(t\right)$; if it satisfies (*t* > *N* or precision ≤δ), then go to step S4; otherwise, go to step S3;S3: execute mutation operation by Equation (21), execute crossover operation by Equation (22) and execute selection operation by Equation (23), iterative times *t* = *t* + 1; go to step S2;S4: output the optimized results.End

#### 2.2.9. Gravitational Search Algorithm (GSA)

The GSA [12] algorithm was proposed by Esmat Rashedi which is based on the law of gravity and mass interactions. On the *t^th^* iteration, the force acting on particle $x_{i}\left(t\right)$ from particle $x_{j}\left(t\right)$ is defined as Equation (24),

(24) $$F_{i,j}^{d}\left(t\right)=G\left(t\right)*\frac{I_{pi}*I_{aj}}{R_{i,j}\left(t\right)+\varepsilon }*\left(x_{j,d}^{t}-x_{i,d}^{t}\right)\;$$

where $I_{pi}\;$is the active gravitational mass related to the particle$\;x_{j}\left(t\right)$, $I_{aj}\;$is the passive gravitational mass related to the particle $x_{i}\left(t\right)$, ε is a small constant, G(t) is the gravitational constant at iteration *t*, which is defined as Equation (25):

(25) $$G\left(t\right)=G_{0}*e^{-\alpha *t/N}\;$$

where $G_{0}=100$ and α=20. $R_{i,j}\left(t\right)$ is the Euclidian distance between two particles $x_{i}\left(t\right)$ and $x_{j}\left(t\right)$, described as follows.

(26) $$R_{i,j}\left(t\right)=\sqrt{\overset{D}{\underset{k=1}{\sum }}{\left(x_{i,k}^{t}-x_{j,k}^{t}\right)}^{2}}\;$$

On the *t^th^* iteration, the total force acting on the particle $x_{i}\left(t\right)\;$in the dimension *d* is defined as Equation (27),

(27) $$F_{i}^{d}\left(t\right)=\overset{M}{\underset{j=1,j\neq i}{\sum }}rand_{j}*F_{i,j}^{d}\left(t\right)\;$$

where $rand_{j}$ is a random number in the interval [0, 1], the acceleration of the particle $x_{i}\left(t\right)$ in the dimension *d* is defined as Equation (28):

(28) $$a_{i}^{d}\left(t\right)=\frac{F_{i}^{d}\left(t\right)}{I_{ii}\left(t\right)}\;$$

where $I_{ii}\left(t\right)$ is the inertial mass of particle $x_{i}\left(t\right)$; one particle updates its velocity and position according to its acceleration, as described in Equation (29).

(29) $$v_{i,d}\left(t+1\right)=rand_{i}*v_{i,d}\left(t\right)+a_{i}^{d}\left(t\right)\;x_{i,d}\left(t+1\right)=x_{i,d}\left(t\right)+v_{i,d}\left(t+1\right)$$

The GSA algorithm updates the gravitational and inertial masses by Equation (30).

(30) $$I_{ai}=I_{pi}=I_{ii}=I_{i},\;i=1,2,\ldots ,M\;i_{i}\left(t\right)=\frac{f_{i}\left(t\right)-worst\left(t\right)}{best\left(t\right)-worst\left(t\right)}\;I_{i}\left(t\right)=\frac{i_{i}\left(t\right)}{\sum_{j=1}^{M}i_{j}\left(t\right)}$$

where $f_{i}\left(t\right)$ is the fitness value of the particle $x_{i}\left(t\right)$, best(t) and worst(t) represent the best and the worst fitness value among all the particles, respectively; they are defined as Equation (31).

(31) $$best\left(t\right)=\begin{array}{c} best\\ j\in \left\{1,2,\ldots ,M\right\} \end{array}f_{j}\left(t\right)\;worst\left(t\right)=\begin{array}{c} worst\\ j\in \left\{1,2,\ldots ,M\right\} \end{array}f_{j}\left(t\right)$$

The steps of the GSA algorithm are described as Algorithm 9.
**Algorithm 9** GSAInput: the parameters *M*, *N*, δ, $G_{0}$, αBeginS1: initialize *M* particles $x_{i}\left(t\right)\;$randomly, 0<i≤M, iterative times *t* = 1;S2: compute f(i), 0<i≤M, update $p_{g}\left(t\right)$; if it satisfies (*t* > *N* or precision ≤δ), then go to step S4; otherwise, go to step S3;S3: update G(t) by Equation (25), update best(t) and worst(t) by Equation (31), update $I_{i}\left(t\right)\;$by Equation (30), calculate $F_{i}^{d}\left(t\right)\;$by Equation (27), compute $a_{i}^{d}\left(t\right)$ by Equation (28), update $v_{i,d}\left(t+1\right)\;$and $x_{i,d}\left(t+1\right)$ by Equation (29), iterative times *t* = *t* + 1; go to step S2;S4: output the optimized results.End

#### 2.2.10. Grey Wolf Optimizer (GWO)

Grey wolf optimizer was proposed in 2014, in which four types of grey wolves are employed for simulating the leadership hierarchy, including alpha (α), beta (β), delta (δ) and omega (ω). The α wolf is called the dominant wolf because his/her orders should be followed by the pack, the β wolf is probably the best candidate to be the α wolf in case the latter passes away or becomes very old, the δ wolf should respect the α but commands the other lower-level wolves as well, the lowest ranking grey wolf is ω and it plays the role of scapegoat. Grey wolves encircle prey during a hunt. In GWO, the encircling behavior can be described by the following equations:

(32) $$D=\left|C*x_{p}\left(t\right)-x\left(t\right)\right|\;$$

(33) $$x\left(t+1\right)=x_{p}\left(t\right)-A*D\;$$

where A and C are coefficient vectors, $\;x_{p}\left(t\right)$ is the position vector of the prey (the solution) and x(t) is the position vector of a grey wolf. A and C are calculated as follows,

(34) $$A=2*a*r_{1}-a\;$$

(35) $$C=2*r_{2}\;$$

where a is linearly decreased from 2 to 0 over the course of iterations, $r_{1}$ and$\;r_{2}\;$are random values in the scope of [0, 1]. In order to mathematically simulate the hunting behavior of grey wolves, GWO supposes that α, β and δ have better knowledge about the potential location of prey; it saves the first three best solutions obtained so far and oblige the other wolves to update their positions according to the position of the best search wolves, described as follows.

(36) $$D_{\alpha }=\left|C_{1}*x_{\alpha }\left(t\right)-x\left(t\right)\right|\;$$

(37) $$D_{\beta }=\left|C_{2}*x_{\beta \;}\left(t\right)-x\left(t\right)\right|\;$$

(38) $$D_{\delta }=\left|C_{3}*x_{\delta \;}\left(t\right)-x\left(t\right)\right|\;$$

(39) $$x_{1}=x_{\alpha }-A_{1}*D_{\alpha }\;$$

(40) $$x_{2}=x_{\beta }-A_{2}*D_{\beta }\;$$

(41) $$x_{3}=x_{\delta \;}-A_{3}*D_{\delta \;}\;$$

(42) $$x\left(t+1\right)=\frac{x_{1\;}+x_{2\;}+x_{3\;}}{3}\;$$

$\mathrm{Here},\;A_{1}$*,*$\;A_{2}$*,*$\;A_{3}\;$and $C_{1}$*,*$\;C_{2}$*,*$C_{3}$ are the coefficients that are generated by different random values. The steps of the GWO algorithm are described as Algorithm 10.
**Algorithm 10** GWOInput: the parameters *M*, *N*, δBeginS1: initialize *M* individuals $x_{i}\left(t\right)\;$randomly, 0<i≤M, iterative times *t* = 1;S2: compute f(i), 0<i≤M, rank the solutions and find the current top three best wolves:$\;x_{\alpha }\left(t\right)$,$\;x_{\beta }\left(t\right)$ and$\;x_{\delta }\left(t\right)$; if it satisfies (*t* > *N* or precision ≤δ), then go to step S4; otherwise, go to step S3;S3: update the solution of each individual via Equation (42), update coefficients *a*, *A* and *C*, iterative times *t* = *t* + 1; go to step S2;S4: output the optimized results.End

#### 2.2.11. Harmony Search (HS) Optimization

Harmony search (HS) optimization algorithm [13] is inspired by improvization in music-playing. In a band, all players adjust their pitch to achieve a wonderful harmony. In the process of global optimization, all decision variables constantly adjust their own values to make the objective function achieve global optimization. A harmony memory (*HM*) with size given by the parameter *HMS* (harmony memory size) stores the best harmony vectors during the optimization. A new harmony vector $x_{i}^{\prime }\left(t\right)=\left(x_{1}^{\prime }\left(t\right),x_{2}^{\prime }\left(t\right)\ldots ,x_{D}^{\prime }\left(t\right)\right)$ is generated from the *HM* based on memory considerations, pitch adjustments and randomization. For $x_{i}^{\prime }\left(t\right)$*,* i=1,2,…,D*,* it chooses a new value using the parameter of harmony memory considering rate (HMCR), which varies between 0 and 1 as follows.

(43) $$x_{i}^{\prime }\left(t\right)=\{\begin{array}{cc} x_{i}^{j}\left(t\right)\;\;\;j\in \left\{1,2,\ldots ,HMS\right\} & r_{1}\leq HRCM\\ x_{i}^{\prime }\left(t\right)\in X_{i} & else \end{array}\;$$

Equation (43) indicates that $x_{i}^{\prime }\left(t\right)$ would be updated from *HM* if a random-generated number $r_{1}$ is less than or equal to *HMCR*, and would be randomly chosen one feasible value from $X_{i}$, which is the definition space of the *i^th^* dimensional variable. Every component $x_{i}^{\prime }\left(t\right)$ is examined to determine whether it should be pitch-adjusted. The procedure uses the parameter of pitch adjusting rate (*PAR*) that sets the rate of adjustment for the pitch chosen from the *HM* as follows.

(44) $$x_{i}^{\prime }\left(t\right)=\{\begin{array}{cc} x_{i}^{\prime }\left(t\right)+\alpha & r_{2}\leq PAR\\ x_{i}^{\prime }\left(t\right) & \mathrm{otherwise} \end{array}\;$$

Here, $r_{2}$ is a random-generated number and α is defined in Equation (45):

(45) α=BW∗u(−1,1) 

where BW is an arbitrary distance band width for continuous design variable, and u(−1,1) is a uniform distribution between −1 and 1. The steps of the HS algorithm are described as Algorithm 11.
**Algorithm 11** HSInput: the parameters *M* (or *HMS*), *N*, δ, *HMCR*, *PAR* and *BW*BeginS1: initialize the harmony memory filled with *M* solutions that are randomly generated, iterative times *t* = 1;S2: compute f(i), 0<i≤M, update $p_{g}\left(t\right)$; if it satisfies (*t* > *N* or precision ≤δ), then go to step S4, otherwise, go to step S3;S3: generate new harmony according to Equations (43) and (44); if it is better than one harmony in *HM*, replace it, iterative times *t* = *t* + 1; go to step S2;S4: output the optimized results.End

## 3. Theoretical Comparison and Analysis of the 11 NIOAs

### 3.1. Common Characteristics

As shown in Section 2, although the NIOAs simulate different population behaviors, all of them are the iterative methods and have some common characteristics which satisfy the Reynolds model [36] and this model describes the basic rules for the aggregation motion of the simulated flock created by a distributed behavioral model.

**1. Randomicity**. Randomicity is the uncertainty of an event with a certain probability and can enhance the global search capability of individuals. All the 11 NIOAs initialize individuals randomly, which can cover the space as large as possible, some other mechanisms have been adopted in them which can enhance the exploration and exploitation abilities, such as the mutation operators $m_{o}$ in GA, IA and DE, the random parameters $rand_{1}$ and $rand_{2}$ in PSO, rand(0,1) and φ in ABC, $\beta_{i}\;$and ε in BA, $\varepsilon_{i}\;$in FA, Lévy flight in CS, rand and$\;rand_{j}$ in DE, $rand_{j}\;$and $rand_{i}$ in GSA, $r_{1},\;r_{2}$,$A_{1},\;A_{2},\;A_{3}\;$and $C_{1}$,$\;C_{2},C_{3}\;$in GWO, u(−1,1) in HS, etc.

**2. Information Interactivity**. The individuals in the NIOAs should exchange information directly or indirectly, which can increase the probability of obtaining the global optimum. For instance, GA, IA and DE adopt the crossover operator $c_{o}$ to exchange information; for PSO, each particle utilizes the global optimum $p_{g}\left(t\right)$ to update its position; employed foragers or onlookers in ABC update their velocities $v_{i,d}\left(t+1\right)$ using another different position $x_{j,d}\left(t\right)$; bats in BA use global optimum $p_{g}\left(t\right)$ to update their positions; in FA, firefly $x_{i}\left(t\right)$ moves toward $x_{j}\left(t\right)$ through mixing the positions information of $x_{i}\left(t\right)$ and $x_{j}\left(t\right)$; as to GSA, the force $F_{i,j}^{d}\left(t\right)$ is computed according to the positions of particles $x_{i}\left(t\right)$ and $x_{j}\left(t\right)$, which are used to update the position of each particle; the wolf in GWO updates its position according to the positions of wolves α, β and δ; and at last but not least, a new harmony in HS is generated from *HM*.

**3. Optimality**. The individuals in the NIOAs move toward the global best solution through different mechanisms of information exchange. For example, the good genes in GA and DE are chosen as the next generation through the operators $s_{o}$,$\;c_{o}$ and $m_{o}$; particles in PSO update their positions, influenced by the local optimum $p_{i}\left(t\right)$ and the global optimum$\;p_{g}\left(t\right)$; onlookers in ABC choose the food sources that have better fitness than the old one, the bat in BA generates a new solution and updates its position only if the new solution is better than the old one; the good antibodies in IA would save in a memory database to participate in the next iteration; as to FA, one firefly moves toward the fireflies that have larger luminance; CS replaces solutions only when the new one is better than the chosen solution and a fraction of worse solutions will be replaced by the newly generated solutions; in GSA, the gravitational and inertial masses are calculated by the fitness evaluation and the better individuals have higher attractions and walk more slowly; wolves in GWO update their positions according to the positions of wolves α, β and δ, which have better solutions in the wolf population; and a new harmony vector in HS can replace the old harmony in HM only if the new harmony is better than the old one.

In addition to the aforementioned common characteristics of theoretical implementation, these common NIOAs are varied to different versions to handle different problems, including combinational optimization problems (COPs) and multi-objective optimization problems (MOOPs). Similar variant methods are adopted to improve the optimization performance of NIOAs, for example, adaptive technology, fuzzy theory, chaos theory, quantum theory and hybridization technology. The classic articles about the above work are listed in Section 3.2, which provides a comprehensive summary of the 11 different NIOAs.

### 3.2. Variant Methods of Common NIOAs

The summarization of variant methods in other survey work [26,27,28,29,30] are fragmented; in this work, we systematically summarize the popular variants of the 11 common NIOAs, and the popular methods are described in Table 2; because of the massive papers, the summarized literature are the state-of-the-art or representative papers, and the superscript is the citation times from the *Web of Science* and *Scopus* databases (by the end of 13 October 2020).

As described in Table 2, the following observations have been made:

(1) All of the 11 common NIOAs have their versions of handling MOOPs and COPs and have been improved through adopting various adaptive strategies, for example, automatically tuning parameters.

(2) Some classic mathematical and physical theories have been used to enhance the performance of NIOAs, such as fuzzy theory [133], chaos theory [56] and quantum theory [48,132], and the exploration and exploitation abilities of NIOAs.

(3) Hybridization is another major method of improving the performance of NIOAs, which make combinational use of the advantages of multiple NIOAs, for instance, GA-IA [38], GA-PSO [47], PSO-ABC [134], PSO-BA [135], BA-DE [76], FA-DE [84], IA-DE [92], CS-GA [100], ABC-DE [108], DE-PSO [57,109], PSO-GSA [117] and PSO-EDAs [58]. In addition, some other methods are also hybridized to improve the performance of NIOAs, such as the Taguchi method [43,60], the Gradient algorithm [59] and the Nelder–Mead simplex method [68].

In general, the proposers maintain the view that a hybridization mechanism can make full use of the advantages of some NIOAs to overcome the disadvantages of the other NIOAs. Following are some examples of this view: DE has the abilities of strong global exploration and rapid convergence; PSO is easy to fall into the local optimum; ABC has slow convergence speed and is poor to balance the exploration and exploitation; BA and FA have a good performance on low dimensional problems, but are not good at solving high dimensional and nonlinear problems; GWO has a poor diversity of population and slow convergence, etc. Although many excellent hybrid NIOAs have been proposed (as mentioned above), what we have to admit is a confusing trend and route of NIOAs enhancement: in order to improve some NIOAs, the “proposers” always claimed that the selected NIOAs have a series of shortcomings, such as being easy to fall into local optimum, slow convergence and not being good at solving high-dimensional problems, and the proposed hybrid methods can improve the performance of the selected NIOAs. It is very likely that, without being fully aware of the merits and demerits of the common NIOAs, a so-called “novel” algorithm is only a mixture of NIOAs. The performance of such hybrid NIOAs, including convergence speed, ability to solve high-dimensional problems and algorithm accuracy, need to be verified with comprehensive experiments. Section 4 will give a systematic comparison and analysis of the performance of the 11 common NIOAs.

### 3.3. Differences

From our observation, there are three important aspects that influence the performance of NIOAs, including the method of parameter tuning, the learning strategy and the topological structure of the population. As the original 11 NIOAs adopt empirical parameters, we will not discuss the problem of parameter-tuning in this paper. However, we will address the sensitivity of the algorithms to their parameters setting in Section 4.2. In this section, we discuss the differences in learning strategies, the topology structures, the time complexities and the interactions of algorithmic components for the 11 NIOAs.

**1. Learning strategies**

Each NIOA has its own learning strategy. GA exchanges information among genes through selection, crossover and mutation operators, while IA updates antibodies according to the conception of activity degree. In GA, the selection operation is executed by the probability $\vartheta_{1}$, while in IA, the selection operation is executed by the roulette method to choose antibodies with large activity degree; the main differences between the GA and DE algorithms were described in Section 2.2.8. In PSO, $x_{i}\left(t\right)$ is updated by its local best optimum$\;p_{i}\left(t\right)$ and the global best optimum $p_{g}\left(t\right)$ of the whole population (see Equation (1)), and in BA, the updating methods of positions and velocities have some similarities to those of PSO. To some extent, BA can be regarded as a combination of the global optimum$\;p_{g}\left(t\right)$ in PSO with an extensive local search method, which is described by the sound intensity $A_{i}\left(t\right)$ and the pulse emissivity $r_{i}\left(t\right)$ (see Equations (7)–(9)); in FA, if the attraction factor γ (see Equation (17)) tends to zero, it is a special case of PSO; according to Equations (1) and (29), GSA also can be regarded as a variant of PSO. The difference among them is that velocity updating of the former is influenced by local optimum $p_{i}\left(t\right)$ and global optimum $p_{g}\left(t\right)$, while those of the latter is affected by all the other individuals (named as the total force); in CS, each cuckoo randomly updates its solution by Lévy flight (see Equation (18)), and new solutions are generated to replace the worst solutions through learning from other individuals. The harmony in HS has interactions with the best individuals (solutions in harmony memory) according to certain probability, which updates solution based on memory considerations, pitch adjustments and randomization (see Equations (43) and (44)); compared to the other nine NIOAs, GWO and ABC have hierarchical evolution mechanisms and the roles can be changed dynamically according to the quality of individuals’ solutions. Wolves in GWO learn from the current top three best wolves α,β and δ (see Equation (42)), and onlookers in ABC learn from the selected employed foragers that found food sources (see Equation (4)).

**2. Topological structures**

**Figure 4:**
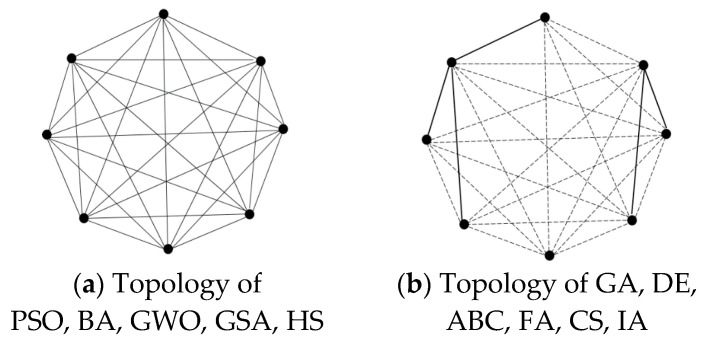
Topologies of 11 compared NIOAs.

According to the scope of interaction among individuals, the topologies of the 11 SOIAs can be roughly grouped into two categories: global neighborhood topology (GNT) and local neighborhood topology (LNT). In this paper, we consider the neighborhood topology in the period of each iteration of NIOAs, and thus we have the following observation and conclusions. GA is LNT, because it can only exchange information between two genes in each iteration; DE is also LNT for a similar reason; ABC can be regarded as LNT too because onlookers only follow certain employed foragers, and scouts generate new solutions randomly and have no interaction with other bees; FA belongs to LNT because a firefly moves towards another firefly that has larger luminance; CS is LNT because individuals either generate new solution independently (see Equation (18)) or produce new solutions and exchange information with other individuals (see Equation (20)); similar to GA, IA also is LNT because two antibodies exchange information through crossover factors. As each particle updates its position using $p_{g}\left(t\right)$, PSO is regarded as GNT; BA is classified as GNT for the same reason as PSO; GWO is also GNT because each wolf in GWO updates its position following the best three wolves; GSA belongs to GNT because each particle updates its position following the total force from all the other particles; HS updates solutions from HM that stored the best harmony vectors of the whole population, so it is GNT. The topologies of the 11 NIOAs are illustrated in Figure 4, where each circle represents an individual; the solid line represents those two individuals have information exchange in the current iteration and the dotted line indicates that two individuals maybe exchange information in the sense of probability during the whole evolution process.

GNT and LNT have their own advantages and disadvantages. Generally speaking, all individuals in GNT are connected to each other and attracted are to the global best solution of the whole population; its merits include rapid convergence and strong exploitation ability, while it is more likely to be confined at a local optimum; on the contrary, each individual in LNT only connects to several other individuals in its neighborhood and is attracted by the best position of the neighborhoods. LNT can make individuals search diverse regions of problem space and has a strong exploration ability, while it may have a slow convergence speed.

**3. The interactions of algorithmic components**

It is necessary to consider insights into the contribution of each component in NIOAs. The interactions of algorithmic components can reflect the core optimization power of the overall method [136]. According to the learning strategies and topological structures of NIOAs, the interactions of algorithmic components can be described. GA, IA and DE exchange information through three components: selection, crossover and mutation operators. For GA, selection and crossover are two main components, and information exchange happens between two genes in each iteration; mutation is executed in a low probability which can increase the diversity of the population. Owing to the local topology, GA has slow convergence. For IA, it uses affinity to guide the searching process. For DE, the mutation is the main operation and generates new solutions by perturbing the different vectors of several individuals. PSO updates the velocity of each particle using its historical and globally optimal solutions; the topology is a full connection, and information exchange is very fast, and thus it is easy to fall into local optimum. ABC has three components: employed foragers, scouts and onlookers. Employed foragers learn from other randomly selected individuals to update their velocity, onlooker finds solutions through sharing information with a specific employed forager and scouts can generate new solutions randomly. The topological structure of ABC is LNT, and the roles of bees can be changed dynamically. BA has a similar mechanism of exchanging information to those of PSO; it updates the velocity using the global optimum; its topology structure is GNT. FA updates its position by exchanging information with another firefly; it is regarded as a special case of PSO, but it belongs to LNT. The particle in GSA updates its velocity according to the acceleration, and the latter uses the total force from all the particles; thus, GSA belongs to GNT. GWO has four types of grey wolves, including alpha (*α*), beta (*β*), delta (*δ*) and omega (*ω*). GWO saves the top three best solutions (*α*, *β*, *δ*) obtained so far and obliges the other wolves to update their positions according to the position of the best three wolves. HS exchanges information with the best solutions (stored in harmony memory) according to a certain probability; it belongs to GNT.

**4. Time complexities analysis**

The time complexity of the 11 NIOAs is described in Table 3 below, where D, M and N represent the number of the dimensions of objective functions, M is the number of the individuals of each NIOA and N is the total iterative times, respectively, and has been defined in Table 1. In order to calculate the time complexity of individual operations in the NIOAs, we divide the NIOAs operations into various components and assign corresponding computational costs. Specifically, we use T_init_ to denote the computational cost of initialization, T_eval_ is the evaluation of a single solution and T_iter_ is the computational cost of one iteration of the main loop of the NIOAs, which is determined by the cost of the operations of updating solutions (T_upd_), evaluating solutions (T_e_) and the calculating statistics (T_stats_). For the 11 compared NIOAs, the computational cost of T_init_, T_eval_, T_e_ and T_stats_ have the same values, which are calculated as follows: T_init_ = D∙M, T_eval_ = D, T_e_ = M∙T_eval_, T_stats_ = M. Thus, the computational cost of one iteration is reckoned as T_iter_ = T_upd_ + T_e_ + T_stats_ and T_upd_ varies with the different NIOAs, the total computational cost of an NIOA (for example, PSO) is defined as T_PSO_ = T_init_ + T_iter_∙T. The time complexity of the 11 compared NIOAs is described in Table 3. From Table 3, we can see that the time complexity of PSO, GA, ABC, BA, CS, DE, GWO and HS is O(D∙M∙N); that of GSA and IA is O((D+M)∙M∙N) and FA O(D∙M^2^∙N). The actual running time given by the benchmark functions (discussed in Section 4.2.3) agrees with the above estimates.

## 4. Performance Comparison and Analysis for the 11 NIOAs

### 4.1. The Description of BBOB Test Functions

In order to evaluate the performances of the 11 NIOAs, 30 Black-Box Optimization Benchmarking (BBOB) functions are adopted as fitness functions, which were proposed in the 2017 IEEE Congress on Evolutionary Computation. The optimum values of the 30 BBOB functions (F1–F30) are from 100 to 3000 with the step of 100; they include unimodal functions, multimodal functions, hybrid functions and composition functions. For the sake of fairness, each algorithm is run 20 times independently, and the individual quantity of every algorithm is 50; all the BBOB functions are tested in low dimension (*D* = 10) and high dimension (*D* = 50) and the iterative times are 1500 and 15,000 when D equals 10 and 50, respectively. In order to analyze the sensitivity of the compared NIOAs to their parameter settings, we apply two groups of parameters for each of the 11 NIOAs. The first group of parameters is consistent with the parameters of the 11 original algorithms. The second group of parameters is different from the first group and is randomly selected according to the principles of the 11 NIOAs. For example, the mutation probability $\vartheta_{3}$ in GA should generally not be too large, and its value in the second group is set to 0.25. The parameters of the compared NIOAs are described in Table 4. For GA and IA in this work, we adopt the method of roulette wheel selection for the selection operators, the multi-point crossover and one-point crossover for the crossover operators and the basic mutation method for the mutation operators that chooses one or more genes to randomly change.

### 4.2. Performance Comparison and Analysis on Benchmark Functions

#### 4.2.1. The Comparison and Analysis on the Accuracy, Stability and Parameter Sensitivity

From the best fitness values (functions), we derive four kinds of criterion values, i.e., the best, the average, the worst and the standard deviation, to qualitatively indicate the effect of each algorithm for a given BBOB function under a certain dimension with a given group of parameters after 20 times of repeated experiments. They are denoted to be BEST, AVERAGE, WORST and STD. Tables S2–S7 of Supplementary Material B show the experimental results for the four kinds of criterion values with all the 30 BBOB functions under the first group of parameters when it is low dimension (=10). Tables S8–S13 of Supplementary Material C show the experiment results when it is high dimension (=50). The values in bold represent the algorithm of the 11 NIOAs with the best result.

Similarly, the experiment results for the four kinds of criterion values on the second group of parameters are presented in Tables S14–S19 of Supplementary Material D for the low dimension (=10) and Tables S20–S25 of Supplementary Material E for the high dimension (=50). Again, the values in bold represent the algorithm with the best result.

Based on the above experimental results, we count the number of times that these 11 NIOAs achieved a “good” result on the 30 functions, as described in Table S26 of Supplementary Material J. In order to objectively analyze the performance of the NIOAs, if the result of the BEST criterion is within 20% on the optimal solution, it is regarded as the “good” result; for the STD criterion, values are less than 50 of F1–F10, are less than 150 of F11–f20 and are less than 300 of F21–F30 are considered as “good” results. In order to compare the performance of 11 NIOAs, the results of Tables S2–S13 of Supplementary Materials B and C are briefed in Table 5, the results of Tables S14–S25 of Supplementary Materials D and E are briefed in Table 6. As described in Table 5 and Table 6, the bold number is the number of wins for the NIOAs on a specific criterion, and the corresponding winning functions are shown in the brackets followed. In addition, as described in Tables S27–S30 of Supplementary Material K, we also give the mean error (AVERAGE ± STD) values of the 11 NIOAs on the 30 BBOB functions, where the values in bold represent the algorithm having the best result.

In this section, we not only compare the accuracy and stability of different NIOAs, but also analyze the sensitivity of each selected NIOA by setting two groups of parameters for the compared NIOAs. It should be noted that it is impossible to find a super NIOA to solve all optimization problems, and it is more meaningful to design a NIOA for a specific problem. However, the experimental results may be helpful for researchers to understand the learning strategy and topology of these NIOAs. In addition, two groups of parameters are chosen to analyze the sensitivity of the 11 NIOAs to their parameters setting, which can roughly reflect the robustness of the compared NIOAs.

**1. Analysis of accuracy and stability**

We compare the 11 algorithms in terms of accuracy and stability under the two groups of parameters. The rankings of accuracy and stability of the compared NIOAs is roughly the same. Based on the experimental results of Table 5 and Table 6, and Table S121 of Supplementary Material J and Tables S122–S125 of Supplementary Material K, the following observations can be made:

(1) As described in Table S26, for the accuracy in low dimensional space, all the NIOAs can obtain good results on at least half of the BBOB functions, while in high dimensional space, the accuracy of all the NIOAs is much worse than that in the low dimensional space, only GSA, DE and CS obtain relatively good results. For the stability in low dimensional space, all the NIOAs can obtain good results on at least half of the BBOB functions, while in high dimensional space, the stability of all the NIOAs is much worse; only CS, DE, ABC and GSA obtain relatively good stability.

(2) As shown in Table 5 and Table 6, DE and CS can receive better solutions and stability compared to the other nine NIOAs for both groups of parameters. Especially, for the BEST criterion on the two groups of parameters, DE achieves the best results among the 11 NIOAs. DE and CS are the most stable algorithms among the 11 NIOAs for the different parameter settings. According to the mean error values of Tables S27–S30, DE and CS can receive obvious better results in low dimensional space compared with the other nine NIOAs, and in high dimensional space, CS, DE and GWO can obtain better mean error values than the other eight NIOAs.

**2. Analysis of the parameter sensitivity on the 11 NIOAs**

Undoubtedly, the optimization results of all the compared NIOAs are sensitive to their parameters settings, as described in Supplementary Materials B–E. In this work, in order to analyze the degrees of sensitivity for the compared NIOAs, we think that an NIOA is sensitive to its parameters setting if the difference of the two results on the same BBOB function under two groups of parameters is greater than one order of magnitude (one result value is 10 times greater than the other). The statistical results are shown in Table 7, and if the experimental results of certain functions differ by two orders of magnitude, two stars are marked on the corresponding function, etc. As described in Table 7, the following observations can be made:

(1) DE, CS, HS, GSA, GWO, FA, BA and IA are sensitive to their parameters setting on high dimensional space and are relatively insensitive in low dimensional space, which indicates that it should be careful to select the parameters of the above NIOAs when using them in high dimensional problems. Specifically, DE and HS are the most sensitive to their parameters setting on high dimensional space.

(2) ABC, PSO and GA are sensitive to their parameters setting both on high and low dimensional spaces, and PSO is the most sensitive to their parameters setting.

According to the above observations, we draw the following preliminary conclusions:

(1) The NIOAs, which have explicit learning strategy of solution update, can acquire better performance than the NIOAs with large randomness (for example, probability method) to learn from other individuals. For example, In GA, the progeny individual replaces the parent individual with a certain probability, while in DE, the individual is only updated when the new individual is better than the old one; the cuckoos in CS update their positions once the new solution generated by Lévy flights is better than the old one; while the individual in BA can be updated by the better one under the probability constraints $rand_{2}<A$, which means that it may not be updated by the better individual; IA executes crossover and mutation operations through choosing antibodies with large activity degree, but the computation of large activity has some uncertainties, for example, the design of the threshold $h_{1}$ in Equation (12) and $h_{2}$ in Equation (13). It seems that the algorithms can achieve better performance, which continuously and randomly generate new solutions and firmly learn from excellent individuals.

(2) With the increase of dimensional number, all the NIOAs become more sensitive to their parameters setting, which indicates that it is more difficult to choose a suitable set of parameters for NIOAs on high dimensional problems, except for DE and CS, GSA and GWO, which perform better in high dimensional space than the other seven NIOAs.

#### 4.2.2. The Efficiency Comparison and Analysis

For the sake of error elimination, we compute the average value of each iteration in 20 independent experiments and obtains the change curves of the global optimized fitness under 1500 and 15,000 iterations on 30 functions for the low and high dimensions, respectively. For the first group of parameters for compared NIOAs, when *D* equals 10, the convergent curves of 11 NIOAs on 30 BB functions are described in Figures S1–S30 of Supplementary Material F, and for the case of *D* =50, the corresponding curves are described in Figures S31–S60 of Supplementary Material G. For the second group of parameters, the corresponding convergent curves are described in Figure S61–S90 of Supplementary Material H and in Figures S91–S120 of Supplementary Material I, respectively. Based on these experimental results, the following observations can be made:

(1) FA and HS have the worst optimization efficiency for most of the 30 functions both on the low and high dimensions, because they either evolve solutions through adopting complete random strategies (see Equations (43) and (44)), for example, HS or learn from other individuals in local topology and are perturbed by a random factor (see Equation (17)), such as FA.

(2) With the increasing of iterative times, the curves of most compared NIOAs trend to be stable, while those of ABC and DE are the oscillatory curves for most of the 30 functions both on the low and high dimensions; the amplitude and frequency of oscillations of ABC are greater than those of DE, and they are larger in high dimensions than low dimension for the two algorithms, but from the whole iteration period, the optimization results of ABC and DE are gradually improved.

(3) PSO, GSA and GWO have the fast convergent speed for most of the 30 functions both on the low and high dimensions, because all of them adopt the explicit strategy of learning from the global best solution; that is, the individuals in these NIOAs learn firmly from the global optimum which leads to the rapid convergence. 

#### 4.2.3. The Comparison of Running Time

The running time of the 11 NIOAs on 30 BBOB functions are summarized in Table S31 of Supplementary Material L. Based on these data, the following observations can be made:

(1) DE and CS are the fastest algorithms on all the 30 functions for the dimension of 10 and 50, respectively. FA is the slowest algorithm both on the low and high dimensional spaces; its running time on the 30 functions is 1~2 orders of magnitude higher than the other 10 NIOAs. GSA has the second-worst running time for both dimensional spaces. The slowest running time of FA and GSA echoes their time complexity given in Table 3 of Section 3.3.

(2) PSO, GA, BAC, BA, GWO and HS are fast when *D* = 10, while in the high dimensional space, their running time is obviously longer than the low dimensional space. Thus, from the view of running time, the above algorithms are more suitable to low dimensional problems, DE and CS are suitable to the problems on both high and low dimensional spaces.

(3) For *D* = 10, the running time of FA is 20 times that of DE for almost all the functions; when *D* = 50, FA is 20 times slower than CS (the maximum is 35) for all the functions. Thus, the difference in running time for the NIOAs is very large, and hence it is important to select fast NIOAs for the optimization problems with the strict requirement of running time.

### 4.3. Statistical Tests for Algorithm Comparison

In this study, we consider two statistical tests: the Friedman test [137] and Nemenyi test [137]. A Friedman test is constructed to analyze the performance of the compared NIOAs. Table 8 provides the Friedman test statistics $F_{F}$ and the corresponding critical value in terms of each evaluation criterion. As shown in Table 8, the null hypothesis (that all of the compared algorithms will perform equivalently) was clearly rejected for each evaluation criterion at a significance level of α=0.05 for the experimental results in both 10 and 50 dimensional spaces. Consequently, we proceed to conduction of a post hoc test [137] in order to analyze the relative performance among the compared NIOAs.

**Figure 5:**
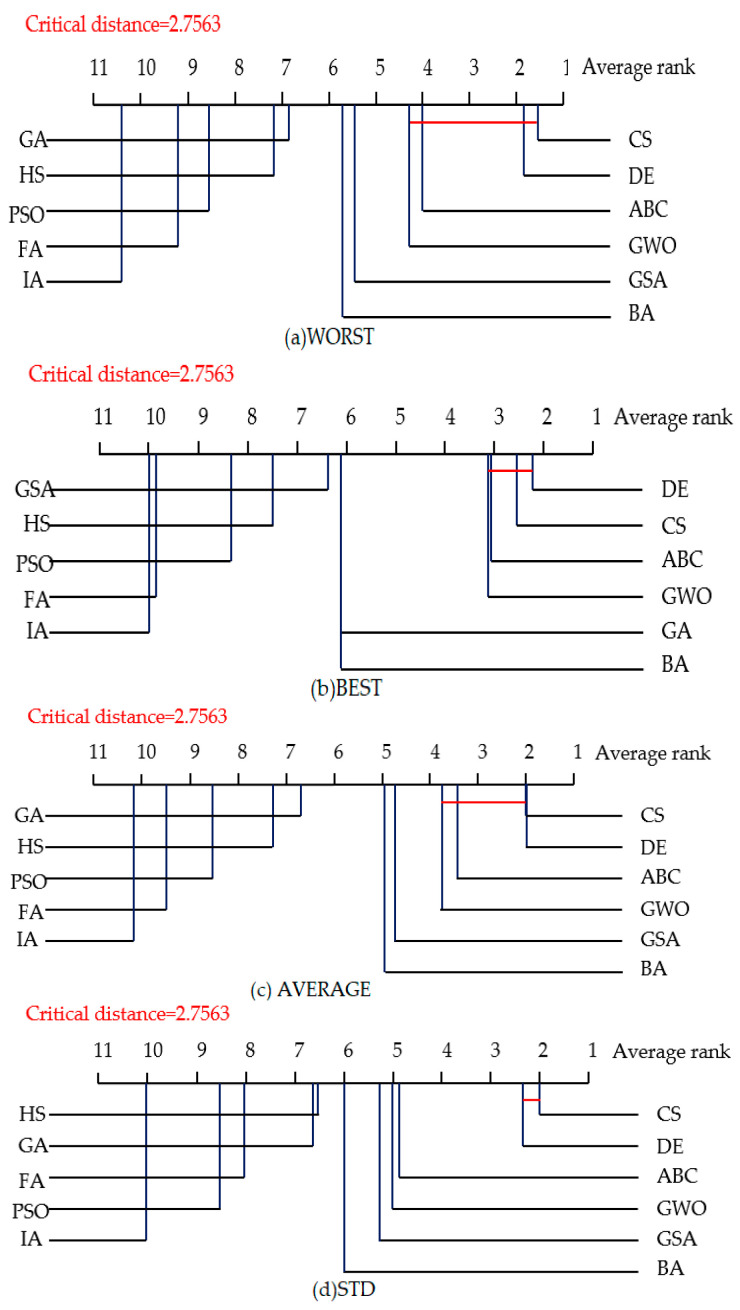
Comparison of DE (control algorithm) against other compared algorithms using the Nemenyi test for the experimental results in 10-dimensional space under parameters I.

**Figure 6:**
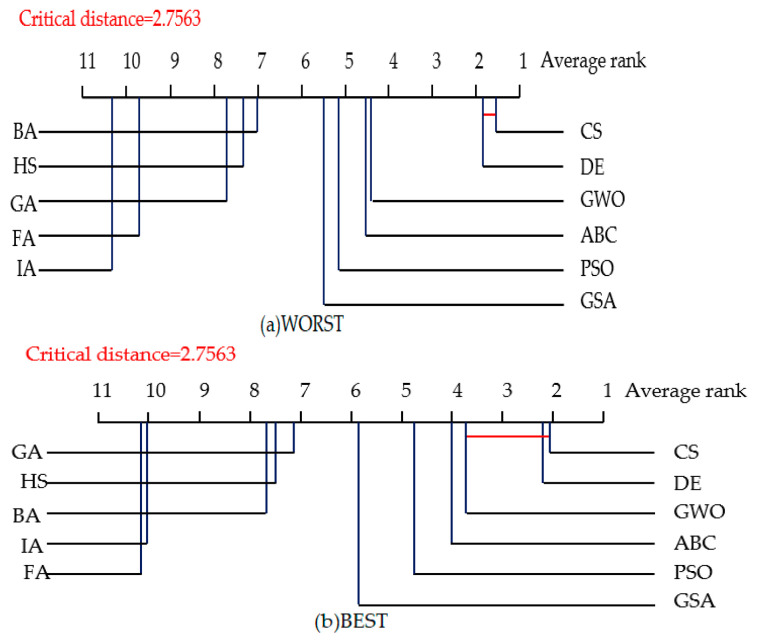

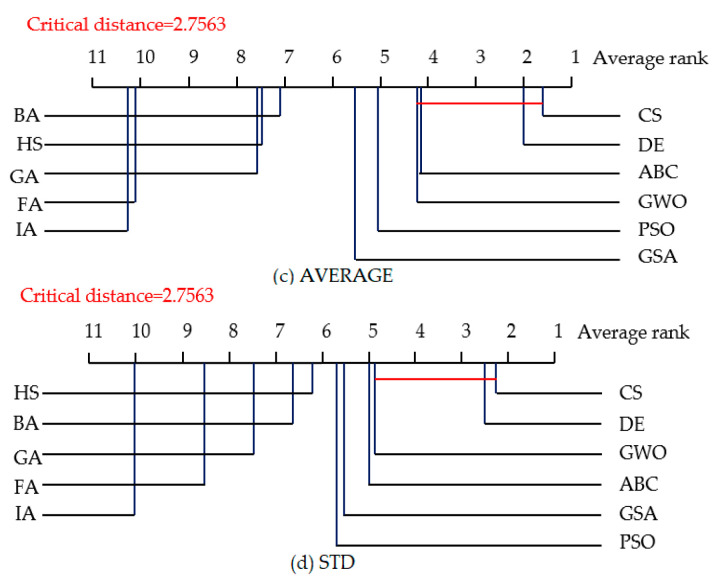
Comparison of DE (control algorithm) against other compared algorithms using the Nemenyi test for the experimental results in 10-dimensional space under parameters II.

The Nemenyi test [137] is used to test whether each of the NIOAs performed competitively against the other compared NIOAs in both the 10- and 50-dimensional spaces. In the test, two NIOAs are considered to have a significant difference in performance if their corresponding average ranks differ at least by the critical difference value given by $\mathrm{CD}=q_{\alpha }\sqrt{\frac{k\left(k+1\right)}{6N}}$. For example, $q_{\alpha }$ is equal to 3.219 at the significance level α=0.05, and thus CD takes the value of 2.7563 (*k* = 11, *N* = 30). Figure 5 and Figure 6 show the CD diagrams for each of the four evaluation criteria about the experimental results of the 10-dimensional space under the two groups of parameters. As CS obtains the best average rank on the 30 functions, CS is taken as the control algorithm. If any compared NIOA whose average rank is within one CD to that of CS, it is connected to CS with a red line, as described in Figure 5 and Figure 6. The algorithms that are unconnected to CS are considered to have a significantly different performance between them. In Figure 5a WORST, for example, the average rank for CS was 1.6333, and the critical value would be 4.3896 by adding CD. Since GSA, BA, GA, HS, PSO, FA and IA obtained 5.6333, 5.8333, 6.9, 7.2667, 8.7, 9.2 and 10.5667 for their respective average rankings, they were significantly worse compared with CS. From Figure 5 and Figure 6, we can see that CS and DE obtained the best average ranks on all four criteria, followed by ABC and GWO. CS and DE have obvious better performance than the other NIOAs. In other words, CS and DE obtained the best solutions and the best stability in low dimensional space.

**Figure 7:**
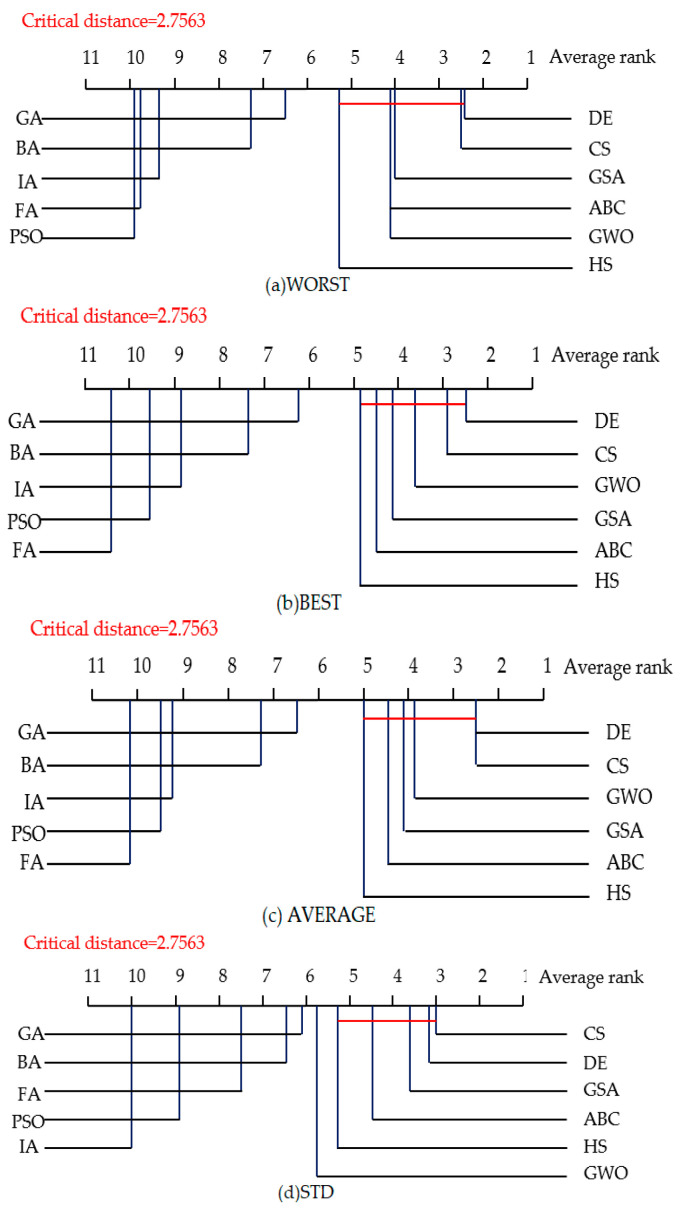
Comparison of DE (control algorithm) against other compared algorithms using the Nemenyi test for the experimental results in 50-dimensional space under parameters I.

**Figure 8:**
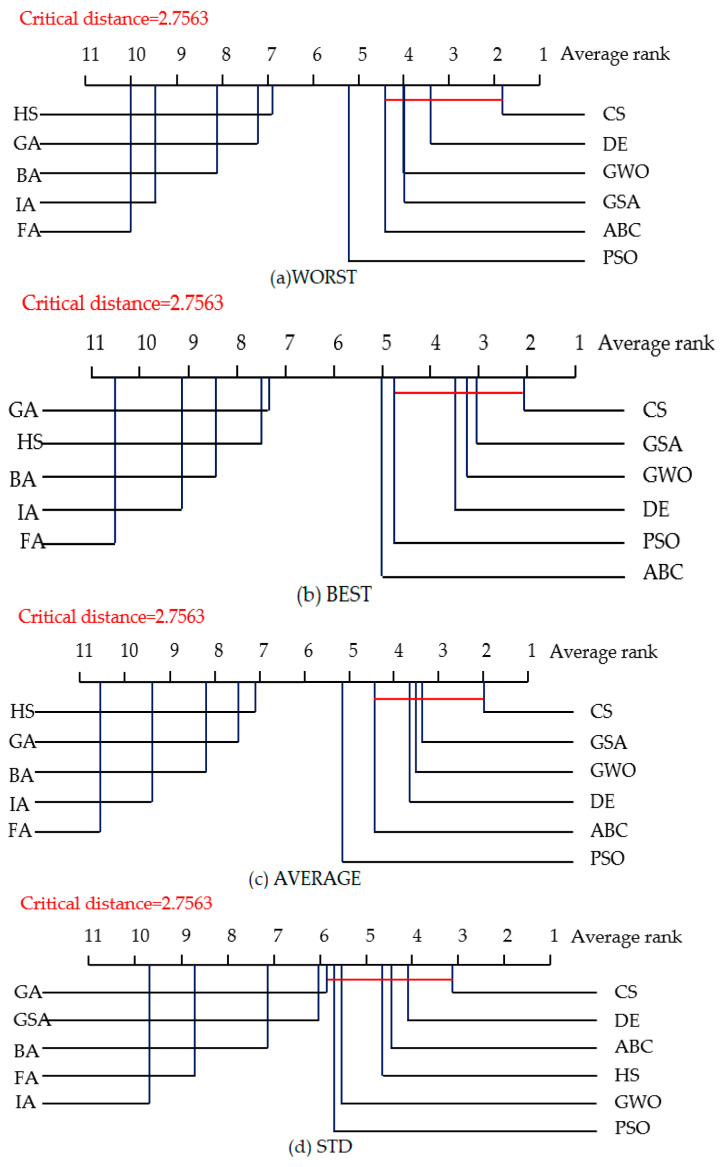
Comparison of DE (control algorithm) against other compared algorithms using the Nemenyi test for the experimental results in 50-dimensional space under parameters II.

Figure 7 shows the experimental results of the CD diagrams for the four kinds of evaluation criteria with the 50-dimensional space under the first group of parameters, and Figure 8 shows it under the second group of parameters. CS and DE still perform well on the high-dimensional space. Especially, under the first group of parameters, DE obtains the best average rank on the WORST, BEST and AVERAGE criteria, ranked the second-best average rank on the STD criterion, whereas CS ranked the first best average rank on the STD criterion. For the second group of parameters, CS ranked the first best average rank on four criteria, while DE ranked the second on the WORST and STD criteria.

### 4.4. Performance Comparison on Engineering Optimization Problem

**Figure 9:**
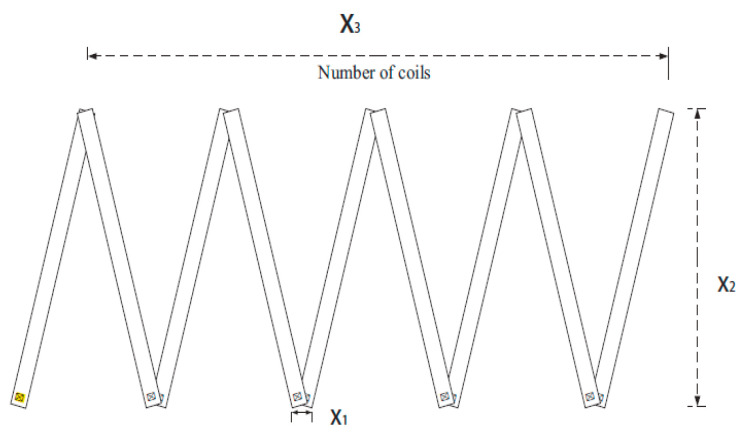
The design of tension/compression spring.

In order to further compare the performance of the 11 NIOAs, we apply them to solve the constrained engineering optimization problem, for example, Tension/Compression Spring Design. A spring is a kind of general mechanical part, which can produce a large elastic deformation under load. The weight of the spring (such as a valve spring of an internal combustion engine cylinder and spring of various buffers) has a great influence on the normal operation of the relevant mechanical equipment. The design, as shown in Figure 9, aims to minimize the weight of a tension/compression spring [138]. In this design, the constraints include the minimum deflection, shear stress, surge frequency and the limit of outer diameter.

There are three designed variables: the average coil diameter $x_{1}$, the wire diameter $x_{2}$ and the number of the active coils $x_{3}$, which together define the following complex constraints:

(46) $$\min F\left(X\right)=\left(x_{3}+2\right)x_{2}x_{1}^{2}\;s.t.\;\;g_{1}\left(X\right)=1-\frac{x_{2}^{3}x_{3}}{71,785x_{2}^{3}}\leq 0,\;g_{2}\left(X\right)=\frac{4x_{2}^{2}-x_{1}x_{2}}{12,566\left(x_{2}x_{1}^{3}-x_{1}^{4}\right)}+\frac{1}{5108x_{1}^{2}}-1\leq 0,\;g_{3}\left(X\right)=1-\frac{140.45x_{1}}{x_{2}^{2}x_{3}}\leq 0,\;g_{4}\left(X\right)=\frac{x_{1}+x_{2}}{1.5}-1\leq 0,\;\mathrm{where}\;0.05\leq x_{1}\leq 2,\;0.25\leq x_{2}\leq 1.3,\;2\leq x_{3}\leq 15.$$

Not only the objective function but also the constraint conditions should be considered in solving such constrained optimization problems. The Penalty function method is one of the most commonly used constraint processing techniques, which transforms the constrained optimization problem into an unconstrained optimization problem according to the Penalty function to the original objective function. In this study, we adopt the dynamic Penalty function [139], defined as follows:

(47) $$F\left(X\right)=f\left(X\right)+{\left(C*t\right)}^{\alpha }\overset{m}{\underset{i=1}{\sum }}G_{i}^{\beta }\left(X\right)\;G_{i}\left(X\right)=max\left(0,g_{i}\left(X\right)\right)$$

Here, t is the current iterative times, C,α,β are three parameters and in general C=1, α=1, β=2. We run each compared NIOA 20 times independently, and the iterative number of times is 1000. Table 9 gives the experimental results of the 11 compared NIOAs on the spring design problem. We can observe that all the 11 NIOAs have very close BEST values (between 0.012 and 0.013). The CS algorithm ranks first for all the four kinds of qualitative criteria: WORST, AVERAGE, BEST and STD. For the WORST criterion, CS, FA, DE, GSA and GWO achieve good results. The results of the engineering optimization problem indicate that all the 11 NIOAs obtain good results, and CS, FA, DE, GSA and GWO are better and more stable than the other six NIOAs.

## 5. Challenges and Future Directions

Indeed, how to improve the performance of NIOAs is a very complex problem, which is influenced comprehensively by the methods of parameter tuning, topology structure and learning strategy. In this study, we draw some preliminary conclusions in order to provide a referencing framework for the selection and improvement of NIOAs. In the past 30 years, a large number of meta-heuristic algorithms (more than 120 in our statistics) and their variants have been proposed in order to provide efficient and effective solutions to optimization problems in the field of AI. Although great progress has been made on the NIOAs, which have been widely and successfully applied to various application fields, challenging problems still exist, mainly reflected in the following four aspects.

1. The first one is the lack of sufficient research in fundamental theories and tools of NIOAs. From our observation, the challenges of the fundamental researches on NIOAs include the following four points.

(1) The biological or natural mechanisms imitated by the NIOAs are not yet fully clear. Most of the NIOAs are proposed by the experts of psychology or computer science and engineering, and close collaboration with biologists is extremely important in order to deeply understand and abstract such mechanisms and functions so that NIOAs can be reasonably and effectively integrated into nature, biology and the real environment.

(2) It is also necessary to lay a solid foundation of mathematical theories to support NIOAs. Such examples include a rigorous time complexity analysis and convergence proof, a deep analysis of topological structures of various NIOAs, a suitable and comprehensive theoretical explanation to balance the contradiction between easily falling into local optimum and slow convergence speed, and an in-depth analytic study of the methods of automatic parameters tuning in order to solve the parameter-dependence problem. Specifically, while working on classic fundamental works [140,141,142] with some achievements in time complexity analysis and convergence proof, the researchers give a list of future research directions, which we brief as follows: for topology analysis, it is indicated that the local neighborhood topology for some specific algorithm is more suitable for complex problems [143], and the investigation into the PSO paradigm finds that the effect of population topology interacted with the function is optimized [144]. Although these previous efforts have recommended population topologies, they still have not precisely identified the topological factors that may result in the best performance on a range of functions [144]. An automatic tuning process for parameters is usually computationally expensive, especially for real-world application problems; therefore, it is desirable to have a benchmark test that suits the NIOAs’ tuning toolbox and is easy to use [145]. Due to the lack of a solid mathematical foundation, almost all the NIOAs are working under the black-box mode; thus, there are always researchers proposing so-called “novel” algorithms and declaring that their optimizers find better solutions than other NIOAs [136].

(3) The research is not sufficient on the problem extension of basic continuous NIOAs to different optimization problems, including COPs and MOOPs. The study here on different learning strategies and topological structures of more than 120 MHAs can provide diverse solutions to COPs and MOOPs. Actually, the current research of mathematical theories in the aforementioned (2) and problem extensions mainly focus on a few NIOAs, including GA, PSO and DE, so it is required to pursue further research to more NIOAs.

(4) Another problem is the visualization platforms of NIOAs research. From our observation, there are few discussions on this aspect except for an early simple attempt [146]. In addition, few benchmark tests suit specific optimization problems such as automatic parameter tuning [145]. Owing to the insufficient and inadequate theoretical investigation on the NIOAs, it becomes quite difficult to clearly distinguish the characteristics of different NIOAs (most of the algorithms look very similar) and this, per se, becomes another optimization problem of an optimal selection of the NIOAs for a given problem. This is also a motivation that we attempt to compare and analyze 11 common NIOAs theoretically and experimentally.

2. The second one is that NIOAs are less capable of solving continuous optimization problems in complex environments. The real environments are complicated, and the optimization problems can be high-dimensional, large-scale, multi-modal and multi-objective; the optimization environments can be dynamic, highly constrained and uncertain; the fitness evaluations may contain noises, be imprecise and time-consuming, and sometimes the fitness functions can be un-deterministic. The complexity of the real environments poses a great challenge to NIOAs. Although some efforts [147,148,149] have been made to solve the aforementioned problems, how to handle these issues is still a very difficult problem.

3. The third one is too few combinations of NIOAs with other related disciplines. NIOAs intrinsically have a parallel and distributed architecture, while less attention is paid to the combinations with parallel and distributed technologies, including GPU-based hardware, robot swarm and cloud platforms. A few works [150,151,152] focus on the above issues, and interdisciplinary research is a great potential for NIOAs.

4. The fourth one is that less effort has been made to apply NIOAs to various domain problem fields. Actually, on the one hand, it is impossible to have one single NIOA to adapt to all the application problems. On the other hand, a certain kind of NIOAs may be more effective for certain kinds of problems [134]. Existing enhanced methods of NIOAs (for example, a combination of different NIOAs) lack an in-depth and targeted discussion on the reason why the enhanced methods are adopted. Furthermore, various NIOAs have been adopted to handle the same application problem, but it is not clear why this NIOA was chosen (researchers just happened to use it).

Consequently, it is our belief that in the future, researchers should tackle the following three problems in the NIOAs. These three problems indicate three future research directions for the NIOAs study.

1. Strengthening solid theoretical analysis for the NIOAs. Some theoretical problems of NIOAs are only studied in specific NIOA (for example, PSO), such as the time complexity analysis, the convergence proof, topology analysis, the automatic parameter tuning, the mechanisms of the exploitation and exploration processes. There are still many problems to be solved in the existing research work [140,141,142], and the theoretical analysis of other NIOAs needs to be analyzed deeply. COPs and MOOPs should be further studied by extending and combining the various existing NIOAs. Furthermore, it is necessary to develop a visualization platform of deconstructing, modeling and simulation of the interactions of components in NIOAs, to make it convenient to study the mechanisms of self-organization, direct/indirect communication and the processes of intelligent emergence on various swarm systems and application cases. It is also necessary to establish a benchmark test suite and easy-to-use algorithm toolbox for different problems, for example, automatic parameter tuning and the aforementioned problems in complex environments.

2. Designing novel NIOAs to solve complicated optimization problems. Many real-world optimization problems are very complex, such as the multi-model and multi-objective problems, the constrained or uncertainty problems, the large-scale optimization problems, the optimization problems with noisy, imprecise or time-varying fitness evaluations. It is another important direction to design more targeted and effective NIOAs for the above problems.

3. Deep fusion with other related disciplines. In order to improve the performance of the current NIOAs, it is indispensable to combine the NIOAs with other related disciplines or directions, such as distributed and parallel computing, machine learning, quantum computation and robot engineering. More concretely, because NIOAs by nature possess the characteristics of distributed parallelism, it is more easily and natural for them to be implemented in distributed and parallel environments, such as cloud platforms and GPU-based hardware environments. Furthermore, for some large-scale optimization problems, the robot swarm can be a good solution that combines NIOAs and robot engineering. With the support from machine learning methods, NIOAs can become efficient to handle the multi-modal multi-objective optimization problems, and on the other way around, NIOAs can provide optimization support to machine learning tasks, such as the clustering problem and the association rules mining problem.

4. Combination with specific applications. It is necessary to design customized NIOA for specific application problems; the topological structure, learning strategy and method of parameters’ selection of customized NIOAs may be suitable to a specific problem, which can acquire the good convergence speed and optimization performance. Existing applications rarely have targeted design of NIOAs; more of them use NIOAs directly or cannot explain the reason for algorithm design with specific problems.

## 6. Conclusions

Nature-Inspired Optimization Algorithms (NIOAs) can provide satisfactory solutions to the NP-hard problems, which are difficult and sometimes even impossible for traditional optimization methods to handle. Thus, the NIOAs have been widely applied to various fields both theoretically and in practice; examples including function optimization problems (convex, concave, high or low dimension and single peak or multiple peaks), combinatorial optimization problems (traveling salesman problem (TSP), knapsack problem, bin-packing problem, layout-optimization problem, graph-partitioning problem and production-scheduling problem), automatic control problems (control system optimization, robot structure and trajectory planning), image-processing problems (image recognition, restoration and edge-feature extraction), data-mining problems (feature selection, classification, association rules mining and clustering).

Many NIOAs and their variants have been proposed in the last 30 years. However, for the specific optimization problems, researchers tend to choose the NIOAs based on their narrow experiences or biased knowledge because there lacks an overall and systematic comparison and analysis study of these NIOAs. This study aims to bridge this gap; the contributions of this paper are fourfold. First, we summarize the uniform formal description for the NIOAs, analyze the similarities and differences among the 11 common NIOAs; second, we compare the performance of 11 NIOAs comprehensively, which can reflect the essential characteristics of each algorithm; third, we present a relatively comprehensive list of all the NIOAs so far, the first attempt to systematically summarize existing NIOAs, although it is very hard work; fourth, we comprehensively discuss the challenges and future directions of the whole NIOAs field, which can provide a reference for the further research of NIOAs. Actually, we are not aiming to find a super algorithm that can solve all problems in different fields once and for all (it is an impossible task). Instead, we propose a useful reference to help researchers to choose suitable algorithms more pertinently for different application scenarios in order to take a good advantage and make full use of the different NIOAs. We believe, with this survey work, that more novel-problem-oriented NIOAs will emerge in the future, and we hope that this work can be a good reference and handbook for the NIOAs innovation and applications.

Undoubtedly, it is necessary and meaningful to make a 34 comprehensive comparison of the common NIOAs, and we believe that more efforts are required to further this review in the future. First, the state-of-the-art variants of the 11 common NIOAs will be compared and analyzed comprehensively, discussing their convergence, topological structures, learning strategies, the method of parameter tuning and the application field. Second, there are more than 120 MHAs with various topological structures and learning strategies. For example, the recently proposed chicken swarm optimization (CSO) and spider monkey optimization (SMO) algorithms have a hierarchical topological structure and grouping/regrouping learning strategies. Thus, the comprehensive analysis of various topological structures and learning strategies of NIOAs is another future work.

## Figures and Tables

**Table 1:** The common symbols of NIOAs.

Conceptions	Symbols	Description
Space dimension	D, 0<d≤D	The problem space description
Population size	M, 0<i≤M	Individual quantity
Iteration times	N, 0<t≤N	Algorithm termination condition
Individual position	$x_{i}\left(t\right)=\left(x_{i,1}\left(t\right),\ldots ,x_{i,d}\left(t\right),\ldots ,x_{i,D}\left(t\right)\right)$	The expression of the *i^th^* solution on the *t^th^* iteration, also used to represent the *i^th^* individual
Local best solution	$p_{i}\left(t\right)=\left(p_{i,1}\left(t\right),\ldots ,p_{i,d}\left(t\right),\ldots ,p_{i,D}\left(t\right)\right)$	Local best solution of the *i^th^* individual on the *t^th^* iteration
Global best solution	$p_{g}\left(t\right)=\left(p_{g,1}\left(t\right),\ldots ,p_{g,d}\left(t\right),\ldots ,p_{g,D}\left(t\right)\right)$	Global best solution of the whole populationon the *t^th^* iteration
Fitness function	f(·)	Unique standard to evaluate solutions
Precision threshold	δ	Algorithm termination condition

**Table 2:** The popular variants of 11 original NIOAs.

NIOAs	Multiple Objectives	Adaptive	Spatial Property		Hybridization			
			Discrete	Continuous	Fuzzy Theory	Chaos Theory	Combination among NIOAs	Others
GA	[37]^3594^ [38]^44334^	[39]^204^	[40]^142^ [41]^73^	[42]^1405^ [43]^831^	[44]^305^	[45]^195^	[46]^420^ [47]^1373^	[43]^831^ [48]^355^
PSO	[49]^800^ [50]^67^	[51]^2363^ [52]^914^	[53]^732^ [54]^640^	[55]^296^	[52]^914^	[56]^252^	[57]^381^ [58]^11^	[59]^214^ [60]^154^ [50]^67^
ABC	[61]^334^	[62]^47^	[63]^235^ [64]^851^	[5]^3932^	[65]^42^	[66]^197^	[67]^122^	[68]^428^
BA	[69]^433^	[70]^204^	[71]^560^ [72]^285^	[73]^136^	[74]^27^	[75]^158^	[76]^64^	[73]^136^
FA	[77]^81^	[78]^66^	[79]^43^ [80]^165^	[81]^142^	[82]^45^	[83]^140^	[84]^99^	[85]^56^
IA	[86]^166^	[87]^97^	[88]^157^	[89]^141^	[90]^205^ [91]^17^	[91]^17^	[92]^166^	[93]^230^
CS	[94]^192^	[95]^114^	[96]^142^ [97]^438^	[7]^2801^	[98]^41^	[99]^104^	[100]^77^	[101]^308^
DE	[102]^350^	[103]^198^	[104]^375^ [105]^219^	[4]^1925^	[106]^251^	[107]^86^	[108]^70^ [109]^257^	[110]^81^
GSA	[111]^135^	[112]^216^	[113]^133^ [114]^114^	[12]^5909^	[115]^154^	[116]^145^	[117]^253^	[118]^152^
GWO	[119]^627^	[120]^60^	[121]^28^	[16]^6135^	[122]^225^	[123]^188^	[124]^29^	[125]^105^
HS	[126]^221^	[127]^186^	[128]^429^	[18]^6808^	[129]^38^	[130]^345^	[131]^194^	[132]^133^

**Table 3:** The time complexities of 11 compared NIOAs.

NIOAs	Time Complexity	Comments
PSO	T_upd_ = T_vec_ + T_pos_ = D∙M + D∙M = 2∙D∙M; O(T_PSO_) = O(D∙M + (3∙D∙M + M)∙N) ≈ O(D∙M∙N)	T_upd_ denotes the cost of updating velocity (T_vec_) and position (T_pos_)
GA	T_upd_ = T_cross_ + T_mut_ = D∙M + D∙M = 2∙D∙M; O(T_GA_) = O(D∙M + (2∙M∙D + M)∙N) ≈ O(D∙M∙N)	T_upd_ denotes the cost of crossover (T_cross_) and mutation (T_mut_) operations
ABC	T_upd_ = T_emp_ + T_sct_ + T_onk_ = D∙M/2 + D∙M/2 + M = D∙M + M; O(T_ABC_) =O(D∙M + (2∙M∙D + 2M)∙N) ≈ O(D∙M∙N)	T_upd_ denotes the cost of updating the positions of employed foragers (T_emp_), scouts (T_sct_) and onlookers (T_onk_)
BA	T_upd_ = T_freq_ + T_vec_ + T_pos_ = D∙M+ D∙M+ D∙M = 3∙D∙M; O(T_BA_) = O(D∙M + (4∙M∙D + M)∙N) ≈ O(D∙M∙N)	T_upd_ denotes the cost of updating the frequency (T_freq_), velocity (T_vec_) and positions (T_pos_)
IA	T_upd_ = T_den_ + T_act_ + T_cross_+ T_mut_ =M∙M + M+ D∙M + D∙M = M(M + 1) + 2 D∙M; O(T_IA_) = O(D∙M +(M∙(M +4) + 3∙M∙D)∙N) ≈ O(D∙M∙N + M∙M∙N) =O((D + M)∙M∙N)	T_upd_ is the cost of updating the density (T_den_), activity (T_act_), crossover (T_cross_) and mutation (T_mut_) operations
FA	T_upd_ = M∙M∙D; O(T_FA_) = O(D∙M +(M∙M∙D + M∙D + M)∙N) ≈ O(D∙M^2^∙N)	T_upd_ is the cost of updating the positions of fireflies
CS	T_upd_ = 2∙M∙D; O(T_CS_) = O(D∙M + (3∙M∙D + M)∙N) ≈ O(D∙M∙N)	T_upd_ is the cost of updating the host nests of cuckoos
DE	T_upd_ = M∙D+ M∙D+ M; O(T_DE_) = O(D∙M +(4M∙D + 2∙M)∙N) ≈ O(D∙M∙N)	T_upd_ is the cost of crossover mutation and selection operations
GSA	T_upd_ = T_grav_ + T_vec_ + T_pos_ = M∙M+ M∙D+ M∙D; O(T_GSA_) = O(D∙M + (M∙M + 3∙M∙D + M)∙N) ≈ O(D∙M∙N + M∙M∙N) = O((D + M)∙M∙N)	T_upd_ is the cost of updating gravitational acceleration (T_grav_), velocity (T_vec_) and position (T_pos_)
GWO	T_upd_ = M∙D; O(T_GWO_) = O(D∙M + (2M∙D + M)∙N) ≈ O(D∙M∙N)	T_upd_ denotes the cost of updating the positions of wolves
HS	T_upd_ = M∙D; O(T_HS_) = O(D∙M + (2M∙D + M)∙N) ≈ O(D∙M∙N)	T_upd_ is the cost of updating harmony vectors

**Table 4:** The parameters of the 11 NIOAs.

Algorithms	Parameters I	Parameters II
GA	$\vartheta_{1}$ = 1, $\vartheta_{2}\;$= 0.8, $\vartheta_{3}$ = 0.2	$\vartheta_{1}\;$= 1, $\vartheta_{2}$ = 0.75, $\vartheta_{3}\;$= 0.25
PSO	$c_{1}\;$= 2, $c_{2\;}$= 2	$c_{1}\;$= 1.5, $c_{2}\;$= 1.5
ABC	F=M/2*, Limit =* 20	F=M/3, *Limit* = 30
BA	α = 0.9, γ = 0.9 $A_{max}$ = 100, $A_{min}$ = 1, $f_{max}$ = 100, $f_{min}$ = 1	α = 0.8, γ = 0.8 $A_{max}$ = 150, $A_{min}$ = 1, $f_{max}$ = 150,$\;f_{min}$ = 1
IA	$P_{c}$ = 0.8, $P_{m}$ = 0.2	$P_{c}$ = 0.75, $P_{m}$ = 0.25
FA	γ = 0.6, *step* = 0.4, $\beta_{0}$ = 1	γ = 0.5, *step* = 0.5, $\beta_{0}$ = 1.1
CS	α = 1, $P_{a}$ = 0.25	α = 1.1, $P_{a}$ = 0.15
DE	*F* = 0.5, *CR* = 0.1	*F* = 0.6, *CR* = 0.2
GSA	$G_{0}$ = 100, α = 20	$G_{0}$ = 90, α = 15
GWO	None	None
HS	*HMCR* = 0.995, *PAR* = 0.4, *BW* = 1	*HMCR* = 0.85, *PAR* = 0.5, *BW* = 0.9

**Table 5:** The number of wins and corresponding functions of each criterion for 11 NIOAs on parameters I.

	Criteria	WORST	AVERGAE	BEST	STD
NIOAs					
DE	D = 10	**10** (F5, F6, F8, F9, F10, F17, F20, F27, F29, F30)	**10** (F5, F6, F8, F9, F10, F17, F19, F20, F27, F30)	**13** (F6, F9, F11, F14, F15, F16, F17, F18, F19, F20, F21, F27, F30)	**7** (F5, F6, F8, F9, F23, F28, F30)
	D = 50	**9** (F4, F6, F8, F16, F20, F25, F27, F28, F30)	**8** (F6, F9, F11, F20, F25, F27, F29, F30)	**8** (F6, F9, F11, F20, F22, F25, F27, F30)	**7** (F4, F6, F21, F25, F27, F28, F30)
CS	D = 10	**17** (F2, F3, F4, F11, F12, F13, F14, F15, F16,F18, F19, F21, F23, F24, F25, F26, F28)	**16** (F2, F3, F4, F11, F12, F13, F14, F15, F16, F18, F21, F22, F24, F25, F26, F28)	**7** (F2, F3, F4, F12, F13, F25, F26)	**17** (F2, F3, F4, F10, F11, F12, F13, F14, F15, F16, F17, F18, F19, F20, F21, F27, F29)
	D = 50	**8** (F3, F12, F14, F15, F18, F19, F24, F29)	**10** (F1, F3, F4, F12, F13, F14, F15, F18, F19, F28)	**8** (F3, F4, F12, F13, F14, F18, F19, F28)	**9** (F3, F10, F14, F15, F16, F18, F19, F22, F29)
HS	D = 10	-	-	-	-
	D = 50	**2** (F9, F23)	**2** (F8, F23)	**5** (F5, F8, F21, F23, F29)	**3** (F9, F23, F24)
GSA	D = 10	**4** (F1, F7, F9, F22)	**3** (F1, F7, F9)	**3** (F1, F7, F9)	**5** (F1, F7, F9, F22, F25)
	D = 50	**6** (F1, F2, F7, F11, F13, F26)	**3** (F2, F7, F26)	**4** (F1, F2, F7, F26)	**9** (F1, F2, F5, F7, F8, F11, F12, F13, F26)
GWO	D = 10	-	**1** (F29)	**3** (F5, F8, F22)	**2** (F25, F26)
	D = 50	**3** (F5, F17, F21)	**7** (F5, F10, F16, F17, F21, F22, F24)	**3** (F16, F17, F24)	-
ABC	D = 10	-	**1** (F23)	**5** (F10, F23, F24, F28, F29)	-
	D = 50	**2** (F10, F22)	-	-	**1** (F17)
PSO	D = 10	-	-	**1** (F6)	-
	D = 50	-	-	**2** (F10, F15)	-
FA	D = 10	-	-	-	-
	D = 50	-	-	-	**1** (F20)
BA		-	-	-	-
GA		-	-	-	-
IA		-	-	-	-

**Table 6:** The number of wins and corresponding functions of each criterion for 11 NIOAs on parameters II.

	Criteria	WORST	AVERGAE	BEST	STD
NIOAs					
DE	D = 10	**11** (F5, F6, F8, F10, F14, F15, F17, F18, F19, F20, F30)	**12** (F5, F6, F8, F15, F17, F18, F19, F20, F23, F27, F29, F30)	**14** (F5, F6, F8, F9, F14, F15, F16, F17, F18, F19, F20, F23, F27, F30)	**9** (F5, F6, F8, F15, F18, F19, F25, F28, F30)
	D = 50	**7** (F4, F6, F20, F25, F26, F27, F28)	**4** (F6, F9,F25, F27)	**5** (F6, F9, F25, F27, F30)	**5** (F4, F6, F25, F27, F28)
CS	D = 10	**16** (F1, F2, F3, F4, F11, F12, F13, F16, F21, F23, F24, F25, F26, F28, F29)	**14** (F2, F3, F4, F11, F12, F13, F14,F16, F21, F22, F24, F25, F26, F28)	**11** (F2, F3, F4, F11, F12, F13, F21, F22, F25, F26, F28)	**15** (F1, F2, F3, F4, F11, F12, F13, F14, F16, F17, F20, F21, F23, F27, F29)
	D = 50	**12** (F1, F2, F11, F12, F13, F14, F15, F18, F19, F24, F29, F30)	**13** (F1, F2, F4, F11, F12, F13, F14, F15, F18, F19, F28, F29, F30)	**11** (F2, F4, F11, F12, F13, F14,F15, F18, F19, F28, F29)	**12** (F1, F2, F11, F12, F13, F14, F15, F17, F18, F19, F29, F30)
HS	D = 10	-	-	-	**1** (F24)
	D = 50	-	-	-	**7** (F5, F8, F16, F21, F23, F24, F26)
GSA	D = 10	**3** (F7, F9, F22)	**3** (F1, F7, F9)	**3** (F1, F7, F9)	**3** (F7, F9, F22)
	D = 50	**2** (F7, F10)	**3** (F7, F10, F26)	**4** (F1, F7, F10, F26)	-
GWO	D = 10	-	**1** (F10)	**2** (F10, F29)	-
	D = 50	**7** (F5, F8, F16, F17, F21, F22, F23)	**9** (F5, F8, F16, F17, F20, F21, F22, F23, F24)	**8** (F5, F8, F16, F17, F20, F21, F23, F24)	-
ABC	D = 10	-	-	-	**1** (F26)
	D = 50	-	-	**1** (F22)	-
PSO	D = 10	-	-	**1** (F24)	-
	D = 50	**1** (F3)	**1** (F3)	**1** (F3)	**3** (F3, F7)
FA	D = 10	-	-	-	**1** (F10)
	D = 50	-	-	-	**2** (F20, F22)
GA	D = 10	-	-	-	-
	D = 50	**1** (F9)	-	-	**2** (F9, F10)
BA		-	-	-	-
IA		-	-	-	-

**Table 7:** The sensitivity comparison of each criterion for 11 NIOAs under two groups of parameters.

	Criteria	WORST	AVERGAE	BEST	STD
NIOAs					
DE	D = 10	-	-	-	-
	D = 50	**7** (F1 **, F2, F9, F12, F13 ***, F15 **, F30 **)	**8** (F1 **, F2, F7, F10, F12, F13 **, F15, F30)	**7** (F2, F3, F7, F8, F10, F18, F22)	**11** (F1 **, F2, F3, F4, F9, F12, F13 **, F15, F18, F22, F30 **)
CS	D = 10	-	-	-	-
	D = 50	**2** (F1, F18)	**1** (F18)	**2** (F8, F12)	**2** (F14, F29)
HS	D = 10	-	-	-	-
	D = 50	**8** (F1 ***, F2, F4, F5, F9, F12 **, F13, F30)	**8** (F1 ***, F2 **, F4, F8, F12 **, F13, F19, F30)	**8** (F1 **, F2, F4, F8, F12 **, F13, F19, F30)	**10** (F1 ***, F2, F4, F9, F12 **, F13, F15, F18, F25, F30)
GSA	D = 10	-	-	-	-
	D = 50	**6** (F1, F2, F9, F12 **, F13, F14 **)	**3** (F12 ***, F14, F22)	**4** (F8, F14, F19, F22)	**7** (F2 **, F9, F12 ***, F13, F14 **, F18, F19)
GWO	D = 10	-	-	-	-
	D = 50	**4** (F1, F9, F18, F19)	**3** (F4, F7, F13)	**3** (F1, F2, F19)	**3** (F13, F19, F24)
ABC	D = 10	**2** (F18, F30)	**2** (F18, F30)	**2** (F18, F30)	**2** (F18, F30)
	D = 50	**6** (F1, F11, F12, F13, F18, F19)	**2** (F1, F15)	**1** (F19)	**4** (F12, F13, F14, F19)
PSO	D = 10	**12** (F1 **, F2, F3, F7, F9, F12 **, F13, F14, F15, F18, F19, F30)	**11** (F1 **, F2, F3, F9, F12, F13, F14, F15, F18, F19, F30)	**8** (F1 **, F3, F9, F12, F13, F14, F18, F30)	**12** (F1 ***, F2 **, F3 **, F7, F9, F12, F13, F14, F15 **, F18, F19, F30)
	D = 50	**12** (F1 **, F2, F3 **, F4 **, F11, F12, F13 **, F14 **, F15 **, F18 **, F19 **, F30)	**14** (F1 **, F2 **, F3 **, F4 **, F11, F12 **, F13 **, F14 ***, F15 **, F18, F19 **, F26, F28, F30 **)	**15** (F1 ***, F2 **, F3 **, F4 **, F9, F11, F12 **, F13 **, F14 **, F15 **, F18 **, F19 **, F26, F28, F30)	**12** (F1 **, F2 **, F3 **, F4 **, F11 ***, F12, F13 **, F14 ***, F15 ***, F18 **, F19 ***, F30 **)
FA	D = 10	-	-	-	-
	D = 50	**4** (F2, F14, F17, F29)	**4** (F12, F14, F18, F29)	**4** (F12, F14, F15, F17)	**4** (F1, F13, F14, F17)
BA	D = 10	-	-	-	-
	D = 50	**3** (F2, F11, F19)	**6** (F2, F3, F12, F14, F15, F26)	**2** (F3, F9)	**6** (F2, F4, F11, F13, F19, F28)
GA	D = 10	**3** (F1, F18, F19)	**1** (F1)	**1** (F1)	**2** (F1, F18)
	D = 50	**4** (F15, F19, F26, F30)	**6** (F1, F14, F15, F18, F19, F26)	**5** (F1 **, F13, F15, F18, F26)	**4** (F1, F11, F15, F30)
IA	D = 10	-	-	-	-
	D = 50	-	**1** (F14)	-	**2** (F1, F13)

**Table 8:** Summary of the Friedman Statistics $F_{F}$ (k=11,N=30) and the critical value in terms of each evaluation criteria (*k*: #comparing algorithms; *N*: #data sets).

Dimensions	NIOAs Parameters	Criteria	$F_{F}$	Critical Value (α=0.05)
10-dimensional space	Parameters I	WORST	89.9707	1.8634
		BEST	79.0949	
		AVERAGE	94.9530	
		STD	34.6416	
	Parameters II	WORST	80.1552	
		BEST	78.3713	
		AVERAGE	95.4905	
		STD	27.9553	
50-dimensional space	Parameters I	WORST	68.9997	
		BEST	69.7277	
		AVERAGE	71.4619	
		STD	32.7366	
	Parameters II	WORST	61.3683	
		BEST	92.1188	
		AVERAGE	75.6435	
		STD	14.9259	

**Table 9:** Experimental results of 11 NIOAs on spring design problem.

Algorithm	WORST	AVERAGE	BEST	STD
GA	0.029080	0.016709	0.012691	0.004163
PSO	0.030457	0.015028	0.012746	0.005420
ABC	0.016446	0.014202	0.012827	0.001062
BA	0.044217	0.023193	0.013194	0.011202
IA	0.031477	0.021735	0.013134	0.006702
FA	0.012880	0.012733	0.012718	3.48 × 10^−5^
CS	**0.012670**	**0.012666**	**0.012665**	**1.27** × **10^−6^**
DE	0.013397	0.013007	0.012755	0.000201
GSA	0.013073	0.012953	0.012740	9.06 × 10^−5^
GWO	0.012821	0.012715	0.012672	3.00 × 10^−5^
HS	0.032620	0.020375	0.012877	0.006328

## Data Availability

All the data are generated by 30 BBOB functions and 11 NIOAs, the results are included within the manuscript and supplementary materials.

## Supplementary Materials

The supplementary figures and tables are available online at https://www.mdpi.com/article/10.3390/e23070874/s1.
