# Supplementary material for: A Comparative Study of Common Nature-Inspired Algorithms for Continuous Function Optimization
Source: Entropy (Basel). 2021 Jul 8;23(7):874. doi: 10.3390/e23070874 (PMC8304592; doi:10.3390/e23070874)
Supplement: Supplementary file 1 [file entropy-23-00874-s001.zip › entropy-1223717-Supplementary Materials.pdf]

Supplementary Material A

Table S1. The summary of MHAs

No.	Algorithm Name	Authors	Published year	Total number (average number per year) of published papers
1	Genetic Algorithm	John Holland	1975	94381 (2051)
2	Particle Swarm Optimization Algorithm	James Kennedy, et al.	1995	35021(1346)
3	Differential Evolution Algorithm	Rainer Storn, et al.	1997	13801(575)
4	Ant Colony Optimization Algorithm	Marco Dorigo, et al.	1992	9137(315)
5	Artificial Bee Colony Algorithm	Dervis Karaboga	2005	5067(316)
6	Immune Algorithm	Mori Kazuyuki, et al.	1991	4771(159)
7	Firefly Algorithm	K.N. Krishnanand, et al.	2008	2505(192)
8	Cuckoo Search Algorithm	Xin-She Yang, et al.	2009	2470(205)
9	Harmony Search Algorithm	Zong Woo Geem, et al.	2001	2001(100)
10	Bat Algorithm	Xin-She Yang	2012	1725(191)
11	Gravitational Search Algorithm	Esmat Rashedi, et al.	2009	1445(120)
12	Grey Wolf Optimization	Seyedali Mirjalili, et al.	2014	1105(157)
13	Biogeography-Based Optimization Algorithm	Dan Simon	2008	950(73)
14	The Bees Algorithms	D.T. Pham, et al.	2006	865(57)
15	Imperialist Competitive Algorithm	Esmaeil Atashpaz-Gargari, et al.	2007	821(58)
16	Shuffled Frog Leaping Algorithm	Muzaffar Eusuff, et al.	2003	762(42)
17	Artificial Fish Swarm Algorithm	Dervis Karaboga	1998	750(32)
18	Bacterial Foraging Optimization	Kevin M. Passino	2002	739(38)
19	Whale Optimization Algorithm	Seyedali Mirjalili, et al.	2016	658(131)
20	Flower Pollination Algorithm	Xin-She Yang	2012	621(69)
21	Fruit Fly Optimization Algorithm	Wen-Tsao Pan	2012	583(64)
22	Firework Algorithm	Ying Tan, et al.	2009	453(37)
23	BeeHive Algorithm	Horst F. Wedde, et al.	2004	422(24)
24	Differential Search Algorithm	Pinar Civicioglu	2012	396(44)
25	Brain Storm Optimization	Yuhui Shi	2013	368(46)
26	Krill Herd Algorithm	Amir H Gandomi, et al.	2012	290(32)
27	Big Bang–Big Crunch Algorithm	Osman K. Erol, et al.	2005	275(17)
28	Invasive Weed Optimization Algorithm	A. R. Mehrabian, et al.	2006	229(15)
29	Water Cycle Algorithm	Hadi Eskandar, et al.	2012	218(24)
30	Symbiotic Organisms Search Algorithm	Cheng Min Yuan, et al.	2014	205(29)
31	Crow Search Algorithm	Alireza Askarzadeh	2016	202(40)
32	Lion Algorithm	Maziar Yazdani, et al.	2015	197(32)
33	Search Group Algorithm	Matheus Silva GoncAlves, et al.	2015	191(31)
34	Intelligent Water Drops Algorithm	Hamed Shah_Hossinie	2007	187(13)
35	Grasshopper Optimization Algorithm	Seyedeh Zahra Mirjalili1, et al.	2018	180(60)
36	Moth-Flame Optimization Algorithm	Seyedali Mirjalili	2015	177(29)
37	Black Hole Algorithm	Abdolreza Hatamlou, et al.	2013	175(21)
38	Charged System Search Algorithm	A. Kaveh, et al.	2010	164(14)
39	Glowworm Swarm Optimization Algorithm	K.N. Krishnanand, et al.	2005	164(10)
40	Monkey Algorithm	Ye Zhen cheng, et al.	2007	162(11)
41	Geometric Partitioning Algorithm	Wang Tao, et al.	2012	159(7)
42	Backtracking Search Optimization Algorithm	Pinar Civicioglu	2013	139(17)
43	Chicken Swarm Optimization Algorithm	Xianbing Meng, et al.	2014	135(19)
44	Hunting Search Algorithm	R. Oftadeh, et al.	2010	131(11)

45	Chemical Reaction Optimization Algorithm	Nazmul Siddique, et al.	2017	124(31)
46	Cat Swarm Optimization Algorithm	Shu Chuan Chu, et al.	2006	118(7)
47	Bumblebees Algorithm	Francesc Comellas, et al.	2003	98(8)
48	Migrating Birds Optimization Algorithm	Ekrem Duman, et al.	2012	95(10)
49	Stochastic Fractal Search Algorithm	Hamid Salimi	2015	92(15)
50	Central Force Optimization Algorithm	R. A. Formato, et al.	2007	90(6)
51	Virtual Ant Algorithm	Xin-She Yang, et al.	2006	81(5)
52	Wolf Search Algorithm	Rui Tang, Simon Fong, et al.	2013	77(8)
53	Colliding Bodies Optimization Algorithm	A. Kaveh, et al.	2014	74(10)
54	Lightning Search Algorithm	Hussain Shareef, et al.	1999	65(10)
55	Artificial Plant Optimization Algorithm	Liu Kun	2013	65(8)
56	Bird Swarm Algorithm	Xian-Bing Meng, et al.	2016	59(11)
57	Pigeon-Inspired Optimization Algorithm	Haibin Duan, et al.	2014	59(8)
58	Sheep Optimization Algorithm	Robert Sawko, et al.	2008	52(4)
59	Shark Algorithm	Michael Hersovici, et al.	1998	51(2)
60	Virus Optimization Algorithm	Yun-Chia Liang, et al.	2016	50(10)
61	Self-Driven Particles Algorithm	Tamas Vicsek, et al.	1995	49(1)
62	Japanese Tree Frogs Algorithm	Hugo Hernández	2012	48(5)
63	League Championship Algorithm	Ali Husseinzadeh Kashan	2009	48(4)
64	Group Search Optimizer Algorithm	He S, et al.	2006	47(3)
65	Vortex Search Algorithm	Berat Dogˇan, et al.	2015	45(7)
66	Spider Monkey Optimization Algorithm	Jagdish Chand Bansal, et al.	2014	42(6)
67	Multi-Verse Optimizer Algorithm	Hatamlou, Abdolreza, Mirjalili	2016	35(7)
68	River Formation Dynamics Algorithm	Pablo Rabanal	2007	33(2)
69	Gradient Evolution Algorithm	R.J. Kuo, et al.	2015	31(5)
70	Social Emotional Optimization Algorithm	Subhransu Sekhar Dash	2010	30(2)
71	Marriage in Honey-bees Optimization Algorithm	Hussein A. Abbass	2001	30(1)
72	Wasp Algorithm	Guy Thbr Aulal,et al.	1991	29(0)
73	Photosynthetic Algorithm	Haruhiko Murase	2000	29(1)
74	Method of Musical Composition Algorithm	Roman Anselmo Mora-Gutiérrez	2014	28(4)
75	Golden ball Algorithm	E. Osaba	2014	27(3)
76	Electro-Magnetism Optimization Algorithm	Erik Cuevas, et al.	2011	27(2)
77	Stochastic Searching Networks Algorithm	John.Mark. Bishop	1989	27(0)
78	The Ant Lion Optimizer Algorithm	Liping Xie	2015	21(3)
79	Car Tracking Optimization Algorithm	Jian Chen	2018	19(6)
80	Hierarchical Swarm Model Algorithm	Hanning Chen, et al.	2010	19(1)
81	Galaxy-based Search Algorithm	Hamed Shah-Hosseini	2011	18(1)
82	Paddy Field Algorithm	Upeka Premaratne, et al.	2009	18(1)
83	Dolphin Echolocation Algorithm	A. Kaveh, et al.	2013	17(2)
84	Human Mental Search Algorithm	Seyed Jalaleddin Mousavirad	2017	16(4)
85	Biology Migration Algorithm	Qingyang Zhang	2019	15(7)
86	Artificial Cooperative Search Algorithm	Pinar Civicioglu	2013	15(1)
87	Queen-bee Evolution Algorithm	Sung Hoon Jung	2003	15(0)
88	Eagles Strategy Algorithm	Xin-She Yang	2010	14(1)
89	Wisdom of Artificial Crowds Algorithm	Roman Yampolskiy	2012	14(1)
90	Group Area Search Algorithm	Liu Changjun, et al.	2013	13(1)
91	Bean Optimization Algorithm	Xiaoming Zhang, et al.	2010	12(1)
92	Consultant-Guided Search Algorithm	Serban Iordache	2010	12(1)
93	Natural Aggregation Algorithm	Fengji Luo	2016	11(2)
94	Human-Inspired Algorithms	Luna Mingyi Zhang,et al.	2009	11(0)
95	Cooperative Bees Swarm Algorithm	Habiba Drias, et al.	2005	11(0)
96	Ecology-Inspired Optimization Algorithm	Rafael Stubs Parpinelli, et al.	2011	10(1)

97	Virus Colony Search Algorithm	Mu Dong Li	2016	9(1)
98	Anarchic Society Optimization Algorithm	Hossein Shayeghi, et al.	2012	9(1)
99	Roach Infestation Optimization Algorithm	Timothy C. Havens	2008	9(0)
100	Emperor Penguins Colony Algorithm	Sasan Harifi	2019	8(4)
101	Amoeba Based Algorithm	Yajuan Zhang, et al.	2012	8(0)
102	Egyptian Vulture Optimization Algorithm	Chiranjib Sur, et al.	2013	7(0)
103	Synergistic Fibroblast Optimization Algorithm	TT DHIVYAPRABHA	2018	6(2)
104	Termite Colony Optimization Algorithm	Ramin Hedayatzadeh, et al.	2010	6(0)
105	Slime Mould Optimization Algorithm	David. Monismith, et al.	2008	6(0)
106	Doves Algorithm	Mu-ChunSua, et al.	2009	5(0)
107	Virtual Bee Algorithm	Xin-She Yang, et al.	2005	5(0)
108	Hydrozoan Algorithm	Daranat Tansui, et al.	2017	4(1)
109	Football Game Algorithm	Elyas Fadakar, et al.	2016	4(0)
110	Weightless Swarm Algorithm	Ting, et al.	2012	4(0)
111	The Great Salmon Run Algorithm	Ahmad Mozaffari, et al.	2012	4(0)
112	Fast Bacterial Swarming Algorithm	Ying Chu, et al.	2008	4(0)
113	Dynamic Partition Search Algorithm	Gaoji Sun, et al.	2014	3(0)
114	Bat Sonar Optimization Algorithm	Dr. Mohammed, et al.	2012	3(0)
115	Spiral Dynamics Inspired Optimization Algorithm	Kenichi Tamura, et al.	2010	3(0)
116	Monarchy Metaheuristic Algorithm	Ibtissam Ahmia	2019	2(1)
117	Pity Beetle Algorithm	Nikos Ath. Kallioras, et al.	2018	2(0)
118	Eel Swarm Intelligence Algorithm	Yaosheng Sun, et al.	2014	2(0)
119	The OptBees Algorithm	Renato Dourado Maia, et al.	2012	2(0)
120	Good Lattice Swarm Algorithm	Shoubao Su, et al.	2007	2(0)
121	Artificial Physics Optimization Algorithm	Liping Xie, et al.	2009	1(0)
122	Benchmarking-based Optimization Algorithm	Seyedali Mirjalili, et al.	2014	0(0)
123	Spiral Milipede-Inspired Routing Algorithm	Olufemi Adeluyi, et al.	2012	0(0)
124	Self-Organizing Migrating Algorithm	Haruhiko Murase	2004	0(0)

Supplementary Material B

Table S2 Experiments on GA, PSO, ABC, BA, IA, FA for F1~F10 functions under D=10 on parameters I

Problems	Criteria	GA	PSO	ABC	BA	IA	FA
F1	WORST	1150905.31	7389317157.72	13090309.85	1027533420.23	19522409138.11	24567892868
	AVERAGE	425280.20	4061797069.88	2354181.14	676040724.22	9126518156.35	15757912820
	BEST	86762.9280	2263632443.69	250424.37	396310175.71	3662679222.10	6164854714
	STD	358292.34	1305011625.0	2794239.66	189138604.50	4604858548.44	5628364779
F2	WORST	510.5488	2069.9649	329.6954	498.4525	12210.3822	14044.2535
	AVERAGE	340.0222	1330.5235	227.9535	404.9380	5102.5600	7390.9887
	BEST	236.9765	595.0111	201.8839	285.7611	1746.3919	2689.8312
	STD	72.4484	425.1034	29.3396	60.6307	2848.3579	2930.1923
F3	WORST	53174.2753	51188.3754	13552.5320	4457.3288	77441.3250	25927.7976
	AVERAGE	25524.9354	28271.2175	8625.3882	2553.8278	27603.4126	17336.2779
	BEST	7045.4792	10776.4717	3866.8109	1393.6205	8500.3786	9795.1880
	STD	13834.8458	11253.0904	2719.8883	731.8650	18631.4457	4133.6863
F4	WORST	483.0629	785.2114	408.5754	450.1486	1999.0264	3100.8899
	AVERAGE	429.4396	657.9673	405.9633	432.9008	1231.3359	1980.1695
	BEST	408.8489	522.2490	400.8356	418.3189	610.8229	682.4590
	STD	26.4812	76.3839	2.0759	8.6482	472.0341	712.7876
F5	WORST	560.9378	587.9662	526.5166	544.7179	647.3552	634.2078
	AVERAGE	549.7743	576.2792	516.1518	537.1692	592.5278	613.7647
	BEST	540.1125	566.5143	509.1870	528.6138	553.0772	594.6531
	STD	6.6747	5.6608	4.9900	4.5430	23.1560	11.8276
F6	WORST	634.4364	663.2841	601.9021	633.0003	684.6893	668.3584
	AVERAGE	627.9288	647.3828	600.9950	619.5374	654.0561	657.6453
	BEST	614.8519	631.5171	600.5439	609.0952	631.5448	646.7685
	STD	4.8505	7.0669	0.3112	5.5501	14.8853	5.0797
F7	WORST	842.9314	1064.1771	747.3148	807.5003	862.9237	1143.9438
	AVERAGE	788.6708	956.5695	733.9866	792.5893	847.3867	1073.7316
	BEST	763.6542	888.5229	719.9530	769.4571	816.7369	924.9750
	STD	21.3411	45.6912	6.4024	12.2652	17.7236	46.8772
F8	WORST	883.5876	911.1776	826.1870	848.7434	915.3350	911.2001
	AVERAGE	859.2423	892.5746	816.1858	838.6272	882.3561	889.0791
	BEST	841.2022	867.8461	805.8347	817.5070	854.7085	865.6985
	STD	11.4949	11.7009	5.3027	8.0652	15.9224	10.3804
F9	WORST	1415.5520	3718.2530	993.5717	1155.4899	3690.5738	2655.3771
	AVERAGE	995.2913	2519.1250	921.8393	1093.6353	2104.1593	2149.4344
	BEST	905.9393	1630.3400	901.0264	990.0752	1120.4522	1846.1460
	STD	138.5490	612.0259	22.3470	40.8872	731.5220	194.3117
F10	WORST	2155.7415	2734.0386	1845.1448	2831.3932	3674.0820	2564.8308

	AVERAGE	1732.3176	2235.8698	1512.4645	2314.1281	3129.6479	2416.1235
	BEST	1216.8176	1762.1202	**1068.9142**	1880.2042	2568.2145	2150.4408
	STD	262.0993	281.4933	159.2519	209.5393	320.4662	93.4708

Table S3. Experiments on GA, PSO, ABC, BA, IA, FA for F11~F20 functions under D=10 on parameters I

**Problems**	**Criteria**	**GA**	**PSO**	**ABC**	**BA**	**IA**	**FA**
F11	WORST	3353.3490	2997.1209	1194.2442	1390.6776	21983.6778	3996.4441
	AVERAGE	1677.2785	1948.4840	1132.5766	1232.4689	9105.3149	2263.2036
	BEST	1136.2457	1411.8020	1109.5843	1147.2468	1793.8031	1568.1480
	STD	548.1344	484.4845	22.0899	51.8267	6693.3168	617.2291
F12	WORST	6282328.45	220621427.43	2941151.62	22326028.35	1504967593.27	2462273071.75
	AVERAGE	1265601.47	49805930.83	758542.9017	11784892.18	624969107.63	1232646467.20
	BEST	28827.8882	5967577.07	42784.9439	1172436.3388	90899112.6105	201224957.66
	STD	1559534.71	47998812.51	708355.8747	6320601.7430	395348418.65	633009084.78
F13	WORST	32435.1995	615234.3909	11929.5318	142455.3071	274626626.33	80247034.8915
	AVERAGE	13860.7592	186693.3386	5265.9325	51456.3270	75763924.2218	17790281.9103
	BEST	1536.7336	5059.5831	1646.4193	13980.4509	11662305.2984	17566.3000
	STD	10334.5952	168118.5267	3517.0810	37856.7449	73781855.1350	27397284.9648
F14	WORST	27221.1091	28900.8339	2101.9929	1688.8978	12519030.0478	1655.1740
	AVERAGE	7299.8736	4862.3112	1632.3267	1580.9151	1944836.3496	1512.3914
	BEST	1501.9967	1587.1991	1448.8388	1497.4601	18368.5321	1449.4438
	STD	7707.0313	8247.2709	158.0399	56.8970	3450011.6671	42.6433
F15	WORST	30619.5770	105730.0253	4138.2424	3212.4854	58424093.5581	3918.1309
	AVERAGE	10932.5250	24796.3325	2076.6279	2160.9289	5603756.1876	2732.1827
	BEST	1581.0748	3691.2019	1529.3037	1695.4598	6985.5035	1714.6526
	STD	8660.6754	29221.9159	600.7164	364.0046	13179865.8357	599.1658
F16	WORST	1884.1099	2283.8358	1775.3202	2041.9821	2631.6118	2234.0749
	AVERAGE	1705.3944	1814.9102	1647.5307	1761.8586	2428.9696	2078.5403
	BEST	1612.6808	1675.0240	1602.4200	1643.5171	2187.2968	1952.2365
	STD	74.6605	156.2032	58.3401	103.6142	140.9550	65.7681
F17	WORST	2018.4012	2035.0278	1741.9899	1915.8444	2443.2917	1930.1460
	AVERAGE	1848.1348	1910.8404	1723.9229	1807.7611	2160.5021	1867.7808
	BEST	1740.3957	1838.7942	1706.4679	1759.8889	1837.9512	1818.0702
	STD	87.8723	57.5464	10.0700	49.6962	167.2660	33.1343
F18	WORST	49760.1523	681900.8684	14226.3850	135728.7307	533962678.7152	634665647.3193
	AVERAGE	24591.2130	210705.3632	7185.4712	64511.1417	165388172.1147	185214454.9991
	BEST	4159.9881	25120.5492	2680.6584	23433.1446	16414814.4300	171011.0796
	STD	12381.5985	165643.7742	3412.5392	32304.8311	127803083.7206	188934511.7444
F19	WORST	55900.1926	116776.0306	5679.8513	3884.8641	60141848.9793	17050.0611
	AVERAGE	22373.7347	46962.2062	2633.8915	2448.0523	9668878.1133	4090.2412
	BEST	2940.0687	6653.3830	1905.3740	1967.5159	22690.5086	2168.9346

	STD	13907.3118	35110.9179	1151.9552	433.1006	14653229.0686	3330.2050
F20	WORST	2086.7142	2280.1753	2039.8808	2268.9464	2500.3711	2185.1786
	AVERAGE	2063.3257	2171.6234	2019.2384	2130.5784	2355.8805	2137.2706
	BEST	2029.9650	2111.7484	2006.3129	2057.9973	2254.5836	2110.3324
	STD	18.6114	46.6752	9.4352	68.5744	70.7073	22.2044

Table S4. Experiments on GA, PSO, ABC, BA, IA, FA for F21~F30 functions under D=10 on parameters I

Problems	Criteria	GA	PSO	ABC	BA	IA	FA
F21	WORST	2383.3995	2388.4116	2236.6934	2346.6081	2434.0625	2410.7712
	AVERAGE	2322.7791	2311.8950	2216.4960	2279.6901	2368.6747	2365.9808
	BEST	2237.2227	2210.1488	2207.2762	2205.0324	2244.7432	2309.6995
	STD	45.5403	77.6269	7.4739	67.8529	47.3273	32.0313
F22	WORST	3107.6506	3062.5508	2312.7478	2405.1075	3890.9314	3980.0724
	AVERAGE	2426.5363	2771.8942	2284.7347	2379.9976	3080.4960	3540.4211
	BEST	2340.4546	2413.6838	2229.3030	2310.4227	2544.6037	2983.8401
	STD	169.9893	178.6271	32.6404	22.7354	328.6690	322.8674
F23	WORST	2674.8114	2681.1163	2637.8162	2646.6399	2818.8945	2882.9671
	AVERAGE	2654.1820	2655.7074	**2608.2473**	2635.7399	2761.8516	2787.1895
	BEST	2638.4900	2626.9254	**2306.4069**	2625.4418	2708.8149	2721.5366
	STD	9.7890	14.0866	71.5485	5.6332	32.2214	36.6854
F24	WORST	2785.4920	2814.6161	2765.9581	2775.5834	3023.2086	3066.4693
	AVERAGE	2735.2686	2791.6850	2559.4864	2746.9556	2891.9558	2954.1071
	BEST	2649.4631	2684.6849	**2471.6761**	2575.7871	2839.9937	2856.0970
	STD	38.4258	26.6788	73.8090	57.9020	45.7179	51.7922
F25	WORST	3052.1987	3236.9447	2950.5040	2984.0308	3633.6171	4612.3968
	AVERAGE	2968.8807	3131.5508	2905.8115	2964.9468	3414.5731	3977.5321
	BEST	2947.1937	3063.7445	2666.6576	2940.9627	3160.2700	3407.6744
	STD	25.1891	55.1507	59.4794	10.9834	137.6877	385.8667
F26	WORST	3444.2525	4466.0241	3042.7033	4206.2604	4693.8968	4919.7016
	AVERAGE	3123.6607	3410.8699	2917.9066	3235.9838	4079.6370	4437.6681
	BEST	2994.6630	3196.0133	2627.6391	2995.7081	3534.1700	3663.4834
	STD	116.7816	370.3052	94.5920	409.2416	311.2601	295.0645
F27	WORST	3146.9655	3197.6933	3110.8552	3149.3789	3421.9789	3391.7328
	AVERAGE	3121.0589	3118.3969	3099.9426	3107.6904	3267.2788	3330.2157
	BEST	3108.3614	3099.5733	3090.8756	3093.7628	3190.9502	3217.9308
	STD	9.0553	21.7855	5.2998	21.1648	63.5154	43.9582
F28	WORST	3614.7569	3598.0386	3404.0943	3413.6655	4197.2400	4103.4389
	AVERAGE	3495.9560	3401.9032	3225.7791	3321.6842	3827.6599	3906.3532
	BEST	3379.1433	3247.0705	**2875.2363**	3178.5999	3641.5615	3466.7687
	STD	50.6673	101.5109	133.9791	103.7884	155.0943	161.4183
F29	WORST	3245.4526	3426.3549	3254.5656	3464.9360	3978.0528	3543.1881

	AVERAGE	3210.9784	3275.6124	3189.7916	3238.7054	3629.4766	3428.6484
	BEST	3189.8046	3151.6736	**3140.4775**	3147.7312	3318.7383	3331.2090
	STD	15.8149	81.4955	25.2171	76.7546	169.6030	58.2207
F30	WORST	3072936.6765	11210758.89	552790.08	1527340.374	199505652.5509	58588552.6398
	AVERAGE	1082755.3607	1684810.1599	178867.64	655207.9021	41744446.4872	30975573.0320
	BEST	6629.7662	52143.0255	5677.2070	58455.4728	1205508.9873	8379858.2903
	STD	872896.5529	2487976.5590	187681.3651	472315.9407	43532551.5594	14131455.0824

Table S5. Experiments on CS, DE, GSA, GWO, HS for F1~F10 functions under D=10 on parameters I

Problems	Criteria	CS	DE	GSA	GWO	HS
F1	WORST	14233.1777	17589.6524	**785.5710**	2290167.9016	1310786988.6210
	AVERAGE	4075.5846	5953.8654	**246.2784**	145710.2779	337264539.3872
	BEST	858.2665	150.7893	**100.0402**	2044.0964	73807584.4212
	STD	3464.8392	5275.6475	**201.9534**	507470.7393	270821881.0885
F2	WORST	**200.0001**	201.7737	223.8595	691.5691	1570.8417
	AVERAGE	**200.0000**	200.1508	210.9144	294.8109	739.4699
	BEST	**200.0000**	200.0002	200.0004	202.2111	298.1408
	STD	**0.0000**	0.3974	7.7134	102.0191	324.6140
F3	WORST	**300.0001**	12503.6313	9781.6505	4622.2197	39967.2188
	AVERAGE	**300.0000**	8353.7616	7573.7491	980.6740	24546.6108
	BEST	**300.0000**	3729.4307	5667.2279	305.1910	10700.5157
	STD	**0.0000**	2699.7958	1106.2889	1107.4253	7950.6130
F4	WORST	**400.7126**	406.3778	406.3770	469.0736	492.1883
	AVERAGE	**400.1050**	404.1867	406.0047	414.2323	438.1227
	BEST	**400.0052**	401.0683	405.4452	403.0292	414.9925
	STD	**0.1664**	1.4515	0.2380	17.9310	19.0861
F5	WORST	521.9772	**509.9128**	564.6718	522.9500	555.7782
	AVERAGE	513.8265	**506.6136**	552.5062	510.4201	542.7490
	BEST	507.9959	503.5275	539.7982	**502.3771**	530.5926
	STD	3.6416	**1.6994**	6.5187	5.3371	6.8470
F6	WORST	609.8465	**600.0000**	632.8836	603.9241	613.5079
	AVERAGE	604.6775	**600.0000**	617.5319	600.7298	606.1549
	BEST	601.6286	**600.0000**	600.8382	600.0344	603.0252
	STD	1.6299	**0.0000**	10.0255	1.0251	2.2500
F7	WORST	735.7535	721.5861	**714.4581**	742.3353	779.6371
	AVERAGE	727.9220	718.1928	**712.0854**	726.6737	766.5767
	BEST	722.8397	714.7708	**710.8203**	717.6123	754.8371
	STD	3.1227	1.9051	**0.9790**	8.0591	8.2094
F8	WORST	824.2729	**810.3171**	830.8437	823.8071	856.6085
	AVERAGE	816.7423	**807.3141**	820.0484	811.0093	841.5594
	BEST	810.6840	803.9091	815.9193	**803.3253**	822.7602

	STD	4.0809	**1.9776**	3.8368	5.1105	9.0382
F9	WORST	987.2820	**900.0001**	**900.0000**	967.3987	1058.3039
	AVERAGE	933.8048	**900.0000**	**900.0000**	909.8875	982.6798
	BEST	911.9161	**900.0000**	**900.0000**	900.0975	924.6963
	STD	18.0020	**0.0000**	**0.0000**	19.6313	36.9898
F10	WORST	1725.2153	**1564.6709**	3460.9346	1813.4370	3202.9820
	AVERAGE	1527.3971	**1413.7491**	2764.3389	1468.9291	2927.5937
	BEST	1384.4228	1271.7443	2279.2205	1130.1008	2538.5848
	STD	**81.9566**	96.1555	351.4215	244.1845	169.0652

Table S6. Experiments on CS, DE, GSA, GWO, HS for F11~F20 functions under D=10 on parameters I

Problems	Criteria	CS	DE	GSA	GWO	HS
F11	WORST	**1105.7660**	1105.7971	1151.2800	1135.6050	5022.8180
	AVERAGE	**1103.4890**	1104.0129	1131.5425	1118.0141	2325.6969
	BEST	1101.7900	**1101.3704**	1120.6729	1102.8143	1313.3024
	STD	**0.7746**	1.2477	9.3359	10.1489	850.8032
F12	WORST	**10705.2573**	640770.6102	1043728.6444	2304166.6132	60463726.2643
	AVERAGE	**5134.2698**	207331.0752	490716.7255	483537.7758	17386067.0036
	BEST	**3430.5345**	39281.7505	60363.9618	5738.9561	3531238.7047
	STD	**1601.8829**	139808.3189	274456.7550	737717.4004	15065381.7795
F13	WORST	**1331.7661**	8095.1641	14581.2820	21777.6479	807784.5468
	AVERAGE	**1320.6429**	2837.2704	11475.7956	9483.2242	107074.9092
	BEST	**1310.8958**	1521.8267	8733.2086	1734.0961	2565.8365
	STD	**4.9694**	1515.9030	1779.7516	6107.9357	207807.3983
F14	WORST	**1424.0463**	1440.6531	7264.0505	5138.3179	17316.7924
	AVERAGE	**1417.4328**	1421.2708	5753.4619	2171.6217	6633.8174
	BEST	1410.4440	**1401.6541**	3567.9372	1442.6071	1804.7889
	STD	**4.0560**	14.8257	987.0154	1450.8913	4506.0960
F15	WORST	**1508.5940**	1558.2196	23656.4746	6402.5519	52567.8008
	AVERAGE	**1505.5851**	1513.0359	17955.9153	2690.4346	10113.1895
	BEST	1503.2022	**1502.9741**	10304.6742	1533.7577	1905.6469
	STD	**1.4880**	14.5767	3487.8542	1475.4496	11965.4781
F16	WORST	**1606.0069**	1642.7469	2299.7861	1989.1093	2075.1343
	AVERAGE	**1603.5827**	1618.1889	2161.0876	1702.0242	1909.9978
	BEST	1602.0553	**1601.0955**	2008.2307	1603.1961	1652.7973
	STD	**1.1455**	13.1978	89.9219	116.4073	118.1956
F17	WORST	1730.3132	**1727.1040**	2038.7159	1780.0294	1911.2329
	AVERAGE	1725.9379	**1718.3305**	1822.0978	1743.8587	1790.0243
	BEST	1721.4572	**1702.6249**	1749.8039	1723.4557	1733.9237
	STD	**2.4640**	8.8022	95.8370	14.0416	50.7967
F18	WORST	**2034.4934**	3545.4771	16013.0753	53711.4207	1562573.3711

	AVERAGE	**1932.8899**	2345.6735	7813.9717	30273.5435	205340.5501
	BEST	1862.9064	**1822.3515**	3354.6342	2459.5502	8705.5525
	STD	**50.1570**	544.3537	3089.9456	14728.8071	358385.0949
F19	WORST	**1907.1329**	1912.4021	45113.6722	13703.3161	21433.3169
	AVERAGE	1905.1992	**1904.9616**	31762.2989	4788.0776	6960.3624
	BEST	1903.2587	**1900.2891**	9933.5533	1915.5765	1991.1935
	STD	**0.9630**	3.8949	9059.0447	5074.5518	5241.4881
F20	WORST	2027.1857	**2020.0701**	2334.9750	2117.1110	2069.6212
	AVERAGE	2023.7020	**2008.7991**	2256.1712	2042.0310	2040.5744
	BEST	2015.0772	**2000.0000**	2184.5983	2001.2692	2025.0200
	STD	**3.4358**	7.2075	46.9631	27.5174	12.3403

Table S7. Experiments on CS, DE, GSA, GWO, HS for F21~F30 functions under D=10 on parameters I

Problems	Criteria	CS	DE	GSA	GWO	HS
F21	WORST	**2203.3038**	2261.4266	2376.0687	2326.0860	2357.0939
	AVERAGE	**2202.1734**	2227.5559	2351.5527	2286.4618	2346.3917
	BEST	2200.8895	**2195.8115**	2312.9625	2200.7720	2328.6834
	STD	**0.7034**	16.8024	15.4498	50.3944	7.1709
F22	WORST	2301.5094	2312.8103	**2300.0000**	2315.1597	2379.6791
	AVERAGE	**2261.7179**	2274.0882	2300.0000	2300.4595	2337.1244
	BEST	2229.0184	2245.2173	2300.0000	**2211.4188**	2316.1418
	STD	29.0084	20.3614	**0.0000**	21.2943	15.5182
F23	WORST	**2619.7398**	2620.8530	2803.9100	2632.6590	2657.0341
	AVERAGE	2614.5752	2615.6108	2716.2573	2612.9657	2643.1545
	BEST	2610.2301	2611.8498	2668.6773	2606.2089	2627.2831
	STD	2.4322	**2.3720**	35.5893	7.8732	7.5852
F24	WORST	**2609.1200**	2748.5300	2805.6592	2757.0103	2796.4827
	AVERAGE	**2512.0808**	2647.8613	2530.5158	2740.6903	2771.6671
	BEST	2499.7799	2533.2837	2500.0000	2700.0318	2695.4249
	STD	23.3365	64.0913	93.9257	**13.3957**	20.8507
F25	WORST	**2897.7450**	2907.2550	2943.4587	2948.7180	3000.2722
	AVERAGE	**2696.1330**	2902.4491	2943.4072	2932.1744	2966.1288
	BEST	**2606.5377**	2899.4363	2943.3854	2898.2724	2948.9076
	STD	105.6240	1.8283	**0.0160**	19.3456	11.6660
F26	WORST	**2812.6105**	2970.8473	4262.7421	3103.8741	4482.3144
	AVERAGE	**2689.5592**	2835.3325	3092.9736	2909.4141	3679.2310
	BEST	**2602.2976**	2670.2209	2800.0000	2818.1834	3159.9849
	STD	98.6713	113.1683	555.2468	**51.6281**	426.7441
F27	WORST	3090.2130	**3081.5671**	3279.4988	3103.1621	3132.2014
	AVERAGE	3087.9813	**3074.4847**	3229.3362	3093.6139	3114.4357
	BEST	3085.2634	**3070.8567**	3181.8749	3089.3101	3104.5276

	STD	**1.7058**	3.5214	23.6340	3.3357	8.0865
F28	WORST	**3171.8624**	3279.7599	3383.7452	3412.7300	3635.8306
	AVERAGE	**3122.4297**	3272.9084	3376.8636	3338.7800	3520.0120
	BEST	3100.8481	3272.5005	3246.1124	3165.3218	3457.6124
	STD	24.9811	**1.6169**	30.7756	100.6271	54.4637
F29	WORST	3185.6997	**3181.5116**	3616.0748	3201.3438	3467.3319
	AVERAGE	3170.3759	3168.9722	3362.5987	**3165.8671**	3337.3840
	BEST	3152.8914	3147.3843	3263.0803	3143.6933	3241.2195
	STD	**9.6106**	9.8150	79.7743	16.3885	56.3242
F30	WORST	32230.7770	**5220.7723**	974802.2003	1694512.1873	10045600.7709
	AVERAGE	11249.6413	**3802.5453**	716829.3885	419049.9522	5753949.0405
	BEST	4118.4237	**3277.9005**	525888.3046	6238.0000	1657671.8651
	STD	6746.4145	**565.6909**	110449.8440	644144.1293	2373732.5875

Supplementary Material C

Table S8. Experiments on GA, PSO, ABC, BA, IA, FA for F1~F10 functions under D=50 on parameters I

Problems	Criteria	GA	PSO	ABC	BA	IA	FA
F1	WORST	1.15E+10	2.08E+11	9.85E+07	3.92E+10	1.15E+11	2.08E+11
	AVERAGE	3.52E+09	1.76E+11	5.15E+07	3.17E+10	8.84E+10	1.94E+11
	BEST	3.42E+07	1.37E+11	1.97E+07	2.48E+10	5.96E+10	1.80E+11
	STD	3.58E+09	1.95E+10	2.11E+07	4.45E+09	1.44E+10	9.01E+09
F2	WORST	2630.1836	69961.0934	464.9266	8058.5289	63324.2821	85104.7163
	AVERAGE	1161.6697	43883.6970	397.7584	6763.8491	29067.7442	66839.9077
	BEST	459.1505	21897.5945	316.1495	5315.6348	11848.0673	45189.6344
	STD	637.5184	13164.5830	45.9682	706.2053	12430.1128	11966.3422
F3	WORST	609697.3150	799838.7480	250741.7996	108442.5425	246240.8790	245488.8478
	AVERAGE	357225.5227	508150.5636	222134.8062	75484.9226	205818.6053	224144.6659
	BEST	234725.6472	361969.9330	169186.7319	58058.7077	169782.3312	194245.3742
	STD	87040.1272	106557.3529	21723.6882	12703.6189	19639.0067	15684.4441
F4	WORST	1002.0903	51317.5811	662.3985	4063.2995	39484.5028	74908.6213
	AVERAGE	775.5342	38707.2423	602.6489	2808.5483	22510.4298	66493.1231
	BEST	696.4286	28302.0355	538.1760	2058.2742	13647.6373	58783.0090
	STD	65.2127	6756.5074	35.2840	539.3999	6259.6480	4493.0140
F5	WORST	1063.9642	1622.6180	803.6027	1089.8516	1200.5481	1471.9552
	AVERAGE	1026.2105	1473.4018	758.4043	1017.9668	1110.7023	1418.9024
	BEST	970.8414	1389.3065	696.8648	945.0356	1028.0266	1366.2310
	STD	27.0791	63.6173	30.5942	33.7224	50.6584	26.6224
F6	WORST	694.8013	729.6394	606.2486	671.7973	699.0175	713.4890
	AVERAGE	687.1545	719.3547	603.3754	660.9922	682.4878	709.2457
	BEST	672.5448	708.6088	601.9090	651.1449	666.1774	705.2882
	STD	5.7621	5.5090	0.9356	5.5076	9.3277	2.2881
F7	WORST	2109.2363	5674.2438	1165.5137	2307.1567	1986.1061	5083.9418
	AVERAGE	1702.9699	5167.3011	1121.3564	2078.7286	1845.2772	4873.8013
	BEST	1465.2054	4612.6251	1073.4776	1871.3300	1680.0030	4620.9629
	STD	167.5442	295.0843	27.3060	120.7691	108.2505	133.0190
F8	WORST	1394.2189	1836.2366	1095.0179	1389.8781	1482.1620	1792.4511
	AVERAGE	1358.8562	1750.9428	1056.6822	1320.0725	1409.1977	1736.9555
	BEST	1311.2458	1673.1201	987.1964	1260.6791	1342.7254	1676.6093
	STD	21.2832	37.0708	24.7954	31.0063	45.6248	30.5943
F9	WORST	4198.1251	89578.5696	15433.5095	22609.0023	28679.2716	48036.4934
	AVERAGE	2263.8017	64449.2711	12443.5396	15375.9241	21498.9031	43611.5834
	BEST	1418.8323	45440.9967	7813.3345	9810.5055	15828.9594	37786.2325
	STD	714.7284	12111.9492	2069.6197	3470.8475	2795.7986	2659.6917
F10	WORST	13570.9779	14917.3483	**7191.2271**	14884.4136	14057.3827	14503.4267

	AVERAGE	12782.0875	13871.3832	6520.9602	14174.2205	12425.7911	14088.4691
	BEST	12115.1652	12541.6909	5860.4629	13037.5499	11479.6629	13436.4445
	STD	298.8837	667.8415	416.3968	469.4655	640.7047	261.0130

Table S9. Experiments on GA, PSO, ABC, BA, IA, FA for F11~F20 functions under D=50 on parameters I

Problems	Criteria	GA	PSO	ABC	BA	IA	FA
F11	WORST	30555.7454	64998.8020	8604.2089	7915.2986	49745.9818	30616.8427
	AVERAGE	16531.7296	39171.6324	4445.6691	4868.5839	26390.0845	25779.5944
	BEST	3724.2206	24986.5905	2092.9880	3137.3655	13836.8520	19294.1337
	STD	8959.7814	11329.5137	1438.4441	934.9020	8970.0158	3183.3412
F12	WORST	9.2287E+07	5.7842E+10	5.0862E+07	1.0098E+10	8.3043E+10	1.0466E+11
	AVERAGE	3.1509E+07	4.3772E+10	2.9903E+07	7.2143E+09	4.8610E+10	8.7802E+10
	BEST	9.6178E+06	2.7776E+10	1.0845E+07	5.4806E+09	2.0730E+10	6.3454E+10
	STD	1.9823E+07	8.6710E+09	9.9170E+06	1.0827E+09	1.5241E+10	1.2636E+10
F13	WORST	1.75E+09	3.20E+10	3.11E+06	3.34E+09	5.17E+10	5.70E+10
	AVERAGE	1.47E+08	1.70E+10	1.36E+06	2.55E+09	3.82E+10	4.44E+10
	BEST	4.14E+05	9.57E+09	4.39E+05	1.76E+09	2.42E+10	3.76E+10
	STD	3.87E+08	6.10E+09	7.65E+05	4.67E+08	8.70E+09	4.61E+09
F14	WORST	1.5059E+07	71876978.2999	2619197.4530	1.4593E+06	1.8785E+08	4.9672E+07
	AVERAGE	6.6103E+06	15610935.4537	1338328.7670	9.0724E+05	9.6261E+07	2.1686E+07
	BEST	376611.6645	2219745.6821	226652.2209	2.1855E+05	1.8342E+07	1.0127E+07
	STD	4158711.1390	18766590.3343	656097.7723	3.7442E+05	4.6425E+07	1.1179E+07
F15	WORST	3.1154E+06	8.0360E+09	1236234.6520	1.12E+09	2.34E+10	1.39E+10
	AVERAGE	361118.5569	3.1240E+09	462588.7692	6.61E+08	1.14E+10	1.06E+10
	BEST	50573.2293	1.4585E+09	126579.0185	3.69E+08	4.30E+09	5.84E+09
	STD	676071.4986	1.5514E+09	305981.6276	2.05E+08	4.42E+09	2.11E+09
F16	WORST	4299.1178	7803.5421	3703.8239	5670.2176	9784.8314	10268.0612
	AVERAGE	3612.1744	6880.5069	3163.5538	5004.0648	7117.5242	8969.9441
	BEST	2764.0783	5953.3293	2519.2269	4572.8909	5511.4685	8191.5127
	STD	460.4562	483.5784	300.1742	268.0510	1159.3968	457.4493
F17	WORST	4024.5958	69840.7626	3288.2235	5239.7147	62012.3783	50695.4259
	AVERAGE	3222.7260	22901.7614	3060.1157	4727.5824	22830.2196	22031.0043
	BEST	2300.5602	7342.7078	2894.5219	3669.8606	4626.5963	9208.5561
	STD	393.5359	17711.6096	**126.0397**	379.7353	20108.3042	9926.7546
F18	WORST	3.17E+07	2.26E+08	5.15E+06	2.03E+07	4.70E+08	1.27E+08
	AVERAGE	1.17E+07	6.57E+07	2.69E+06	1.03E+07	1.73E+08	7.47E+07
	BEST	982358.2504	19911167.0994	778339.2615	3.1092E+06	4.4159E+07	4.6064E+07
	STD	8466361.7220	56270437.5344	1228021.7460	4.5061E+06	1.0824E+08	2.0690E+07
F19	WORST	9118373.4420	4.5185E+09	349055.2451	4.7982E+08	9.9806E+09	7.0271E+09
	AVERAGE	953475.4028	2.3000E+09	203305.0359	3.2058E+08	5.6239E+09	4.7235E+09
	BEST	15605.7538	8.5125E+08	24411.1902	1.9140E+08	1.2132E+09	2.1683E+09

	STD	2047383.6790	1.0206E+09	85894.8537	8.0856E+07	2.6401E+09	1.1424E+09
F20	WORST	3819.7110	4588.0392	3229.1906	4202.7592	4935.8382	4023.1671
	AVERAGE	3497.9397	4208.1546	2936.9495	3754.9202	4460.3035	3846.2905
	BEST	3107.4390	3695.5248	2405.5544	3456.3644	4079.2094	3640.2597
	STD	186.8896	228.3120	210.0079	196.0929	251.6456	**89.8943**

Table S10. Experiments on GA, PSO, ABC, BA, IA, FA for F21~F30 functions under D=50 on parameters I

Problems	Criteria	GA	PSO	ABC	BA	IA	FA
F21	WORST	2970.6277	3315.7557	2617.0560	2881.1266	3048.3489	3333.6841
	AVERAGE	2930.5201	3203.1208	2576.8571	2804.1044	2934.8968	3268.1014
	BEST	2881.8476	3120.6501	2529.0108	2735.2119	2809.2467	3192.4711
	STD	25.2088	48.5644	22.6817	40.6593	72.8183	37.7581
F22	WORST	15305.0861	16719.2738	**9076.1567**	16488.0774	15871.1124	16210.1051
	AVERAGE	14492.9772	15250.2298	8224.8142	15768.3599	14611.6729	15802.1631
	BEST	13619.4550	14423.4310	7065.6044	15034.4223	13196.1348	15127.3306
	STD	508.1153	723.0329	513.7224	426.0571	856.9585	287.6048
F23	WORST	3552.3511	4105.9972	3109.4953	3298.0218	4236.3205	4738.7292
	AVERAGE	3441.2808	3878.0850	3046.1689	3249.6314	3999.1496	4588.3645
	BEST	3372.7435	3715.9012	2981.1774	3183.1404	3612.9215	4359.3143
	STD	48.8230	116.3666	37.2542	26.4210	148.1612	102.5072
F24	WORST	3661.9653	4152.6961	3754.7899	3420.3124	4568.1748	5349.8123
	AVERAGE	3584.4356	3875.3554	3584.9933	3366.9236	4377.3174	5114.4496
	BEST	3522.9748	3676.1691	3479.8466	3310.8416	4125.6367	4884.6487
	STD	36.8707	138.2199	83.4934	32.6894	136.0074	115.9334
F25	WORST	3867.0355	50591.5888	3161.0573	5616.2640	16298.5236	41122.3945
	AVERAGE	3614.5671	35970.5457	3105.3323	4981.1325	12688.8251	36992.1953
	BEST	3493.4358	27898.5677	3034.0900	4277.0781	9306.2472	33835.8363
	STD	95.1332	5904.3163	26.6389	413.6734	2199.8857	2302.1746
F26	WORST	9595.9930	18727.2635	8132.0977	9764.4633	18135.1015	26209.6781
	AVERAGE	8994.8341	15928.4327	6850.0863	9168.1036	15115.5720	24034.7683
	BEST	8225.2988	13236.0103	3656.6354	8566.3686	12935.5421	22771.8498
	STD	352.8107	1464.1351	1060.2870	303.4838	1234.8064	911.6484
F27	WORST	3992.1781	4873.5988	3510.0864	3781.2338	6760.6593	7183.7432
	AVERAGE	3866.3328	4341.3224	3449.4407	3559.4422	5993.8858	6766.6958
	BEST	3728.8231	3692.5226	3379.0995	3426.8292	4898.6529	6391.5922
	STD	69.7153	293.6257	35.4134	91.9191	463.7360	271.5025
F28	WORST	4248.7610	16137.4790	3431.1360	4805.7488	12623.4491	19664.9048
	AVERAGE	3937.8973	12357.2644	3370.0006	4355.3049	10450.1143	18183.0075
	BEST	3781.9206	10197.2315	3293.5375	3946.3806	7977.4782	16089.8735
	STD	116.7470	1377.6597	33.1999	206.0410	1187.3975	998.2653
F29	WORST	4646.1388	14468.9624	4391.6559	7506.6945	116650.0367	86074.0103

	AVERAGE	4228.6134	9699.7186	4138.6346	6162.0245	33665.1837	41271.2405
	BEST	3726.4604	7888.3082	3764.1914	5473.4611	7414.0877	18967.3676
	STD	249.3349	1734.3438	176.4197	468.4095	32574.8600	16068.8959
F30	WORST	4.3314E+06	5.6656E+09	2.6934E+06	9.2608E+08	1.2794E+10	1.0684E+10
	AVERAGE	2.8795E+06	2.8491E+09	1.6924E+06	6.4065E+08	6.5933E+09	7.5141E+09
	BEST	1.5853E+06	8.3692E+08	1.2138E+06	4.0143E+08	2.2890E+09	5.3812E+09
	STD	7.0052E+05	1.3093E+09	4.3336E+05	1.3589E+08	2.9616E+09	1.1008E+09

Table S11. Experiments on CS, DE, GSA, GWO, HS for F1~F10 functions under D=50 on parameters I

Problems	Criteria	CS	DE	GSA	GWO	HS
F1	WORST	5019.399577	13731.59286	**4854.004153**	9103167715	23808787.37
	AVERAGE	**1143.542341**	5593.337216	1270.585353	4817920639	18706543.28
	BEST	123.9051449	873.0697854	**100.0351925**	734891372.1	13686192.93
	STD	1549.220759	4263.593933	**1259.974096**	2377275277	2756487.371
F2	WORST	200.0025	2508.3802	**200.0005**	1974.7793	1490.2158
	AVERAGE	200.0022	1584.0207	**200.0003**	1416.5266	962.9948
	BEST	200.0016	655.5803	**200.0003**	847.1604	718.3262
	STD	0.0002	575.4537	**0.0001**	322.0299	194.4486
F3	WORST	**35513.8408**	290220.4289	162794.3989	88725.7864	380802.8375
	AVERAGE	**30556.6259**	178300.0851	142423.1048	63191.6997	309668.4963
	BEST	**23335.6498**	75728.0424	122650.8243	39661.3200	201802.6338
	STD	**3231.9410**	67623.5131	10617.1037	15954.5958	44134.9967
F4	WORST	499.7741	**448.6336**	595.6381	1246.8938	666.2820
	AVERAGE	**444.2842**	445.9746	518.1847	827.0265	615.0293
	BEST	**426.3077**	444.4458	428.5127	624.7922	540.5747
	STD	21.9202	**0.8845**	41.4049	149.9551	34.4209
F5	WORST	818.4900	728.7132	850.2229	**709.6835**	843.7751
	AVERAGE	783.4911	698.0146	823.2600	**667.5045**	676.2427
	BEST	732.6594	665.6601	782.5667	639.3635	**568.5427**
	STD	26.2829	18.8940	**17.2749**	18.9106	87.3930
F6	WORST	659.1498	**600.0000**	658.4497	613.6512	606.8287
	AVERAGE	648.3113	**600.0000**	650.1581	608.3309	603.2226
	BEST	638.3152	**600.0000**	640.8974	601.9816	601.6936
	STD	6.2088	**0.0000**	4.1785	3.4111	1.3148
F7	WORST	1101.5316	997.8648	**818.1593**	1044.0531	1215.2829
	AVERAGE	1020.4381	957.6188	**789.1432**	974.5407	1151.5824
	BEST	965.2413	912.7168	**773.3115**	896.6980	1030.0316
	STD	33.0585	26.1531	**10.5079**	37.3839	47.8639
F8	WORST	1150.8748	**1037.1801**	1166.1422	1070.0558	1149.3401
	AVERAGE	1093.3177	1003.5322	1142.9600	982.3760	**924.7647**
	BEST	1022.4460	970.9222	1108.4355	935.6918	**853.3344**

	STD	34.1885	21.5236	**14.7506**	36.7209	92.0858
F9	WORST	19961.1751	3471.7104	5865.2357	10667.5938	**2479.0426**
	AVERAGE	14059.4314	**1355.8390**	4586.3377	4471.2094	1853.9088
	BEST	8095.2709	**900.5945**	3599.1595	2036.0158	1195.2417
	STD	2848.1163	747.5491	651.7507	2256.7808	**400.7461**
F10	WORST	7362.4138	10180.8247	8830.5155	7724.5665	15625.4748
	AVERAGE	7066.1550	9185.2654	7390.7770	**6240.9936**	14945.2187
	BEST	6451.3279	8480.0201	6523.2579	**5185.1252**	13911.7385
	STD	**231.3603**	528.1585	636.6554	587.3618	445.8150

Table S12. Experiments on CS, DE, GSA, GWO, HS for F11~F20 functions under D=50 on parameters I

Problems	Criteria	CS	DE	GSA	GWO	HS
F11	WORST	1256.4925	1272.7944	**1247.6112**	8485.4120	12658.3415
	AVERAGE	1230.7902	**1205.2123**	1222.0223	2995.4541	6215.3391
	BEST	1203.4215	**1136.1366**	1204.7225	1433.0228	1822.6576
	STD	14.5527	39.9526	**10.7532**	1582.2245	2886.5663
F12	WORST	**1127842.779**	56685862.78	1414442.107	1520752626	78183311.88
	AVERAGE	**672424.9686**	26437089.96	855635.6488	294383191.8	34678421.88
	BEST	**268554.0601**	3947914.103	515720.5835	19049338.32	12786992.11
	STD	250428.9051	16784841.01	**226006.1909**	343565070.4	17752669.1
F13	WORST	24397.1999	67743.5178	**22133.4028**	733701771.6000	1142862.6210
	AVERAGE	**9677.9830**	21203.3802	17921.5443	135021417.8000	843206.5263
	BEST	**2751.1694**	5790.7626	14967.2879	63486.6078	495415.3774
	STD	7467.5832	15943.7785	**2381.7263**	178988003.7000	142481.4624
F14	WORST	**2267.2151**	1247809.8400	40013.0854	434759.3290	9665447.8550
	AVERAGE	**1881.0893**	530715.5570	19784.9556	192108.3809	2009212.9630
	BEST	**1706.0756**	170167.4699	11095.5617	30739.6042	124466.1859
	STD	**186.0523**	305928.9918	7266.2912	138234.8893	2176854.9560
F15	WORST	**5091.7634**	39102.5097	17321.9858	26990171.9500	204336.5002
	AVERAGE	**2705.2721**	15957.5762	12211.5594	4645056.2300	147926.1275
	BEST	**1974.0867**	3264.2095	7022.9687	14284.0550	104990.3422
	STD	**810.2771**	10769.5596	3477.9936	8683460.8300	28453.8141
F16	WORST	3405.8413	**3279.4340**	3968.4809	3299.0517	5269.1610
	AVERAGE	3187.8035	2836.6620	3520.8892	**2836.3719**	4844.4939
	BEST	2901.2676	2282.5166	2757.7385	**2224.9640**	4152.4415
	STD	**127.0180**	251.4863	325.1560	256.7214	322.6061
F17	WORST	3003.2775	2950.8677	4139.0874	**2845.3096**	4111.8163
	AVERAGE	2810.0084	2665.4146	3560.2046	**2620.4479**	3731.8330
	BEST	2481.8839	2216.1170	3086.3096	**2163.6668**	2433.7206
	STD	156.5652	226.9739	336.6568	167.8344	372.7768
F18	WORST	**197978.0572**	2384173.6590	307262.3202	17332150.1300	37760674.7100

	AVERAGE	**141364.6498**	1339528.7440	184493.7265	2186183.3760	18235029.4200
	BEST	**72474.5628**	304312.7360	90318.6027	138681.5682	2608617.7540
	STD	**33577.2560**	495027.4796	48953.1106	3668607.2520	9763840.4850
F19	WORST	**3623.8097**	10516.3237	21947.8249	8727111.4300	111708.3029
	AVERAGE	**2350.7099**	7571.8018	16936.0272	1598319.6350	77858.6376
	BEST	**2023.5797**	4768.4602	11366.8130	39042.6818	44938.6624
	STD	**338.6595**	1725.2511	2597.7610	2939551.3510	21048.5868
F20	WORST	3169.7028	**2683.3248**	4161.9016	3159.7169	4355.9163
	AVERAGE	2898.5694	**2489.0004**	3440.7617	2680.4872	4003.3221
	BEST	2663.3383	**2239.3789**	2901.2167	2374.2686	3665.8884
	STD	119.2373	125.3900	269.0799	218.9314	194.0833

Table S13. Experiments on CS, DE, GSA, GWO, HS for F21~F30 functions under D=50 on parameters I

Problems	Criteria	CS	DE	GSA	GWO	HS
F21	WORST	2607.3249	2552.7589	2753.4699	**2523.8537**	2659.1014
	AVERAGE	2564.2783	2518.3894	2687.7899	**2474.0013**	2573.9167
	BEST	2488.7192	2475.4391	2636.8722	2429.5137	**2377.2626**
	STD	33.0401	**18.7318**	30.9734	24.5615	91.1159
F22	WORST	9531.8957	11853.9031	12021.2434	9343.8251	17079.8188
	AVERAGE	8959.7994	10755.4056	11149.4412	**8202.8470**	16338.8772
	BEST	8615.8542	**4469.1726**	10079.7740	6677.0551	15277.4374
	STD	**230.6388**	1550.2496	502.0526	650.6074	503.1918
F23	WORST	3039.4170	2932.6102	4342.1717	2964.0557	**2872.1435**
	AVERAGE	2993.5997	2901.9881	4021.9049	2909.0850	**2834.1074**
	BEST	2946.5063	2867.9786	3824.8555	2834.0203	**2802.8153**
	STD	24.0437	20.1130	129.7924	32.8177	**18.1618**
F24	WORST	**3194.6120**	3330.8977	3619.6175	3348.0622	3317.8242
	AVERAGE	3148.4686	3280.7747	3538.5860	**3076.6011**	3279.4091
	BEST	3104.4223	3209.2159	3438.1686	**2999.1116**	3251.3010
	STD	23.4474	37.3563	55.5011	83.0537	**18.1563**
F25	WORST	3019.9450	**2931.2757**	3082.2015	3681.1651	3246.5385
	AVERAGE	2975.1062	**2931.2167**	3053.8875	3333.2251	3105.9637
	BEST	2944.2583	**2931.1563**	2963.1917	3146.3438	3025.4649
	STD	21.8790	**0.0367**	30.6097	157.8912	52.0988
F26	WORST	7183.1980	5683.9697	**2900.0000**	6437.7451	5064.7051
	AVERAGE	4852.8814	5333.7167	**2900.0000**	5827.0137	4725.6591
	BEST	2944.6718	4832.6575	**2900.0000**	5221.1038	4335.6432
	STD	1590.5749	189.8246	**0.0000**	353.1515	216.0491
F27	WORST	3353.0500	**3200.0113**	6201.6513	3693.4801	3552.2291
	AVERAGE	3286.1705	**3200.0110**	5373.0709	3474.6478	3455.4650
	BEST	3228.8344	**3200.0103**	4858.8926	3401.4624	3350.1761

	STD	37.5202	**0.0002**	341.5696	70.2619	55.5717
F28	WORST	3306.2232	**3300.0112**	3314.4492	4607.8553	3902.6068
	AVERAGE	**3290.8306**	3300.0109	3296.8133	3946.3150	3628.0022
	BEST	**3257.6231**	3300.0102	3258.8487	3552.7303	3441.0911
	STD	19.6976	**0.0003**	14.3911	278.4031	128.2537
F29	WORST	**4265.7617**	4347.5235	5337.9879	4839.9663	5217.3585
	AVERAGE	4130.9539	**3975.4678**	4999.5290	4114.5251	4062.9886
	BEST	3913.9026	3521.0306	4501.8473	3638.7828	**3412.1197**
	STD	**82.0980**	252.3177	231.9748	310.6121	494.5997
F30	WORST	1534536.792	**322381.2344**	7797051.886	160152601.7	5084118.02
	AVERAGE	1083491.397	**131633.1067**	7059991.648	71443359.38	4007927.406
	BEST	777974.562	**22415.03922**	6458705.602	33194299.81	2598702.666
	STD	193673.2045	**99353.50603**	401906.7482	35611073.29	601402.3194

Supplementary Material D

Table S14 Experiments on GA, PSO, ABC, BA, IA, FA for F1~F10 functions under D=10 on parameters II

Problems	Criteria	GA	PSO	ABC	BA	IA	FA
F1	WORST	1.72E+07	2.51E+07	1.32E+07	2.51E+09	2.03E+10	3.17E+10
	AVERAGE	2.20E+06	1.86E+07	4.79E+06	1.54E+09	1.07E+10	2.13E+10
	BEST	2.35E+05	1.06E+07	2.85E+05	7.12E+08	2.69E+09	1.09E+10
	STD	3.66E+06	4.18E+06	3.61E+06	4.98E+08	4.59E+09	6.80E+09
F2	WORST	620.2018	226.4656	421.7721	807.3159	20782.7356	19100.2957
	AVERAGE	379.0324	211.3775	269.5622	603.4011	6858.7792	10274.3262
	BEST	230.5703	207.8677	208.7685	453.2679	896.1372	2310.0012
	STD	90.2186	4.1414	52.0523	93.5562	5852.3112	4784.4153
F3	WORST	48950.2657	377.7648	19999.3084	10485.1563	77441.3250	28324.6109
	AVERAGE	22618.0631	351.7427	13020.6670	6343.2395	31143.9367	22680.7002
	BEST	3783.3361	323.8057	6944.5646	2951.2262	11349.9426	15104.7188
	STD	12663.0918	13.4729	4003.8649	2097.3069	19258.7633	4188.4753
F4	WORST	482.7545	476.8150	470.3901	524.0823	2706.6217	4980.6238
	AVERAGE	444.2229	411.2743	412.2447	471.1171	1472.4049	2505.9436
	BEST	412.6303	402.5273	404.8995	439.2745	651.9300	985.3608
	STD	25.8376	15.6313	14.5081	23.1885	668.3813	1131.5514
F5	WORST	594.5037	552.1705	528.2840	554.7245	637.4059	642.5146
	AVERAGE	557.7127	536.9861	520.3040	547.9885	594.1289	623.1782
	BEST	541.5183	524.4867	512.0314	541.9937	557.1627	596.5947
	STD	12.6698	7.2295	5.2089	3.2715	19.0117	11.7623
F6	WORST	645.5053	610.9161	605.0235	650.8179	690.5463	673.8541
	AVERAGE	635.7167	605.9595	601.7512	629.0364	659.8818	663.5163
	BEST	620.9014	602.1307	600.4847	617.1400	643.0054	656.3987
	STD	7.0730	2.3343	1.0173	7.8629	13.9250	4.8998

F7	WORST	912.8540	745.6081	759.4342	869.4248	862.9237	1191.4556
	AVERAGE	824.4512	739.0163	741.8699	833.1616	842.7123	1094.9558
	BEST	786.8627	732.9580	729.7402	777.4377	796.9956	995.0074
	STD	36.4897	3.8605	8.4918	23.0459	20.5600	58.6935
F8	WORST	907.0640	842.3781	832.6795	867.0500	920.4991	909.1908
	AVERAGE	865.3575	827.8161	820.9819	855.7420	892.9714	897.0352
	BEST	842.4390	818.0895	808.4897	844.0228	872.4565	872.5030
	STD	14.4199	6.9651	5.8501	5.5397	14.4812	8.9287
F9	WORST	1750.5047	908.7855	1262.3747	1626.0206	4128.6948	2783.0071
	AVERAGE	1117.8526	905.5558	977.8729	1428.8061	2321.4507	2367.9278
	BEST	916.5277	902.7885	901.4372	1101.8554	1367.3227	1982.6069
	STD	252.5027	1.4775	84.4555	138.3544	684.1111	243.7720
F10	WORST	2418.5017	2218.3085	1930.2247	2854.2843	3650.5490	2737.0893
	AVERAGE	2063.8674	1954.0078	1603.3122	2325.8570	3105.3384	2568.1092
	BEST	1714.9456	1573.5261	1132.9149	1730.0210	2678.1069	2343.8011
	STD	202.0139	200.2997	216.0494	268.8732	265.8077	116.3733

Table S15. Experiments on GA, PSO, ABC, BA, IA, FA for F11~F20 functions under D=10 on parameters II

Problems	Criteria	GA	PSO	ABC	BA	IA	FA
F11	WORST	4037.4821	1156.5722	1370.7275	1522.5418	24385.5137	12969.0513
	AVERAGE	2174.0563	1132.0821	1165.0612	1354.5407	8353.2900	4263.6737
	BEST	1175.3990	1116.1924	1110.0201	1201.8259	1482.2015	1935.2462
	STD	870.5894	9.7998	60.9754	88.1860	6247.0261	2482.7799
F12	WORST	8.25E+06	6.23E+06	5.26E+06	5.93E+07	2.09E+09	2.89E+09
	AVERAGE	1.76E+06	1.70E+06	1.39E+06	3.02E+07	8.07E+08	1.60E+09
	BEST	3.94E+04	3.72E+05	3.67E+04	3.87E+06	5.93E+07	6.53E+08
	STD	2.17E+06	1.50E+06	1.49E+06	1.73E+07	5.91E+08	6.94E+08
F13	WORST	84588.7166	21490.8789	27014.0592	590333.0490	5.68E+08	2.60E+08
	AVERAGE	16779.9300	11265.2783	10696.9230	181817.9369	1.16E+08	6.50E+07
	BEST	1980.0603	3245.6987	1450.1610	12870.0225	2.04E+06	3.79E+05
	STD	18577.9024	5379.7453	8002.3558	168143.9022	1.34E+08	7.11E+07
F14	WORST	28520.0894	1714.7140	3337.6945	2199.6688	52205923.3257	2376.6788
	AVERAGE	11708.4218	1501.8460	1936.8328	1729.7779	5084187.4081	1624.4445
	BEST	2001.0514	1457.3747	1449.7191	1536.1625	14129.1998	1498.0962
	STD	8702.4732	55.2560	539.4378	160.3311	11664242.3421	201.6546
F15	WORST	24733.1817	3134.6553	7695.6944	8495.6651	64506520.5808	7500.9838
	AVERAGE	9217.6448	2084.4003	3000.9079	3608.7470	9804131.5114	3634.1296
	BEST	1605.2737	1617.6619	1525.1963	1712.3253	11235.1504	1975.0199
	STD	7316.5079	444.9278	1801.9131	1880.3650	15556751.1733	1415.7151
F16	WORST	1968.6782	2002.2392	1873.1774	1870.5312	2956.4111	2545.9691
	AVERAGE	1763.3005	1840.5082	1736.8620	1777.4975	2516.8678	2193.1383
	BEST	1643.2157	1626.9221	1607.4834	1664.2420	2252.5855	2043.2879

	STD	82.8026	93.2390	83.2100	51.3573	165.8699	115.3682
F17	WORST	2088.4884	1866.3015	1791.5456	1986.6794	2534.4223	2101.9442
	AVERAGE	1877.0128	1770.9406	1738.9108	1828.1072	2247.9522	1959.1280
	BEST	1751.7689	1744.7146	1710.5733	1768.8475	1915.6422	1816.4383
	STD	93.8899	26.1898	20.4886	56.2214	174.0131	77.6369
F18	WORST	125590.4853	47418.7494	48063.4765	335294.3567	6.42E+08	1.01E+09
	AVERAGE	39885.1487	17358.1941	13805.6390	147022.2323	1.80E+08	3.57E+08
	BEST	7765.2306	3955.5427	2257.0773	35514.5200	1.63E+06	1.50E+07
	STD	30064.0824	13494.5671	12608.0572	81887.0656	1.73E+08	2.88E+08
F19	WORST	105384.2283	32947.5105	6059.1043	17881.4047	1.27E+08	2.52E+07
	AVERAGE	25012.3301	3772.6376	3106.6893	6203.4387	3.46E+07	1.30E+06
	BEST	5126.3290	1930.0002	1920.2789	2233.7969	6.77E+04	2.27E+03
	STD	23149.9743	6891.5050	1310.9271	4609.5706	3.59E+07	5.61E+06
F20	WORST	2122.2767	2249.3401	2060.9528	2237.1133	2704.3891	2238.1828
	AVERAGE	2093.6119	2105.9744	2034.7185	2140.2372	2401.5719	2170.8424
	BEST	2062.6937	2040.7243	2014.5840	2083.1693	2257.4932	2116.6451
	STD	18.3889	58.7488	13.2951	50.9408	109.8474	38.0139

Table S16. Experiments on GA, PSO, ABC, BA, IA, FA for F21~F30 functions under D=10 on parameters II

Problems	Criteria	GA	PSO	ABC	BA	IA	FA
F21	WORST	2383.6664	2351.7331	2324.4959	2361.9872	2433.1603	2433.4311
	AVERAGE	2329.4202	2307.9176	2235.2340	2288.1882	2379.5349	2392.9446
	BEST	2253.9005	2200.8264	2213.6550	2209.1397	2263.7273	2244.6599
	STD	45.3174	55.1629	30.2953	70.1229	50.3126	42.5355
F22	WORST	3666.5753	2313.9677	2316.8517	3737.3941	3702.8272	4066.3402
	AVERAGE	2491.4775	2304.6520	2293.9515	2534.2558	3112.6705	3747.4094
	BEST	2300.7851	2217.2301	2232.3763	2393.6159	2512.9466	3134.9966
	STD	285.8952	25.1886	26.5692	286.4620	350.5046	300.9343
F23	WORST	2693.5923	2723.5935	2643.2668	2656.3747	2850.3192	2915.2974
	AVERAGE	2667.0841	2655.4026	2631.0413	2643.5294	2785.1359	2818.2927
	BEST	2644.2424	2627.8882	2617.1405	2624.5795	2717.4406	2748.5012
	STD	11.9340	22.5718	8.3798	7.6398	44.4148	40.3767
F24	WORST	2828.3221	2795.1476	2770.1593	2784.0787	2979.2901	3114.7373
	AVERAGE	2786.7387	2714.5386	2645.4603	2769.0073	2902.1641	2982.4910
	BEST	2738.6722	**2479.1624**	2514.7294	2619.9741	2763.6431	2851.5518
	STD	20.3221	109.1810	100.4541	35.2481	48.3858	70.0206
F25	WORST	3197.9132	2951.2394	2952.0462	3041.3684	4278.5860	5385.8293
	AVERAGE	3018.3218	2935.4116	2905.9681	3007.8212	3441.0874	4302.0044
	BEST	2956.5288	2900.4787	2681.5434	2946.0649	3107.2458	3524.2234
	STD	64.2262	20.0651	71.2296	23.6207	254.2840	522.2458
F26	WORST	4173.3170	4122.8505	3152.1819	4344.8127	4889.6006	5120.3438
	AVERAGE	3293.6125	3011.6323	2991.6324	3358.5104	4324.8546	4668.5367

	BEST	3026.4594	2896.3450	2826.1554	3059.0377	3431.6691	3800.4849
	STD	285.5019	268.2144	**81.1300**	472.5890	385.3743	348.9536
F27	WORST	3174.3059	3200.9266	3133.4754	3102.6670	3485.4095	3549.9650
	AVERAGE	3132.4272	3127.9496	3104.3884	3100.5980	3297.2000	3341.3351
	BEST	3110.9745	3091.4512	3096.6610	3097.4336	3160.0558	3183.7829
	STD	16.5994	34.8071	9.0022	1.3876	109.0819	89.4165
F28	WORST	3682.5907	3446.5547	3384.9652	3736.4806	4278.2631	4513.0687
	AVERAGE	3475.4590	3299.0817	3240.0847	3401.6621	3908.1634	4066.0256
	BEST	3311.3452	3115.9075	3122.7221	3232.4115	3317.6368	3649.4133
	STD	107.2597	136.6024	68.9899	101.7592	219.6879	187.7651
F29	WORST	3297.1770	3343.0052	3278.0378	3379.7694	4038.3000	3950.9794
	AVERAGE	3227.3205	3246.6109	3213.6069	3250.4208	3545.2437	3603.6838
	BEST	3191.6828	3183.0534	3185.5247	3193.7286	3334.7097	3369.2902
	STD	28.0459	51.7246	27.3326	48.5503	167.4160	145.1674
F30	WORST	4.52E+06	1.92E+06	1.31E+06	1.53E+06	1.55E+08	1.06E+08
	AVERAGE	1.78E+06	5.13E+05	4.59E+05	5.25E+05	6.03E+07	4.69E+07
	BEST	6.75E+03	1.15E+04	1.68E+04	7.22E+04	9.04E+06	2.17E+07
	STD	1.46E+06	6.79E+05	4.01E+05	4.80E+05	4.59E+07	2.08E+07

Table S17. Experiments on CS, DE, GSA, GWO, HS for F1~F10 functions under D=10 on parameters II

Problems	Criteria	CS	DE	GSA	GWO	HS
F1	WORST	**1076.4756**	5017.3758	1533.4813	4.65E+08	8.93E+08
	AVERAGE	517.6789	975.1618	**331.9382**	2.51E+07	3.71E+08
	BEST	169.8149	104.6067	**100.4503**	1.96E+03	6.96E+07
	STD	**288.1456**	1313.3013	356.1735	1.04E+08	2.03E+08
F2	WORST	**200.0000**	200.0235	200.0003	346.8184	1437.6374
	AVERAGE	**200.0000**	200.0030	200.0002	283.3074	738.7239
	BEST	**200.0000**	200.0001	200.0001	230.3800	479.3638
	STD	**0.0000**	0.0065	0.0001	35.3684	298.1132
F3	WORST	**300.0000**	10723.1440	5826.0969	6035.9473	34164.1058
	AVERAGE	**300.0000**	5023.0775	3762.3300	2084.9336	21572.6330
	BEST	**300.0000**	2278.9283	1774.4782	301.0396	12911.6672
	STD	**0.0000**	2241.6998	1137.1101	1981.7546	5042.3402
F4	WORST	**400.2190**	406.3474	405.2060	463.5713	490.8986
	AVERAGE	**400.0404**	405.2721	404.8663	423.0225	443.4473
	BEST	**400.0000**	403.4840	403.9679	406.8682	417.4061
	STD	**0.0782**	0.8421	0.2941	23.1752	21.3706
F5	WORST	519.4028	**513.4572**	572.4818	535.8384	554.3909
	AVERAGE	512.7719	**508.1124**	557.9676	516.9237	545.3742
	BEST	507.1621	**504.6060**	539.7982	506.9684	528.8184
	STD	3.7850	**2.3020**	9.3168	8.8714	5.9268
F6	WORST	604.7367	**600.0000**	631.3306	601.1598	614.2676

	AVERAGE	602.3379	**600.0000**	617.1124	600.3237	608.0923
	BEST	600.8024	**600.0000**	600.9588	600.0209	603.2879
	STD	1.1074	**0.0000**	9.7344	0.3683	2.9260
F7	WORST	732.8541	725.0128	**716.5477**	734.4778	803.0312
	AVERAGE	724.7176	719.9736	**713.2566**	723.5415	779.9611
	BEST	715.4215	715.7673	**711.5842**	714.5896	743.0608
	STD	4.5268	2.7034	**1.2967**	4.8252	12.2544
F8	WORST	820.6679	**810.3681**	829.8487	823.2668	859.3237
	AVERAGE	813.0465	**807.7905**	821.6403	814.5633	847.1608
	BEST	808.4003	**803.5114**	814.9244	806.0587	835.5997
	STD	3.3070	**1.5529**	4.7648	4.9998	6.5257
F9	WORST	912.9918	**900.0000**	**900.0000**	929.3882	1204.4281
	AVERAGE	903.9051	**900.0000**	**900.0000**	905.0330	1069.1380
	BEST	900.2415	**900.0000**	**900.0000**	900.1115	953.2770
	STD	4.0440	**0.0000**	**0.0000**	9.1005	78.7224
F10	WORST	1859.6543	**1811.7332**	3228.9057	2002.4975	3138.8064
	AVERAGE	1634.6155	1508.0356	2716.0971	**1479.7272**	2845.2813
	BEST	1327.7328	1243.5219	2148.3461	**1125.3745**	2530.6443
	STD	134.6371	124.7584	296.1864	273.3765	**166.1419**

Table S18. Experiments on CS, DE, GSA, GWO, HS for F11~F20 functions under D=10 on parameters II

Problems	Criteria	CS	DE	GSA	GWO	HS
F11	WORST	**1103.4535**	1105.7367	1141.6115	1246.6665	5366.7683
	AVERAGE	**1102.3624**	1103.6712	1130.4248	1130.2942	1972.1574
	BEST	**1101.3024**	1101.7718	1120.8861	1104.3757	1141.9777
	STD	**0.5534**	1.0413	6.1309	39.7118	1111.6315
F12	WORST	**9871.6875**	474220.7550	1090782.8790	2071164.7446	66752732.8937
	AVERAGE	**4260.7200**	173682.5701	228969.6393	571944.6198	21724123.3593
	BEST	**2104.9026**	34541.7217	17171.8552	12150.6858	946015.8839
	STD	**1955.4371**	122570.8224	251104.6842	624442.1407	16085400.4652
F13	WORST	**1329.7357**	3094.5689	15531.3863	38809.8919	235235.7472
	AVERAGE	**1314.1597**	1799.0782	10123.0779	10145.6124	26346.7100
	BEST	**1306.1490**	1311.7012	5572.3250	1593.0440	2318.1061
	STD	**5.7875**	515.0584	2314.0566	8248.7736	50446.7335
F14	WORST	1423.1738	**1422.9671**	9208.9343	5206.9416	27549.7739
	AVERAGE	**1415.4240**	1418.5004	6465.8933	1830.2947	8674.4306
	BEST	1404.0726	**1400.1588**	2424.7236	1446.4773	1519.5658
	STD	**4.7860**	6.4671	1951.0770	1076.4552	7996.1373
F15	WORST	1508.7066	**1503.6013**	22405.2216	10639.6820	19730.8307
	AVERAGE	1504.6297	**1501.3508**	14860.5805	3306.1135	7178.6816
	BEST	1501.9247	**1500.2441**	4685.5189	1542.5037	1954.1079
	STD	1.6828	**0.7568**	4557.9763	2473.2560	5543.2150

F16	WORST	**1619.9466**	1677.6012	2307.4371	1747.8793	2164.1631
	AVERAGE	**1604.7764**	1615.6894	2121.3020	1661.7262	1852.0701
	BEST	**1601.9030**	1601.4423	1971.9240	1611.2905	1610.2833
	STD	**4.0252**	17.7320	107.8830	47.2137	168.0325
F17	WORST	1734.8782	**1728.8660**	2083.8743	1780.2139	1929.6820
	AVERAGE	1728.3604	**1717.8661**	1892.1693	1739.0448	1783.4382
	BEST	1714.2548	**1701.2059**	1751.6342	1723.4893	1734.2012
	STD	**4.2950**	7.5347	112.1760	14.2649	49.5300
F18	WORST	1993.0478	**1825.8604**	16700.2618	52690.6552	3633664.7291
	AVERAGE	1910.7092	**1820.4959**	7923.4067	31902.0974	294984.8247
	BEST	1872.0218	**1808.0896**	2372.5642	6510.8951	5933.9184
	STD	36.3713	**3.2291**	4769.6867	14545.2674	814119.3047
F19	WORST	1908.0176	**1902.2375**	14438.9499	14379.2615	20757.5333
	AVERAGE	1904.4980	**1901.2255**	9642.4892	7673.2315	8897.9013
	BEST	1903.1015	**1900.0202**	4079.5312	1915.6802	2027.2034
	STD	1.2247	**0.7577**	2562.0820	5636.1179	6503.2684
F20	WORST	2032.8012	**2020.3122**	2513.4301	2165.8621	2063.9171
	AVERAGE	2025.1340	**2013.9268**	2300.5517	2055.9091	2040.5637
	BEST	2016.1679	**2000.0000**	2187.5272	2021.4792	2016.9453
	STD	4.1230	**8.3835**	105.3874	46.7961	10.3323

Table S19. Experiments on CS, DE, GSA, GWO, HS for F21~F30 functions under D=10 on parameters II

Problems	Criteria	CS	DE	GSA	GWO	HS
F21	WORST	**2207.7760**	2318.6119	2373.0173	2316.9546	2365.7061
	AVERAGE	**2200.7595**	2258.8043	2353.5436	2312.0586	2351.1191
	BEST	**2200.0247**	2207.2341	2308.4481	2303.1596	2330.8161
	STD	**1.7367**	40.3733	13.3637	3.8869	9.5377
F22	WORST	**2301.0085**	2316.7355	2300.3444	2325.9712	2389.0919
	AVERAGE	**2272.7439**	2294.1542	2300.0172	2301.8063	2348.0831
	BEST	**2200.3377**	2244.0485	2300.0000	2216.1280	2324.7049
	STD	41.1782	21.9606	**0.0770**	21.1477	15.7895
F23	WORST	**2623.0247**	2623.8635	2921.4613	2633.9654	2666.0695
	AVERAGE	2613.2460	**2602.6720**	2753.7341	2618.0580	2651.6917
	BEST	2606.0644	**2342.2329**	2665.4136	2607.0633	2633.5175
	STD	**4.2064**	61.3986	65.1570	8.3883	7.6092
F24	WORST	**2740.7390**	2753.8683	2833.8700	2767.1579	2796.7410
	AVERAGE	**2541.5925**	2714.7336	2577.0220	2745.1314	2784.4970
	BEST	2500.1082	2568.6296	2500.0000	2719.2782	2759.7059
	STD	65.6552	58.7242	137.4647	11.7589	**8.2495**
F25	WORST	**2897.7693**	2908.7463	2943.4275	2949.8678	2982.0717
	AVERAGE	**2830.2786**	2901.3001	2932.2354	2934.7401	2969.2756
	BEST	**2600.8637**	2876.1595	2897.7429	2902.4045	2938.7348

	STD	108.8964	**6.3482**	19.7824	16.3742	10.0762
F26	WORST	**2900.0000**	3055.2155	4353.2858	3920.4319	4293.4237
	AVERAGE	**2781.9601**	2933.3932	3474.9216	2977.7156	3438.4981
	BEST	**2600.0016**	2649.7546	2800.0000	2804.8923	3065.1758
	STD	83.8621	103.0029	601.0464	238.3505	457.7364
F27	WORST	**3090.9235**	3097.3514	3281.8367	3101.1309	3135.7256
	AVERAGE	3088.7899	**3076.3536**	3217.8850	3094.0179	3113.3778
	BEST	3086.0324	**3071.3298**	3159.8206	3090.7550	3102.5831
	STD	**1.3093**	7.3384	30.2043	2.3634	7.4436
F28	WORST	**3198.0805**	3279.8790	3383.7452	3452.3449	3559.8840
	AVERAGE	**3093.3118**	3272.9850	3337.8885	3387.1970	3492.0408
	BEST	**2807.6164**	3272.5005	3142.5767	3166.8217	3451.4252
	STD	85.6125	**1.6751**	94.1646	69.0834	24.8553
F29	WORST	**3192.3787**	3204.8603	3566.5841	3316.1184	3487.4070
	AVERAGE	3176.5524	**3173.3913**	3359.5530	3195.4632	3349.7998
	BEST	3159.3304	3157.1576	3236.3458	**3143.8873**	3235.8554
	STD	**9.8669**	11.9988	121.2545	48.0125	67.8582
F30	WORST	82809.4674	**5540.5133**	463649.7811	2892975.6363	10482758.8654
	AVERAGE	21099.2980	**3794.2750**	286315.8091	367496.6630	5316293.0365
	BEST	5595.3505	**3271.7034**	185114.2406	7542.8492	884189.5561
	STD	19722.7860	**604.9856**	68520.4244	811324.3234	2883209.7467

Supplementary Material E

Table S20. Experiments on GA, PSO, ABC, BA, IA, FA for F1~F10 functions under D=50 on parameters II

Problems	Criteria	GA	PSO	ABC	BA	IA	FA
F1	WORST	5.52E+10	4.39E+09	4.47E+08	7.47E+10	1.02E+11	2.32E+11
	AVERAGE	1.69E+10	1.63E+09	1.82E+08	5.72E+10	8.72E+10	2.12E+11
	BEST	1.87E+09	8.22E+08	8.97E+07	4.06E+10	6.72E+10	1.87E+11
	STD	1.54E+10	1.03E+09	7.86E+07	8.91E+09	8.40E+09	1.12E+10
F2	WORST	2782.8922	1033.1516	826.5971	15827.8700	54822.3725	114077.8953
	AVERAGE	1535.0162	598.4002	569.7591	13133.7590	32403.3999	86243.8099
	BEST	783.9794	464.8501	413.1991	8716.0405	11118.8293	56096.6917
	STD	594.9337	128.2569	89.4477	1750.9338	13372.3934	13351.3703
F3	WORST	533592.9925	**4757.6652**	283023.6727	211751.5856	261502.1075	293392.2710
	AVERAGE	360450.8232	**2752.3789**	234430.8342	136825.5969	187406.9650	246396.4930
	BEST	247109.2389	**2135.9651**	167806.2310	103501.8348	128660.4869	211186.9448
	STD	78942.4245	**563.6748**	25044.1405	28418.6567	30272.9066	21217.7396
F4	WORST	1828.8990	795.6833	732.6160	8583.5715	33701.5203	85375.5026
	AVERAGE	1135.3336	677.3199	666.2757	5720.7029	23723.0695	73052.5659
	BEST	912.1736	605.7730	578.0114	3730.2723	12005.4899	57842.5868
	STD	243.7415	50.4315	39.8513	1408.1111	6207.7553	7541.8522
F5	WORST	1194.1399	1012.1986	866.9814	1171.3619	1174.2913	1517.1914

	AVERAGE	1150.8908	956.3243	823.6456	1113.5197	1086.1935	1471.2551
	BEST	1092.6934	878.5167	791.0720	1063.1667	1004.6972	1384.2181
	STD	26.1023	33.4707	22.6799	34.3148	54.4744	32.5769
F6	WORST	711.5919	672.6796	610.1317	695.3713	692.9603	746.2426
	AVERAGE	699.5832	633.4833	606.6864	677.5325	681.8262	735.9741
	BEST	688.3985	613.0252	603.4044	660.2245	667.7494	715.3807
	STD	6.0323	19.0652	1.7660	7.4258	6.9449	7.1707
F7	WORST	2928.2344	1179.2866	1250.0883	3704.2626	2007.7884	7118.5317
	AVERAGE	2425.6521	1143.9483	1185.6344	3003.2223	1797.3088	6045.7552
	BEST	2124.6944	1105.7473	1118.9204	2446.4701	1549.2024	5199.2272
	STD	213.5904	**19.9434**	46.6045	339.7349	116.3468	425.0864
F8	WORST	1578.9517	1324.3713	1188.7115	1515.8357	1497.3913	2126.6961
	AVERAGE	1477.7033	1254.3281	1116.4288	1408.5623	1400.8012	1974.2669
	BEST	1424.6589	1184.7868	1029.6923	1288.9790	1268.8389	1884.2173
	STD	40.8644	32.7313	43.2545	51.5347	55.3204	61.4622
F9	WORST	**5583.4508**	27506.4820	20264.0495	36295.8888	29208.2062	77934.1237
	AVERAGE	4204.2256	16425.6283	15186.6662	25365.5098	21836.2272	63229.3904
	BEST	2920.8163	1469.6796	8932.9540	18980.5360	14403.2623	47487.5069
	STD	**717.0423**	7576.3715	2979.9226	4748.9219	3334.4259	6653.2661
F10	WORST	13729.5877	12895.3609	8487.5165	15088.4108	14203.1466	16297.8131
	AVERAGE	13350.5961	11828.9022	7675.1253	14498.9649	12593.8334	15594.9358
	BEST	12805.2064	10228.0732	6246.5725	13522.3007	10509.4777	14864.9601
	STD	**243.1707**	734.4923	531.5367	467.6222	996.5649	346.0301

Table S21. Experiments on GA, PSO, ABC, BA, IA, FA for F11~F20 functions under D=50 on parameters II

Problems	Criteria	GA	PSO	ABC	BA	IA	FA
F11	WORST	42023.8404	1771.4980	13198.9035	15595.6062	52584.4303	96170.6263
	AVERAGE	19682.8094	1594.6961	6421.7427	8775.2771	31869.7460	61168.6025
	BEST	3138.1364	1487.5909	2689.2034	6341.2968	17316.4525	41221.1994
	STD	11057.4559	69.6286	2642.4495	2174.6617	11155.1756	13372.7378
F12	WORST	8.70E+07	2.98E+09	1.51E+08	1.84E+10	9.43E+10	2.14E+11
	AVERAGE	4.25E+07	5.52E+08	8.85E+07	1.43E+10	6.02E+10	1.58E+11
	BEST	1.41E+07	2.43E+08	3.24E+07	9.50E+09	2.26E+10	1.14E+11
	STD	2.09E+07	6.02E+08	3.05E+07	2.46E+09	1.89E+10	2.95E+10
F13	WORST	1.08E+09	3.01E+08	1.65E+07	7.38E+09	5.46E+10	1.50E+11
	AVERAGE	3.07E+08	1.01E+08	5.91E+06	4.92E+09	3.49E+10	1.07E+11
	BEST	1.15E+06	6.70E+07	9.42E+05	2.91E+09	1.11E+10	8.04E+10
	STD	3.25E+08	4.85E+07	3.67E+06	1.23E+09	1.21E+10	1.89E+10
F14	WORST	2.01E+07	2.28E+05	4.72E+06	3.23E+06	2.51E+08	8.03E+08
	AVERAGE	8.47E+06	9.75E+04	2.22E+06	1.88E+06	1.34E+08	4.42E+08
	BEST	1.85E+06	4.70E+04	7.56E+05	8.65E+05	2.17E+07	1.23E+08
	STD	5.64E+06	4.83E+04	1.12E+06	5.81E+05	7.08E+07	2.27E+08

F15	WORST	2.28E+07	3.26E+07	3.94E+06	2.61E+09	2.34E+10	4.72E+10
	AVERAGE	1.89E+06	1.89E+07	1.28E+06	1.47E+09	1.03E+10	3.31E+10
	BEST	1.24E+05	1.15E+07	2.17E+05	8.60E+08	4.59E+09	1.06E+10
	STD	4.94E+06	6.14E+06	9.12E+05	4.69E+08	4.55E+09	9.60E+09
F16	WORST	5195.9228	4134.4334	3740.7838	6258.0901	9618.2642	11662.4132
	AVERAGE	4694.3398	3748.1565	3484.3272	5579.6209	7680.6217	10107.3127
	BEST	3899.2611	3104.9768	2972.3926	4748.3437	5337.4751	8869.8509
	STD	353.2299	284.4417	219.8086	344.7406	1226.6435	716.7178
F17	WORST	4245.3284	3342.0289	3764.2402	6154.2024	60698.9996	111842.6232
	AVERAGE	3588.7242	3071.2084	3324.5436	5682.5801	24651.6490	45775.4831
	BEST	2784.2982	2657.2668	2951.3407	5099.0902	4895.2627	20271.3684
	STD	374.3512	192.4273	209.5900	367.7366	15844.6521	25348.1867
F18	WORST	1.83E+07	2.94E+06	1.45E+07	3.55E+07	5.98E+08	2.16E+08
	AVERAGE	9.46E+06	1.11E+06	5.84E+06	1.78E+07	2.70E+08	1.36E+08
	BEST	2.51E+06	2.90E+05	9.85E+05	4.95E+06	5.11E+07	2.84E+07
	STD	5.06E+06	6.13E+05	3.35E+06	8.84E+06	1.27E+08	5.35E+07
F19	WORST	2.12E+07	1.51E+07	2.48E+06	1.30E+09	9.35E+09	8.98E+09
	AVERAGE	3.41E+06	1.07E+07	8.90E+05	8.45E+08	6.18E+09	6.40E+09
	BEST	5.50E+04	4.52E+06	1.94E+05	4.14E+08	2.73E+09	3.49E+09
	STD	6.33E+06	2.79E+06	5.24E+05	2.89E+08	1.97E+09	1.46E+09
F20	WORST	3908.1892	3368.3793	3409.2399	4286.9696	5075.6484	4131.1580
	AVERAGE	3582.5636	3017.4712	3196.1747	3925.1426	4523.0346	3975.5235
	BEST	3146.9237	2724.8876	2849.7969	3530.8759	3898.4568	3781.1601
	STD	224.0137	172.7679	149.6793	190.5818	247.0872	**78.4267**

Table S22. Experiments on GA, PSO, ABC, BA, IA, FA for F21~F30 functions under D=50 on parameters II

Problems	Criteria	GA	PSO	ABC	BA	IA	FA
F21	WORST	3067.2516	2827.4778	2698.4521	2944.8609	3051.0879	3390.1404
	AVERAGE	3001.0288	2745.9920	2636.8520	2883.7604	2955.1159	3326.1440
	BEST	2929.1076	2660.8035	2544.3224	2820.1811	2872.6660	3276.6003
	STD	35.1630	39.8747	43.4486	29.1168	49.6079	30.1564
F22	WORST	15691.3486	14701.7603	10052.5980	16640.3030	15551.4069	16422.5311
	AVERAGE	15154.5426	12335.0426	8421.6470	16030.5488	14613.6840	16074.4863
	BEST	14691.5377	2603.2019	**2484.6669**	15360.9258	13604.1209	15735.5875
	STD	285.8160	3389.3658	2411.3830	435.3442	521.6766	**193.4803**
F23	WORST	3752.0934	3473.5210	3193.7997	3387.2387	4454.9893	4966.7873
	AVERAGE	3611.8158	3304.8618	3127.6773	3320.6175	4060.5993	4721.2806
	BEST	3491.4479	3183.1855	3004.0619	3226.6226	3561.8578	4494.5015
	STD	76.0155	85.5211	44.2970	38.1299	232.5663	137.2579
F24	WORST	3849.8731	3764.7307	3804.3937	3508.7896	4827.2732	5500.6303
	AVERAGE	3735.2371	3518.9576	3658.9288	3419.9685	4419.8556	5222.5306
	BEST	3593.3468	**3339.7439**	3538.5500	3369.0480	4081.8816	4811.9248

	STD	63.7835	121.4349	77.3406	32.7249	201.8217	185.2407
F25	WORST	5065.6496	3198.0601	3251.8809	10195.8987	14257.1282	47503.4543
	AVERAGE	4197.5441	3137.7268	3160.4099	7617.6946	11858.8900	41705.3656
	BEST	3736.3339	3078.8722	3108.2993	5079.5329	9707.0934	36944.5538
	STD	306.5150	32.8146	39.4271	1529.4270	1219.5635	2895.2080
F26	WORST	11342.0892	10169.0677	8514.5987	10947.2356	17598.2626	29393.8561
	AVERAGE	10742.2475	6266.0452	7553.3539	10133.4218	15620.4769	26017.9166
	BEST	10142.2326	3274.0813	4817.0579	9347.6623	13596.0377	23576.7486
	STD	305.5508	2585.4281	761.5220	460.7429	1168.9385	1225.3744
F27	WORST	4244.1636	3699.8288	3611.4196	3933.6564	6442.0521	7722.1762
	AVERAGE	4181.2403	3484.2780	3510.4484	3721.8451	5880.7913	7221.5814
	BEST	4069.5152	3297.7131	3427.3176	3579.7186	4906.6178	6659.3978
	STD	52.4450	124.1141	53.1121	96.9917	418.9433	330.3945
F28	WORST	5693.0836	3831.9357	3509.1879	9138.4255	13050.8180	20971.4850
	AVERAGE	4970.3333	3433.9171	3419.8297	6070.4390	10248.2415	18952.8527
	BEST	4290.0374	3339.6835	3356.6620	4850.6405	8200.8957	17234.3131
	STD	374.6803	132.1411	39.8936	1073.0502	1294.5342	1025.3615
F29	WORST	5598.6883	5769.4020	4537.0274	7860.1918	443303.3837	273275.8639
	AVERAGE	5198.9752	5071.0403	4284.2107	7039.4956	50305.8604	109857.6966
	BEST	4768.3783	4727.1315	3820.4656	6149.7317	7371.5852	29241.8564
	STD	235.4118	318.1987	187.8299	470.0569	93747.4193	60597.5035
F30	WORST	1.28E+07	1.18E+08	7.38E+06	1.67E+09	1.45E+10	1.36E+10
	AVERAGE	6.89E+06	9.99E+07	2.96E+06	1.05E+09	7.63E+09	9.29E+09
	BEST	3.15E+06	8.35E+07	1.85E+06	4.35E+08	1.98E+09	5.45E+09
	STD	3.00E+06	1.08E+07	1.19E+06	3.34E+08	2.82E+09	2.45E+09

Table S23. Experiments on CS, DE, GSA, GWO, HS for F1~F10 functions under D=50 on parameters II

Problems	Criteria	CS	DE	GSA	GWO	HS
F1	WORST	**1.02E+04**	2.28E+06	3.19E+04	1.51E+10	2.09E+10
	AVERAGE	**2.56E+03**	2.05E+05	5.60E+03	6.91E+09	1.59E+10
	BEST	1.11E+02	1.29E+02	**1.08E+02**	1.31E+09	9.54E+09
	STD	**2.95E+03**	5.27E+05	8.11E+03	3.22E+09	2.99E+09
F2	WORST	**200.0040**	31746.9347	1160.7739	3344.8424	18303.8527
	AVERAGE	**200.0014**	12700.4051	406.0398	1731.4057	12783.1568
	BEST	**200.0007**	2532.9496	200.0010	1024.6372	8637.2173
	STD	**0.0008**	7895.1983	245.9467	569.6310	2599.8748
F3	WORST	24646.6848	599286.3290	250255.0595	111247.4266	389498.9094
	AVERAGE	20159.3035	343488.8294	186204.8178	77105.8233	300852.6311
	BEST	14808.0367	149800.6154	128813.1440	44123.6892	229674.3931
	STD	2536.1061	140460.7328	28980.7509	17636.9544	46049.3862
F4	WORST	579.9845	**447.0451**	648.8019	1468.5626	2905.2431
	AVERAGE	**437.5964**	444.1200	512.8025	1015.7883	2361.5973

	BEST	**400.0001**	439.4522	429.7586	699.0085	1852.4819
	STD	44.1811	**2.0625**	62.6456	242.0154	265.2721
F5	WORST	860.1851	885.7122	852.2128	**755.1916**	1023.2403
	AVERAGE	771.7189	826.3423	798.3474	**694.1462**	986.4360
	BEST	702.5253	766.9366	690.0366	**613.7003**	945.6314
	STD	40.2967	34.0013	47.1856	30.8246	**21.0967**
F6	WORST	656.7505	**600.0001**	643.4854	622.6816	625.8855
	AVERAGE	633.4571	**600.0000**	632.0601	612.0397	621.6597
	BEST	620.6616	**600.0000**	617.3588	604.3734	615.7079
	STD	9.0591	**0.0000**	7.6244	4.2024	2.2576
F7	WORST	1194.3518	1134.4017	**993.4898**	1224.7953	1660.9620
	AVERAGE	1090.4073	1061.7652	**940.9815**	1036.2680	1575.7428
	BEST	981.0081	1016.3575	**858.5536**	915.7978	1489.7964
	STD	64.4202	32.0684	42.1220	81.5279	51.6151
F8	WORST	1105.6050	1188.3912	1197.9794	**1038.3122**	1326.5756
	AVERAGE	1072.3991	1133.1929	1090.7749	**984.2740**	1287.7225
	BEST	996.0272	1069.3994	999.9856	**953.3614**	1244.6120
	STD	28.6534	34.0119	45.8231	23.1301	**21.2813**
F9	WORST	19437.7861	11439.0101	14233.5154	6137.3049	10614.0874
	AVERAGE	11788.9383	**2175.8717**	7748.5029	4254.8037	8663.7850
	BEST	4660.4465	**900.0000**	2774.5532	1838.5635	6557.6438
	STD	4163.3156	3020.7771	2607.9157	1125.1342	1150.2789
F10	WORST	7781.1331	13251.2678	**7777.4551**	7803.9818	16179.0376
	AVERAGE	7123.3675	12547.1238	**5914.0745**	6634.7765	15339.7986
	BEST	6402.3487	11869.7523	**4785.6608**	4936.5436	14537.0111
	STD	338.6191	353.7371	742.9287	718.9209	486.3024

Table S24. Experiments on CS, DE, GSA, GWO, HS for F11~F20 functions under D=50 on parameters II

Problems	Criteria	CS	DE	GSA	GWO	HS
F11	WORST	**1278.8586**	1429.5106	1358.0257	8304.7295	16540.5087
	AVERAGE	**1219.6203**	1272.4814	1274.2403	3890.2435	8986.1731
	BEST	**1176.1034**	1183.1005	1223.6732	1569.7636	4587.6185
	STD	**25.5339**	65.7866	38.1853	2029.2717	3099.6564
F12	WORST	**1189378.0401**	913826751.5221	1958638508.2824	3242031151.7423	2873752281.9699
	AVERAGE	**225468.9250**	341901976.4075	198558063.0178	645808708.6912	1954516267.5605
	BEST	**55980.1229**	9197187.1652	220703.8490	14137838.1221	1010017054.3358
	STD	**239295.4412**	285665724.3724	601857300.9132	795758292.4735	466617471.2543
F13	WORST	**12170.2928**	14638693.2685	100951.2753	270296041.1967	34847617.6307
	AVERAGE	**4235.0145**	1576472.9219	53226.9458	88745935.4705	9486211.3689
	BEST	**1881.7308**	2963.1522	17410.9476	70190.2883	2912529.4497
	STD	**2488.4476**	3397210.3615	21780.1504	99834371.4392	7625090.7050
F14	WORST	**1653.3011**	1186498.6957	1655418.3676	1773865.8031	7892649.6700

	AVERAGE	**1576.4108**	646653.8323	274053.6361	428541.8600	2653158.6347
	BEST	**1530.5724**	149012.4651	9701.7349	66804.2167	431043.8654
	STD	**31.7176**	274498.9123	413231.3400	430925.1789	1848307.9429
F15	WORST	**2417.2182**	1943938.5892	27128.0181	23031112.0001	848022.8190
	AVERAGE	**1955.8472**	225246.8548	13994.9924	5877693.2831	485170.0594
	BEST	**1624.9900**	2943.6671	2498.3600	22774.9968	227782.4947
	STD	**194.7719**	482929.2636	7764.0556	7356137.2720	177920.6769
F16	WORST	3710.7154	4592.3427	4438.2704	**3525.1813**	5803.3860
	AVERAGE	3039.5539	3618.7916	3582.8471	**2902.5948**	5461.5869
	BEST	2420.9349	2711.3435	2800.9628	**2370.1324**	4968.3543
	STD	302.0266	535.5853	456.8734	352.9859	**208.4959**
F17	WORST	3080.7350	3299.7806	4087.7622	**3054.8805**	4509.0647
	AVERAGE	2777.2827	2787.9559	3456.2627	**2590.2032**	4168.4980
	BEST	2386.9796	2450.5866	2892.2004	**2128.5445**	3605.5149
	STD	**184.3244**	201.1050	301.3372	266.9205	214.5706
F18	WORST	**68008.1830**	5803384.3885	427546.7264	6711292.3523	81556914.0223
	AVERAGE	**33245.6856**	2736637.7438	234512.6965	2147793.2880	41585252.6341
	BEST	**14261.2839**	1020141.5365	66853.3640	154053.5460	6812617.2183
	STD	**16319.8771**	1463962.4900	103709.4889	1700898.8177	22392361.8821
F19	WORST	**2681.6258**	15864.8916	44543.3323	99644506.4445	428103.9806
	AVERAGE	**2154.7850**	**8603.8377**	18876.4095	5535469.8611	282197.9220
	BEST	**1976.8392**	4424.7155	5184.6738	127697.5696	111829.0122
	STD	**187.2113**	3122.4493	11523.2381	22156091.0669	80401.6411
F20	WORST	3063.6312	**2983.1790**	4067.4460	3209.1226	4476.5701
	AVERAGE	2777.1247	2719.9998	3445.8669	**2702.6666**	4154.8426
	BEST	2393.5808	2503.3890	3051.9019	**2287.7058**	3761.6049
	STD	208.0133	142.8749	314.8358	217.2997	195.6939

Table S25. Experiments on CS, DE, GSA, GWO, HS for F21~F30 functions under D=50 on parameters II

Problems	Criteria	CS	DE	GSA	GWO	HS
F21	WORST	2595.5734	2687.2439	2734.0048	**2550.7493**	2814.9787
	AVERAGE	2543.0511	2633.4501	2595.5316	**2494.2599**	2778.6347
	BEST	2466.0074	2578.9341	2490.6946	**2451.5708**	2732.7213
	STD	37.6763	33.4743	66.8247	28.2386	**18.8129**
F22	WORST	9619.8435	15179.0363	10283.8217	**8869.4353**	17037.6376
	AVERAGE	9118.3631	14213.5408	8926.6873	**8086.5154**	16457.9219
	BEST	8414.9297	12930.7286	7510.9214	6936.4017	15518.4557
	STD	370.8804	514.9083	696.7020	514.4964	352.5750
F23	WORST	3039.5937	3088.0510	3822.1653	**2977.8101**	3292.7711
	AVERAGE	2970.8573	3035.2232	3318.6493	**2928.9920**	3248.7304
	BEST	2897.9147	2958.0114	2987.0162	**2860.0315**	3199.6836
	STD	41.1864	36.9995	198.2687	35.6962	**28.7382**

F24	WORST	**3268.5611**	3383.0084	3493.8157	3369.3369	3432.0372
	AVERAGE	3174.6077	3318.5480	3302.6392	**3119.0259**	3398.1812
	BEST	3079.8677	3261.6965	3117.0315	**3002.6072**	3357.7378
	STD	46.7549	39.5512	79.9521	103.6092	**22.0915**
F25	WORST	3028.5296	**2931.3426**	3088.9254	4028.6052	5108.2984
	AVERAGE	2990.9150	**2931.2802**	3032.4856	3423.3596	4520.1769
	BEST	2942.4561	**2931.2513**	2964.2828	3057.3814	3948.9707
	STD	30.1100	**0.0266**	39.7740	203.4529	286.1856
F26	WORST	7264.4260	**7141.9486**	9745.2836	7437.6028	9756.6739
	AVERAGE	6139.0968	6354.9705	**5539.6906**	6048.5914	9099.7192
	BEST	2900.0009	5725.8034	**2900.0001**	5183.9332	8722.1477
	STD	1197.4310	413.9506	2839.7968	562.5225	**282.3465**
F27	WORST	3323.9668	**3200.0119**	4460.9491	3715.5984	4030.2077
	AVERAGE	3257.8181	**3200.0116**	3919.7399	3538.1554	3919.5674
	BEST	3219.4419	**3200.0113**	3560.9191	3372.2664	3812.1263
	STD	28.6174	**0.0001**	214.9326	79.2397	65.7020
F28	WORST	3304.4025	**3300.0118**	3883.8606	5565.3802	6186.7374
	AVERAGE	3294.9490	**3300.0117**	3335.7300	4101.5299	5275.7331
	BEST	3257.3531	**3300.0113**	3261.9001	3545.0578	4367.7996
	STD	16.0938	**0.0001**	131.8416	492.2318	480.3973
F29	WORST	**4418.4208**	5468.6628	5420.2673	4862.4720	6364.7579
	AVERAGE	**4056.7505**	4615.2234	4926.3088	4320.5553	5729.5187
	BEST	**3644.1795**	4020.0167	4276.1500	3788.7691	4880.4469
	STD	**172.0778**	478.2961	344.0108	301.2080	332.1567
F30	WORST	**1.89E+06**	2.39E+07	3.53E+06	1.49E+08	4.48E+07
	AVERAGE	**1.03E+06**	6.57E+06	2.38E+06	7.63E+07	2.48E+07
	BEST	7.03E+05	**8.91E+04**	1.37E+06	3.70E+07	1.42E+07
	STD	**3.13E+05**	7.35E+06	5.06E+05	3.29E+07	8.15E+06

Supplementary Material F

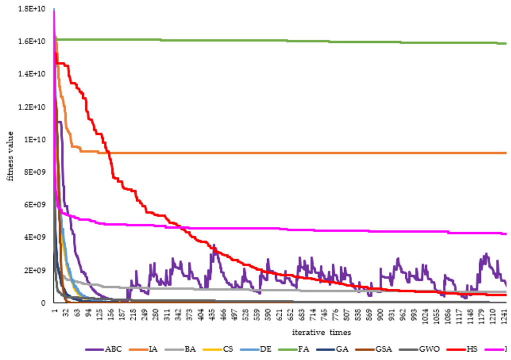

Figure S1. The fitness change curves on F1 under D=10 for parameters I of compared NIOAs

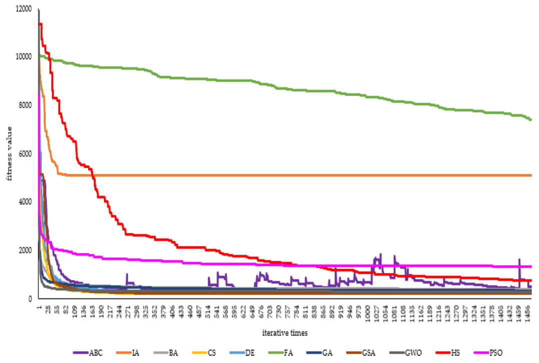

Figure S2. The fitness change curves on F2 under D=10 for parameters I of compared NIOAs

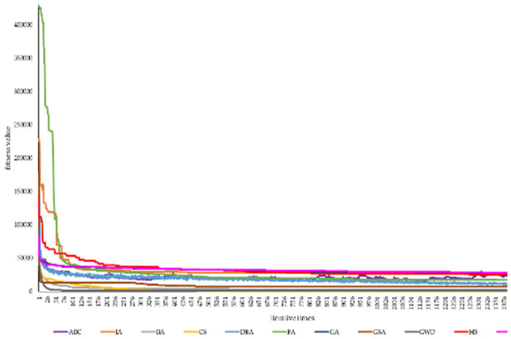

Figure S3. The fitness change curves on F3 under D=10 for parameters I of compared NIOAs

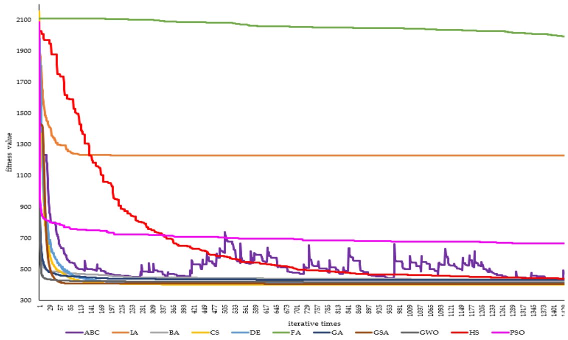

Figure S4. The fitness change curves on F4 under D=10 for parameters I of compared NIOAs

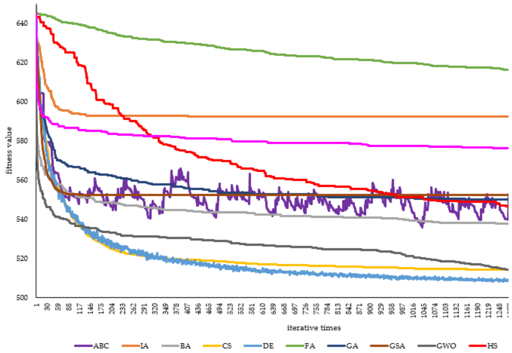

Figure S5. The fitness change curves on F5 under D=10 for parameters I of compared NIOAs

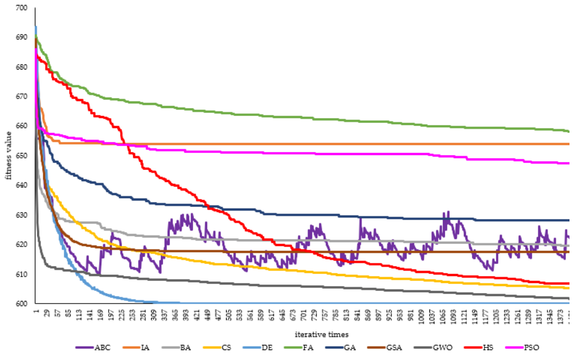

Figure S6. The fitness change curves on F6 under D=10 for parameters I of compared NIOAs

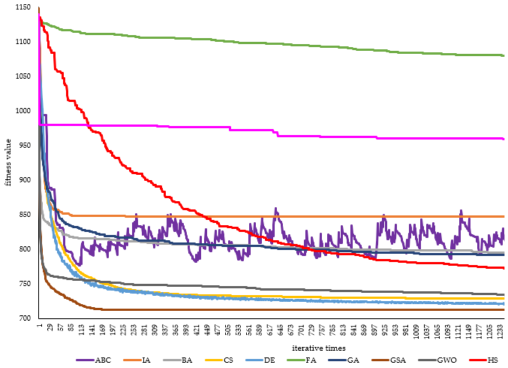

Figure S7. The fitness change curves on F7 under D=10 for parameters I of compared NIOAs

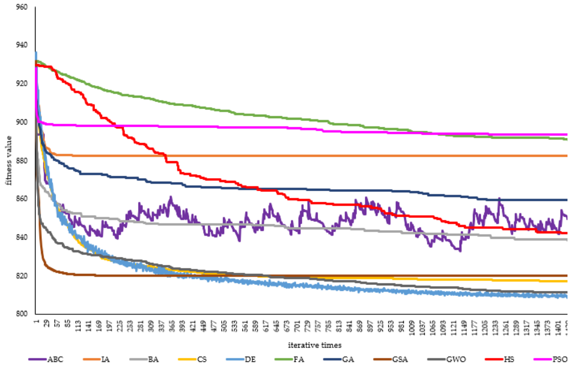

Figure S8. The fitness change curves on F8 under D=10 for parameters I of compared NIOAs

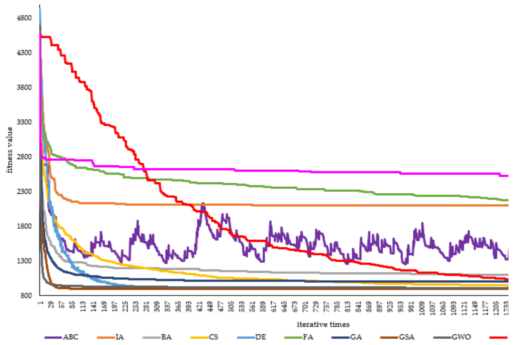

Figure S9. The fitness change curves on F9 under D=10 for parameters I of compared NIOAs

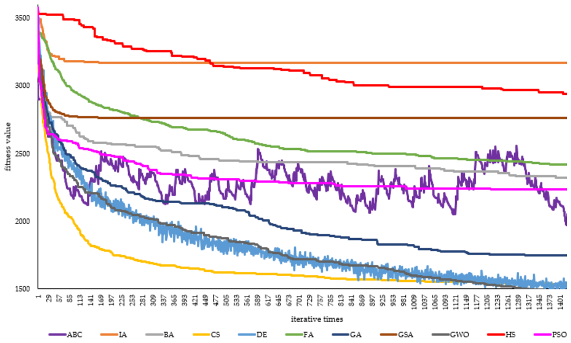

Figure S10. The fitness change curves on F10 under D=10 for parameters I of compared NIOAs

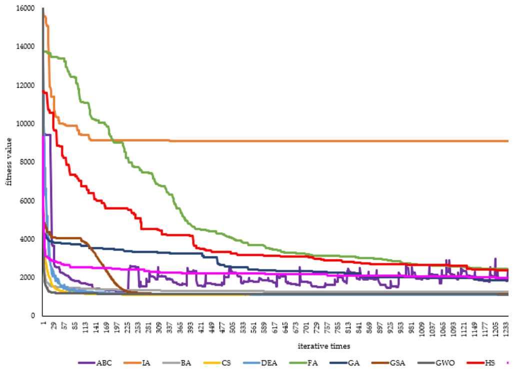

Figure S11. The fitness change curves on F11 under D=10 for parameters I of compared NIOAs

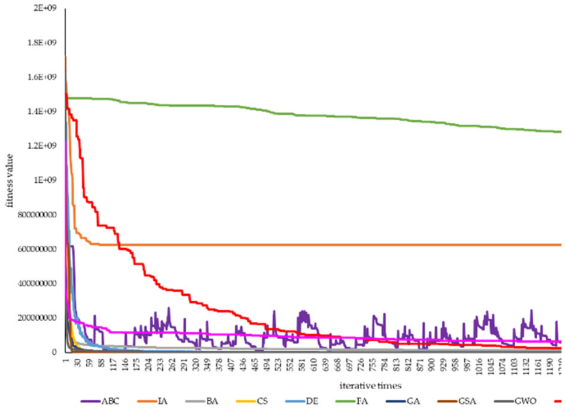

Figure S12. The fitness change curves on F12 under D=10 for parameters I of compared NIOAs

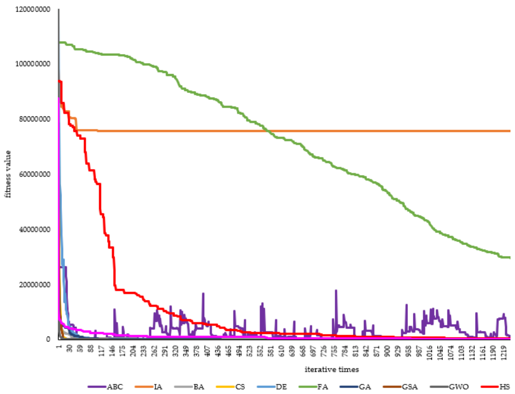

Figure S13. The fitness change curves on F13 under D=10 for parameters I of compared NIOAs

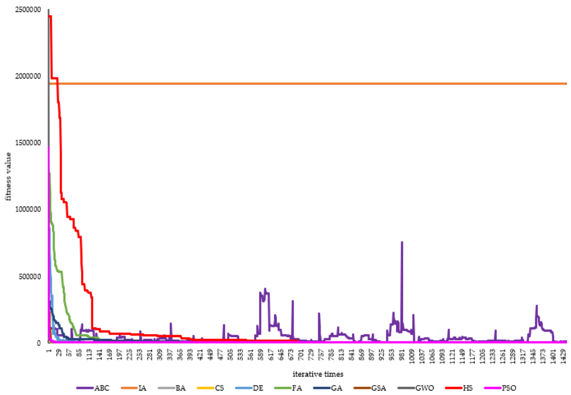

Figure S14. The fitness change curves on F14 under D=10 for parameters I of compared NIOAs

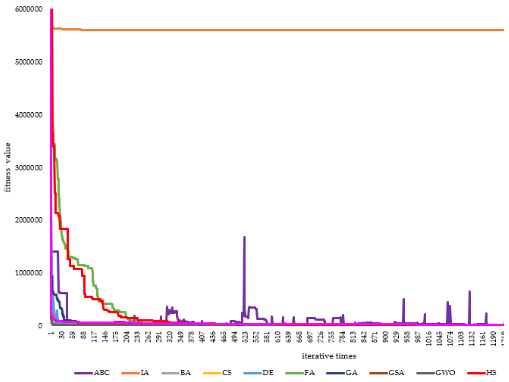

Figure S15. The fitness change curves on F15 under D=10 for parameters I of compared NIOAs

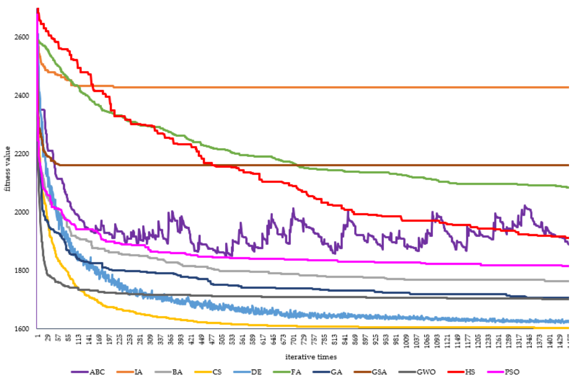

Figure S16. The fitness change curves on F16 under D=10 for parameters I of compared NIOAs

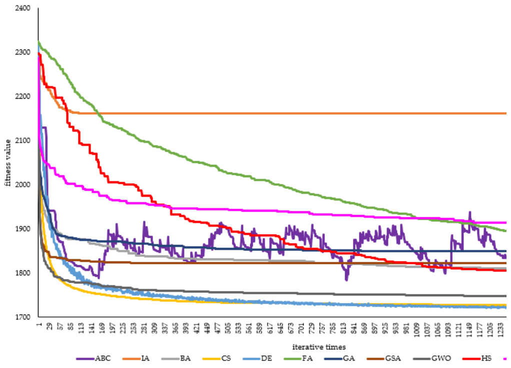

Figure S17. The fitness change curves on F17 under D=10 for parameters I of compared NIOAs

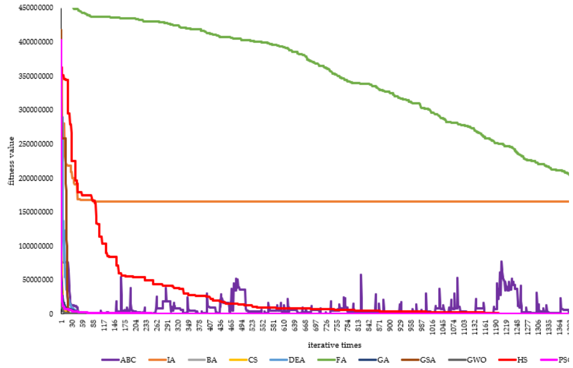

Figure S18. The fitness change curves on F18 under D=10 for parameters I of compared NIOAs

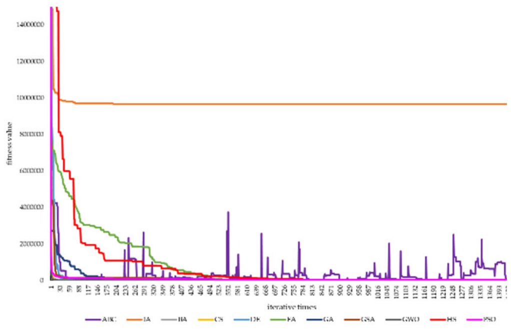

Figure S19. The fitness change curves on F19 under D=10 for parameters I of compared NIOAs

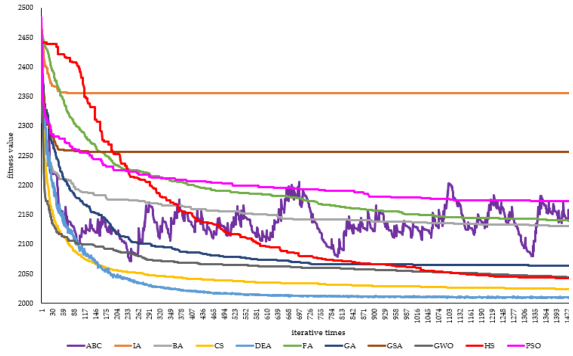

Figure S20. The fitness change curves on F20 under D=10 for parameters I of compared NIOAs

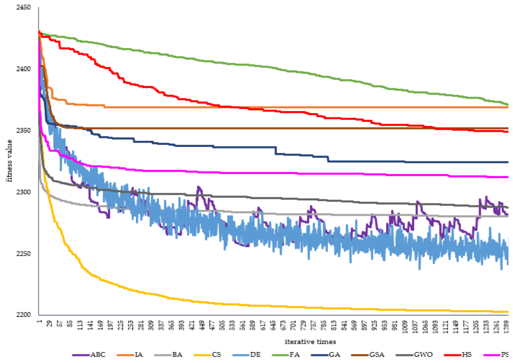

Figure S21. The fitness change curves on F21 under D=10 for parameters I of compared NIOAs

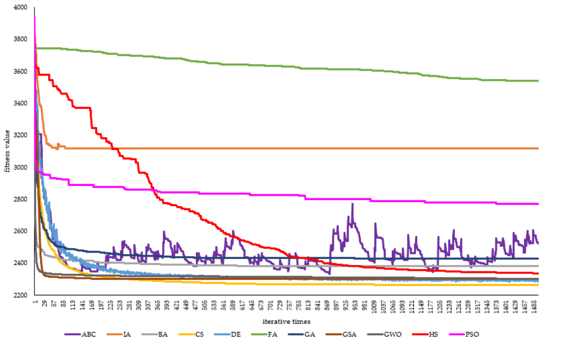

Figure S22. The fitness change curves on F22 under D=10 for parameters I of compared NIOAs

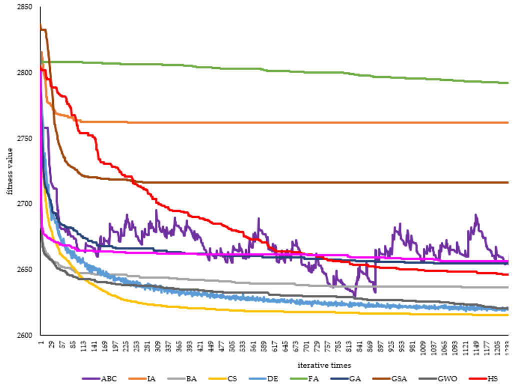

Figure S23. The fitness change curves on F23 under D=10 for parameters I of compared NIOAs

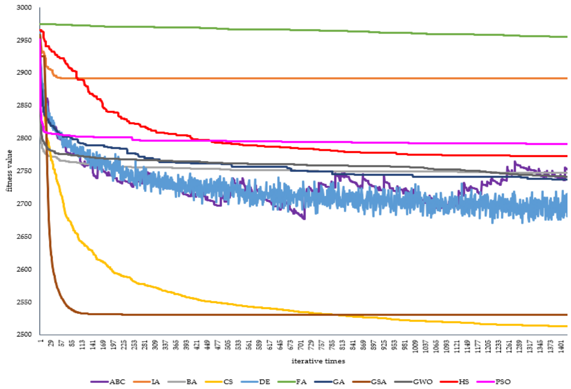

Figure S24. The fitness change curves on F24 under D=10 for parameters I of compared NIOAs

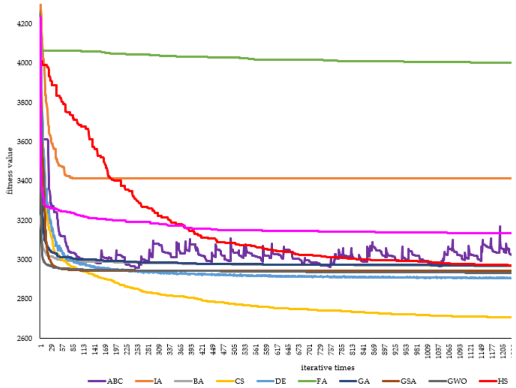

Figure S25. The fitness change curves on F25 under D=10 for parameters I of compared NIOAs

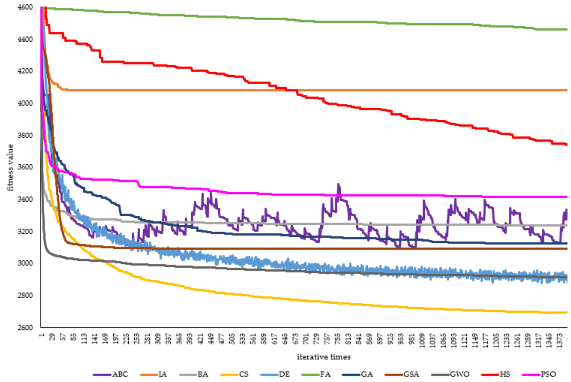

Figure S26. The fitness change curves on F26 under D=10 for parameters I of compared NIOAs

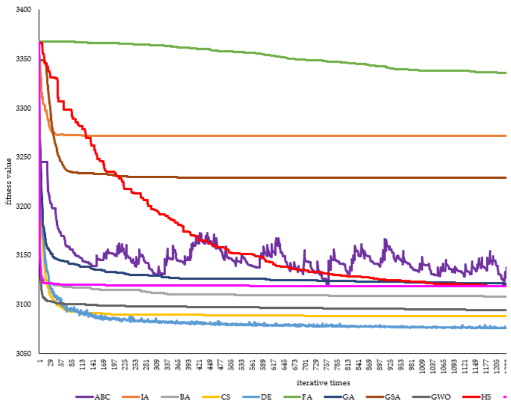

Figure S27. The fitness change curves on F27 under D=10 for parameters I of compared NIOAs

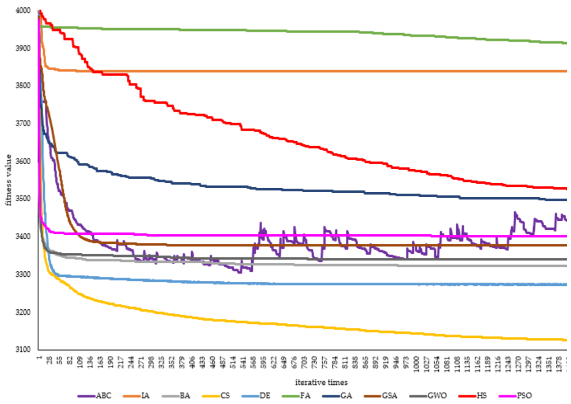

Figure S28. The fitness change curves on F28 under D=10 for parameters I of compared NIOAs

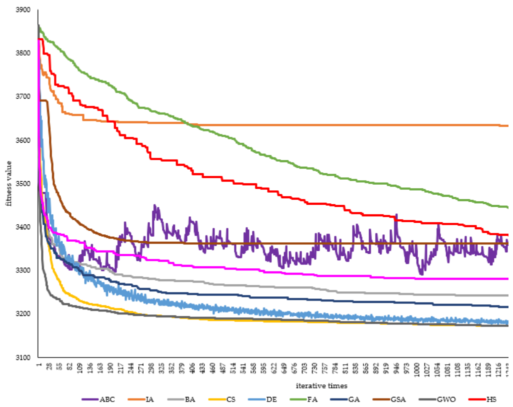

Figure S29. The fitness change curves on F29 under D=10 for parameters I of compared NIOAs

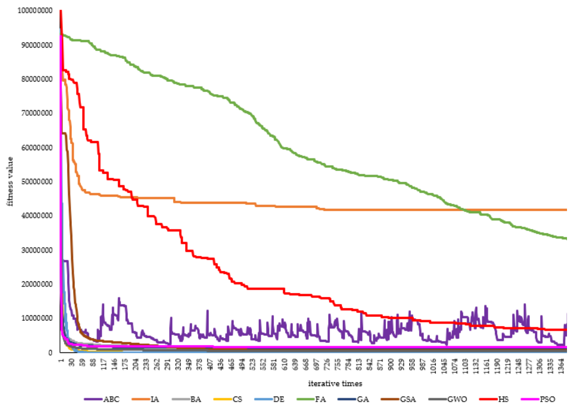

Figure S30. The fitness change curves on F30 under D=10 for parameters I of compared NIOAs

Supplementary Material G

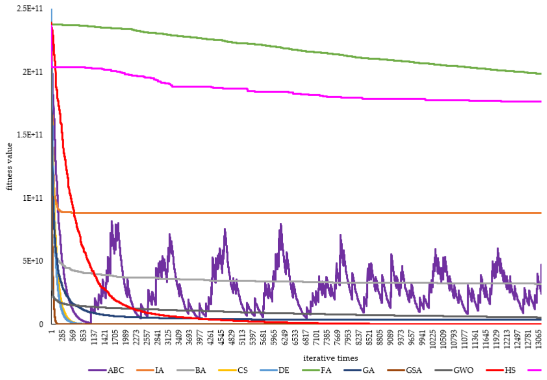

Figure S31. The fitness change curves on F1 under D=50 for parameters I of compared NIOAs

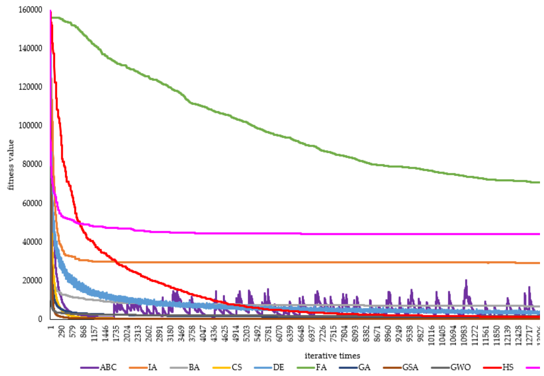

Figure S32. The fitness change curves on F2 under D=50 for parameters I of compared NIOAs

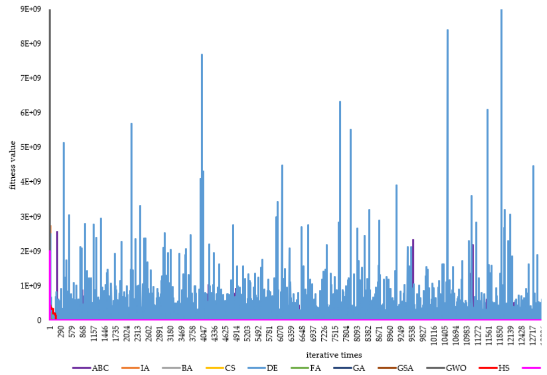

Figure S33. The fitness change curves on F3 under D=50 for parameters I of compared NIOAs

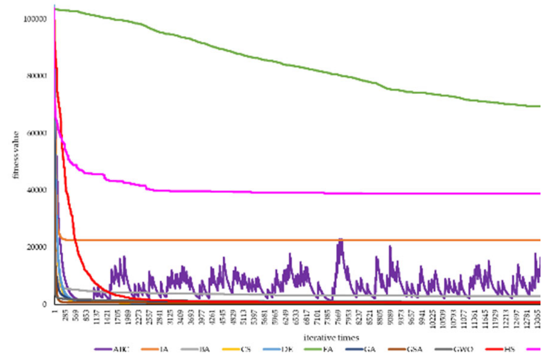

Figure S34. The fitness change curves on F4 under D=50 for parameters I of compared NIOAs

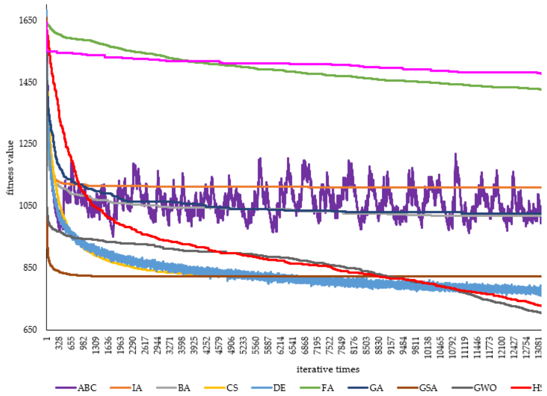

Figure S35. The fitness change curves on F5 under D=50 for parameters I of compared NIOAs

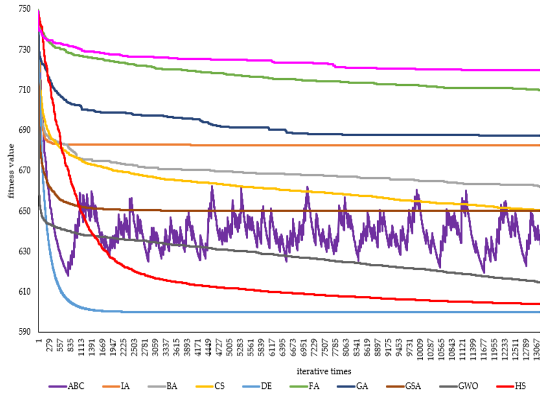

Figure S36. The fitness change curves on F6 under D=50 for parameters I of compared NIOAs

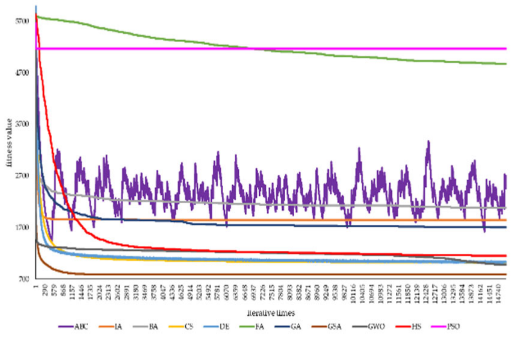

Figure S37. The fitness change curves on F7 under D=50 for parameters I of compared NIOAs

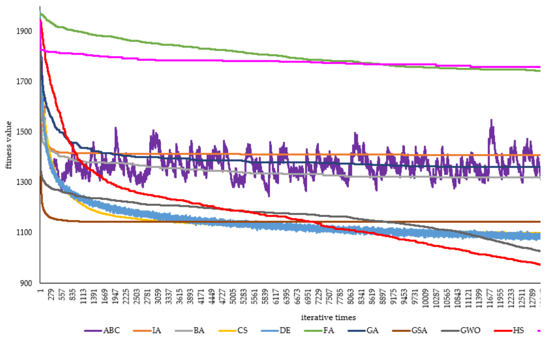

Figure S38. The fitness change curves on F8 under D=50 for parameters I of compared NIOAs

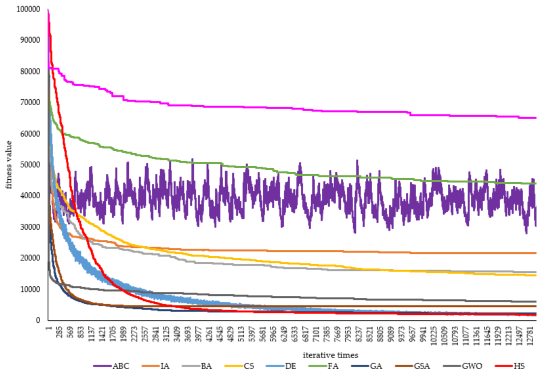

Figure S39. The fitness change curves on F9 under D=50 for parameters I of compared NIOAs

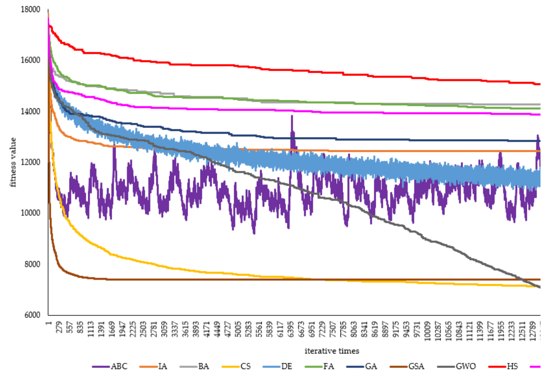

Figure S40. The fitness change curves on F10 under D=50 for parameters I of compared NIOAs

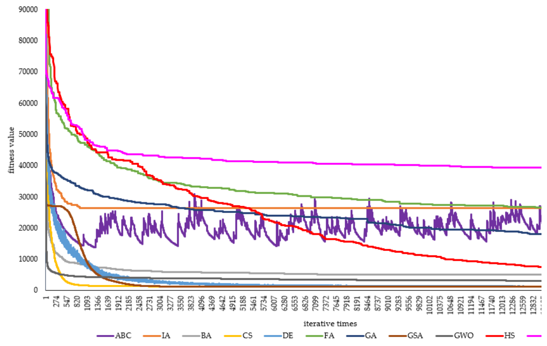

Figure S41. The fitness change curves on F11 under D=50 for parameters I of compared NIOAs

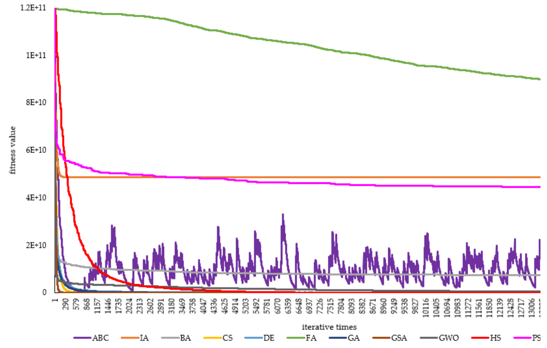

Figure S42. The fitness change curves on F12 under D=50 for parameters I of compared NIOAs

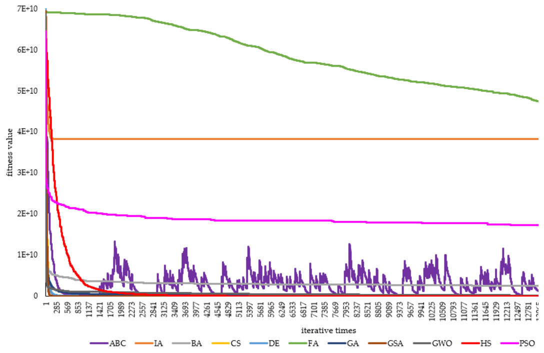

Figure S43. The fitness change curves on F13 under D=50 for parameters I of compared NIOAs

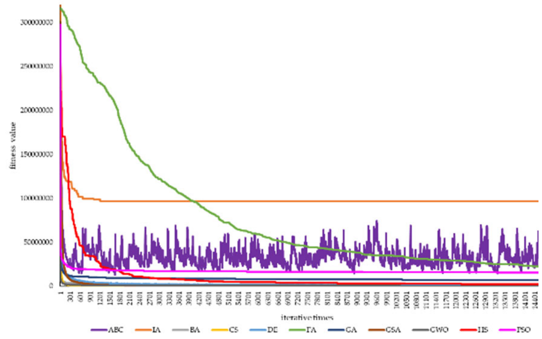

Figure S44. The fitness change curves on F14 under D=50 for parameters I of compared NIOAs

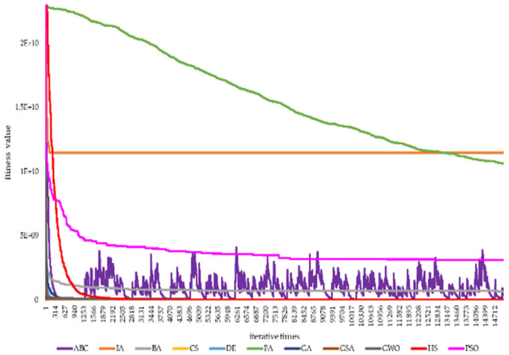

Figure S45. The fitness change curves on F15 under D=50 for parameters I of compared NIOAs

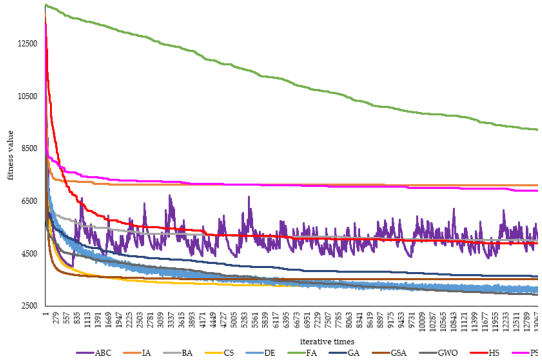

Figure S46. The fitness change curves on F16 under D=50 for parameters I of compared NIOAs

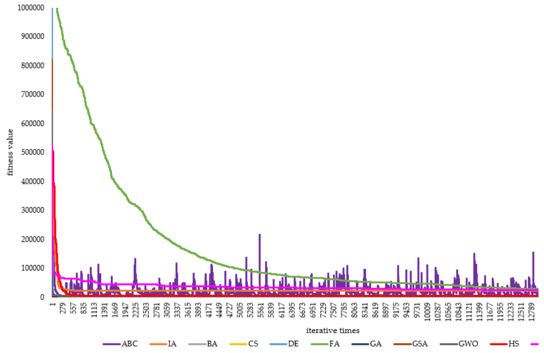

Figure S47. The fitness change curves on F17 under D=50 for parameters I of compared NIOAs

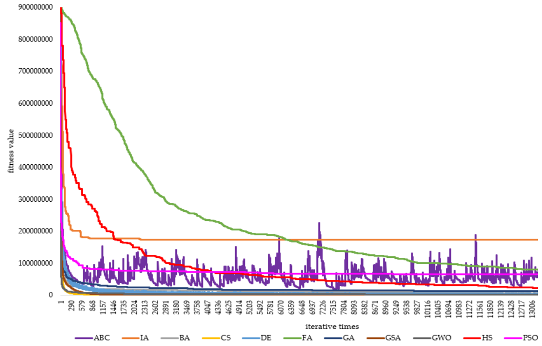

Figure S48. The fitness change curves on F18 under D=50 for parameters I of compared NIOAs

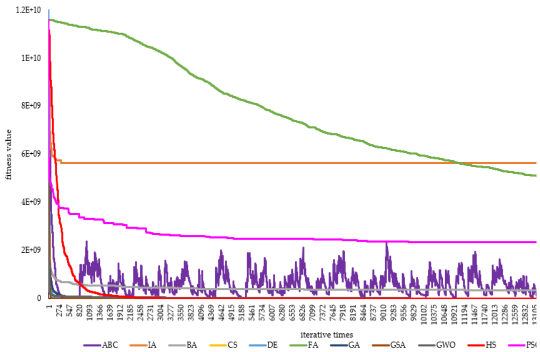

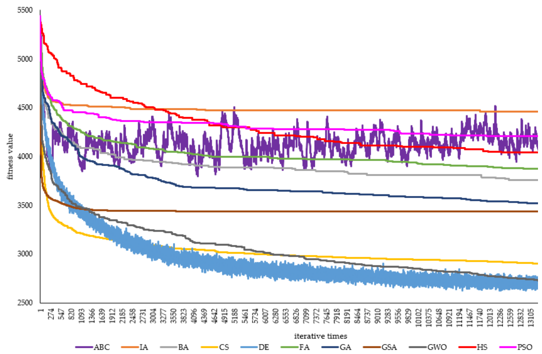

Figure S49. The fitness change curves on F19 under D=50 for parameters I of compared NIOAs

Figure S50. The fitness change curves on F20 under D=50 for parameters I of compared NIOAs

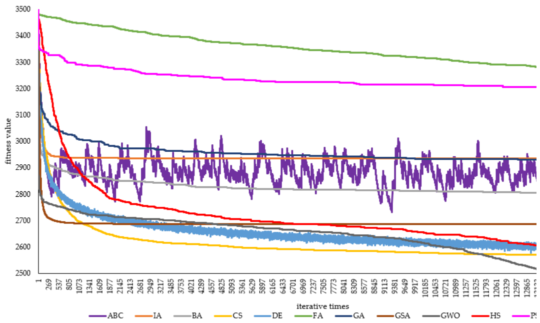

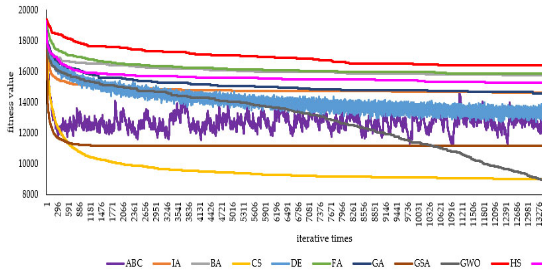

Figure S51. The fitness change curves on F21 under D=50 for parameters I of compared NIOAs

Figure S52. The fitness change curves on F22 under D=50 for parameters I of compared NIOAs

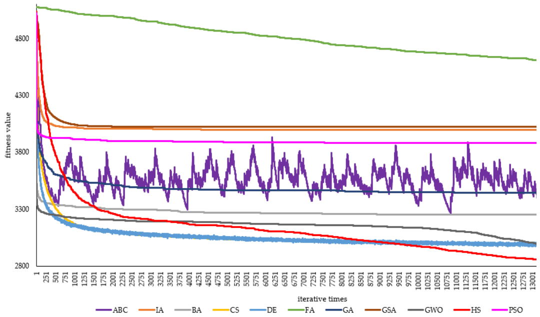

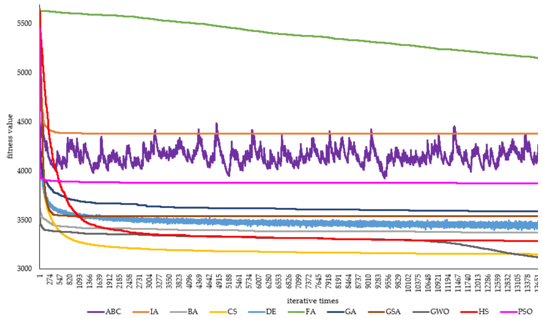

Figure S53. The fitness change curves on F23 under D=50 for parameters I of compared NIOAs

Figure S54. The fitness change curves on F24 under D=50 for parameters I of compared NIOAs

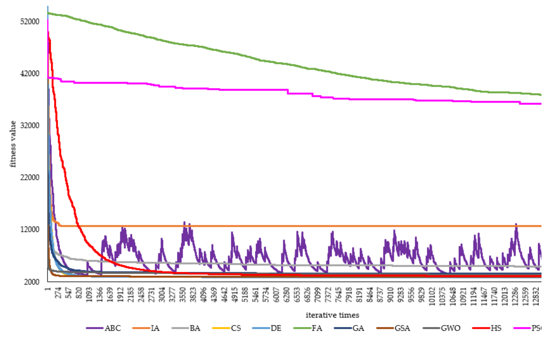

Figure S55. The fitness change curves on F25 under D=50 for parameters I of compared NIOAs

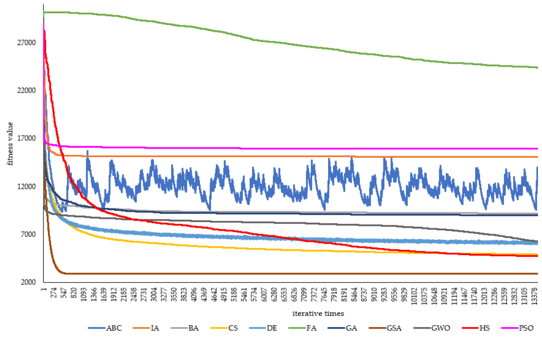

Figure S56. The fitness change curves on F26 under D=50 for parameters I of compared NIOAs

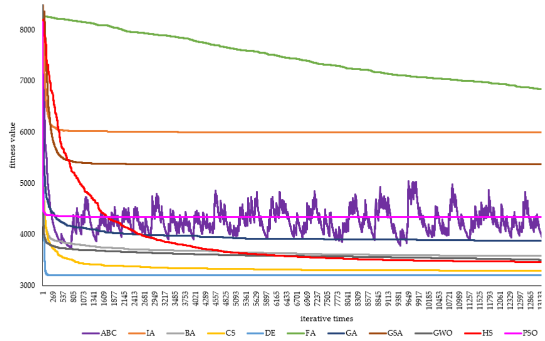

Figure S57. The fitness change curves on F27 under D=50 for parameters I of compared NIOAs

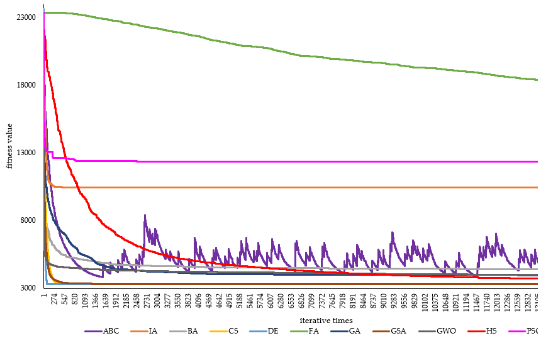

Figure S58. The fitness change curves on F28 under D=50 for parameters I of compared NIOAs

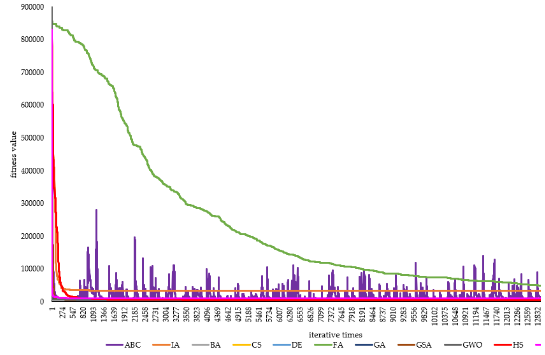

Figure S59. The fitness change curves on F29 under D=50 for parameters I of compared NIOAs

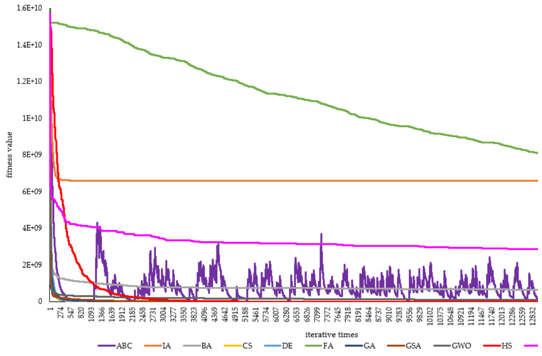

Figure S60. The fitness change curves on F30 under D=50 for parameters I of compared NIOAs

Supplementary Material H

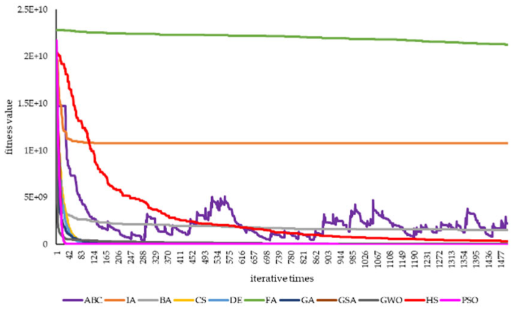

Figure S61. The fitness change curves on F1 under D=10 for parameters II of compared NIOAs

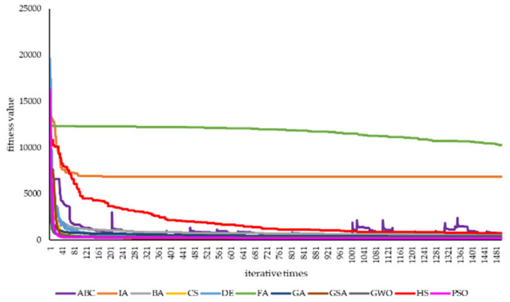

Figure S62. The fitness change curves on F2 under D=10 for parameters II of compared NIOAs

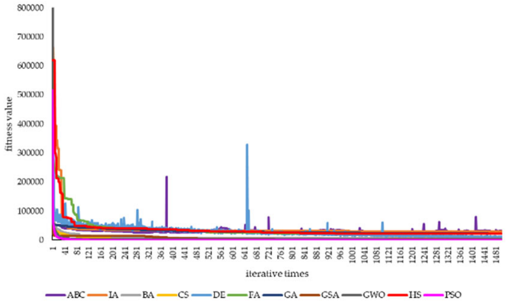

Figure S63. The fitness change curves on F3 under D=10 for parameters II of compared NIOAs

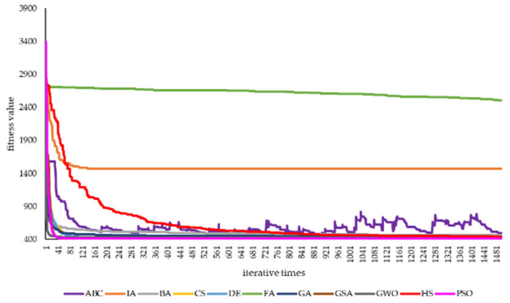

Figure S64. The fitness change curves on F4 under D=10 for parameters II of compared NIOAs

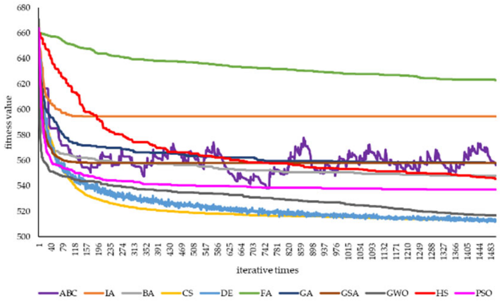

Figure S65. The fitness change curves on F5 under D=10 for parameters II of compared NIOAs

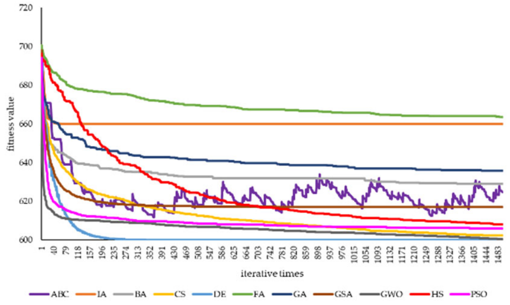

Figure S66. The fitness change curves on F6 under D=10 for parameters II of compared NIOAs

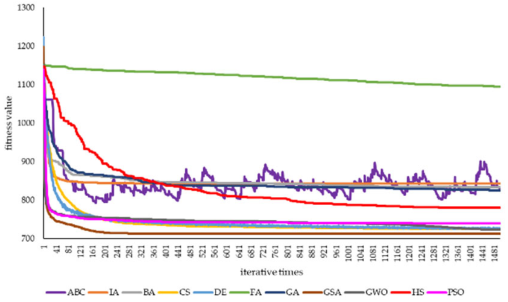

Figure S67. The fitness change curves on F7 under D=10 for parameters II of compared NIOAs

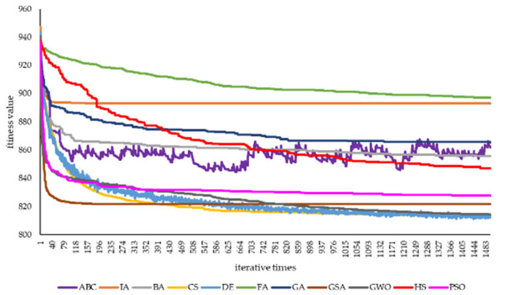

Figure S68. The fitness change curves on F8 under D=10 for parameters II of compared NIOAs

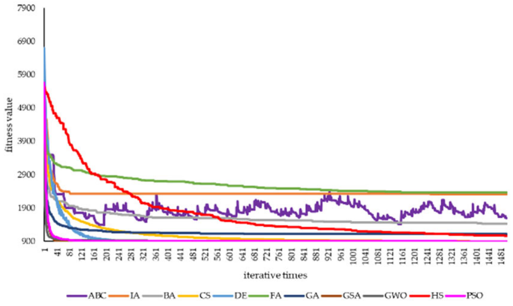

Figure S69. The fitness change curves on F9 under D=10 for parameters II of compared NIOAs

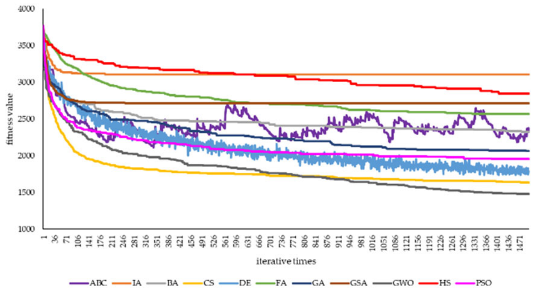

Figure S70. The fitness change curves on F10 under D=10 for parameters II of compared NIOAs

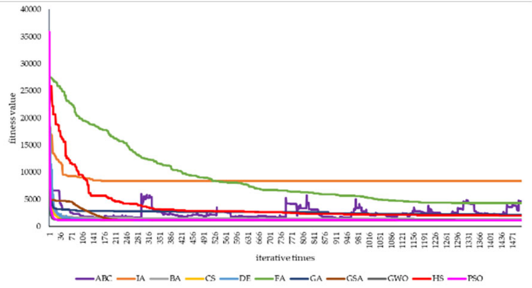

Figure S71. The fitness change curves on F11 under D=10 for parameters II of compared NIOAs

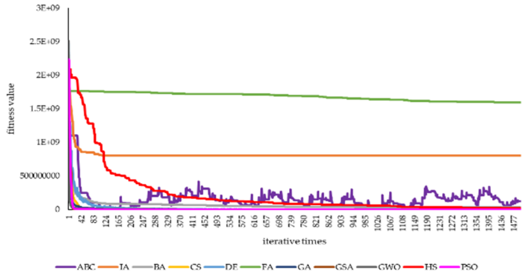

Figure S72. The fitness change curves on F12 under D=10 for parameters II of compared NIOAs

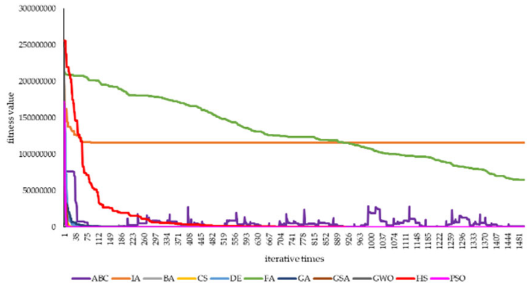

Figure S73. The fitness change curves on F13 under D=10 for parameters II of compared NIOAs

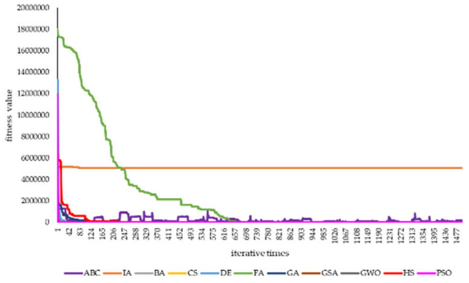

Figure S74. The fitness change curves on F14 under D=10 for parameters II of compared NIOAs

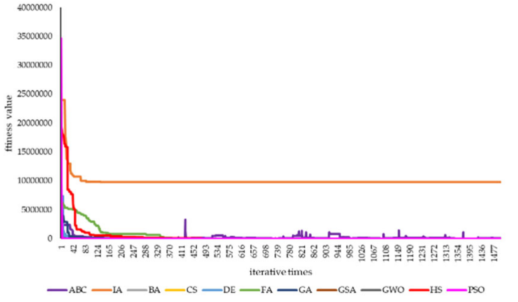

Figure S75. The fitness change curves on F15 under D=10 for parameters II of compared NIOAs

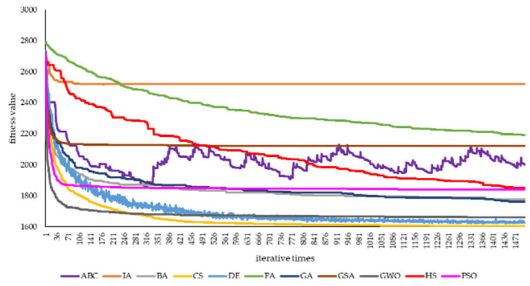

Figure S76. The fitness change curves on F16 under D=10 for parameters II of compared NIOAs

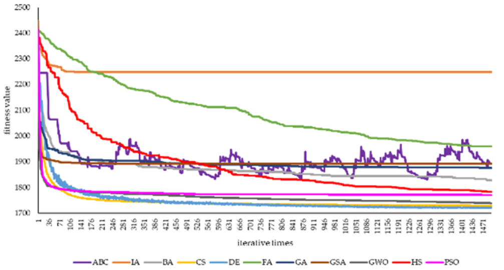

Figure S77. The fitness change curves on F17 under D=10 for parameters II of compared NIOAs

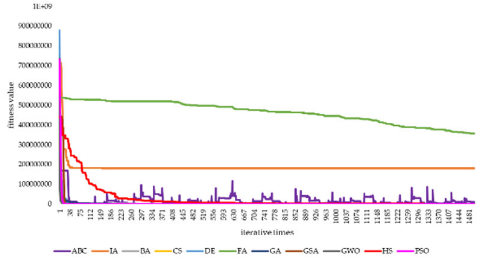

Figure S78. The fitness change curves on F18 under D=10 for parameters II of compared NIOAs

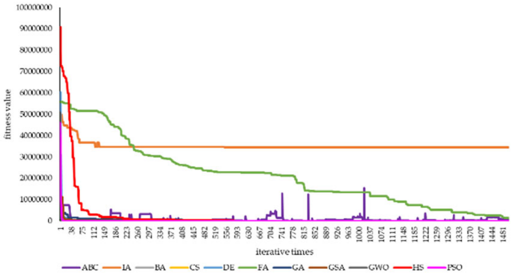

Figure S79. The fitness change curves on F19 under D=10 for parameters II of compared NIOAs

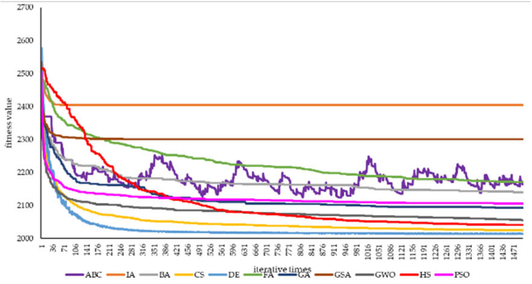

Figure S80. The fitness change curves on F20 under D=10 for parameters II of compared NIOAs

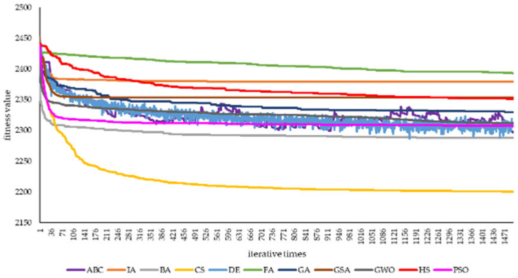

Figure S81. The fitness change curves on F21 under D=10 for parameters II of compared NIOAs

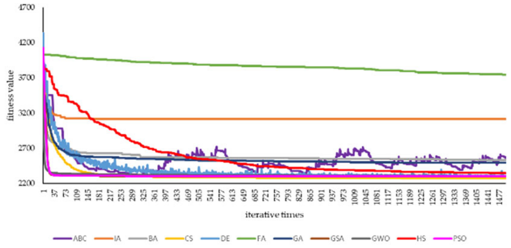

Figure S82. The fitness change curves on F22 under D=10 for parameters II of compared NIOAs

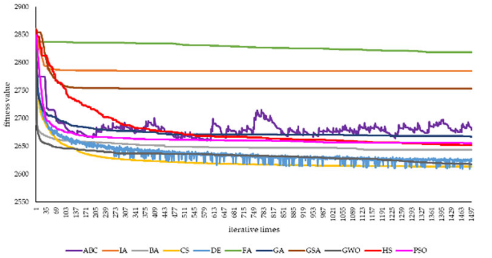

Figure S83. The fitness change curves on F23 under D=10 for parameters II of compared NIOAs

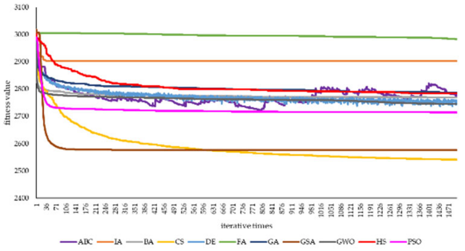

Figure S84. The fitness change curves on F24 under D=10 for parameters II of compared NIOAs

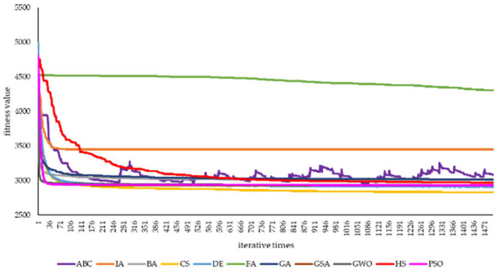

Figure S85. The fitness change curves on F25 under D=10 for parameters II of compared NIOAs

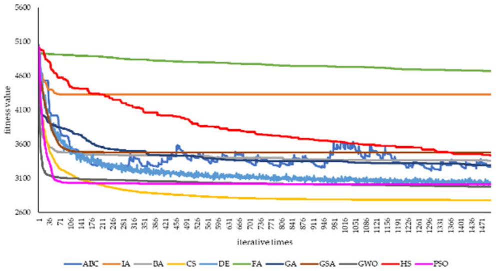

Figure S86. The fitness change curves on F26 under D=10 for parameters II of compared NIOAs

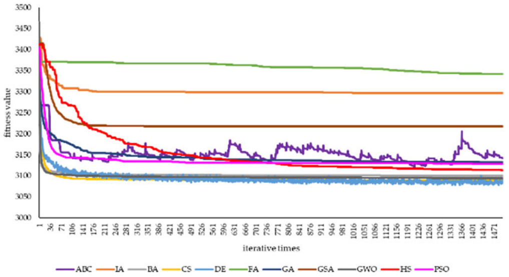

Figure S87. The fitness change curves on F27 under D=10 for parameters II of compared NIOAs

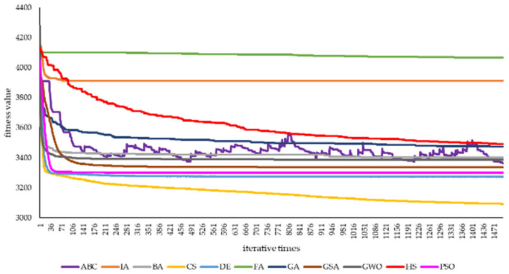

Figure S88. The fitness change curves on F28 under D=10 for parameters II of compared NIOAs

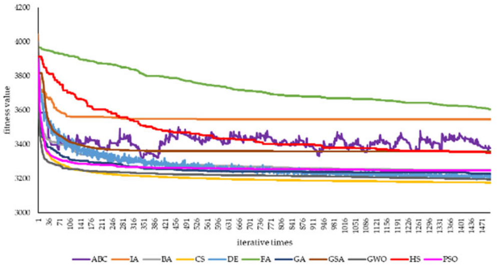

Figure S89. The fitness change curves on F29 under D=10 for parameters II of compared NIOAs

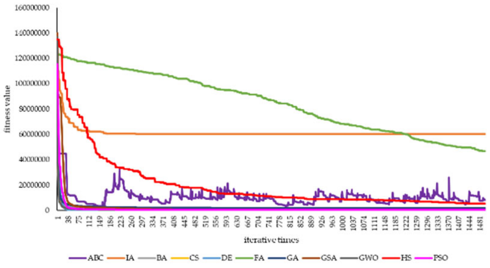

Figure S90. The fitness change curves on F30 under D=10 for parameters II of compared NIOAs

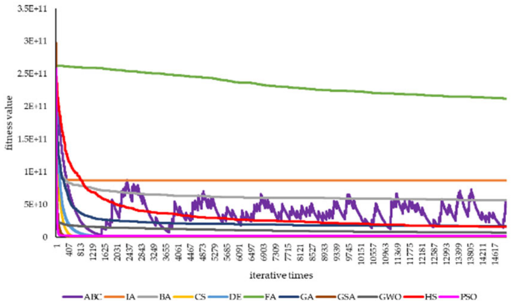

Figure S91. The fitness change curves on F1 under D=50 for parameters II of NIOAs

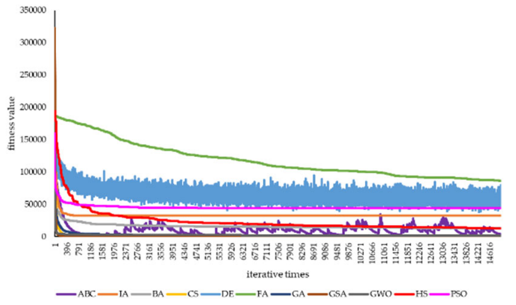

Figure S92. The fitness change curves on F2 under D=50 for parameters II of NIOAs

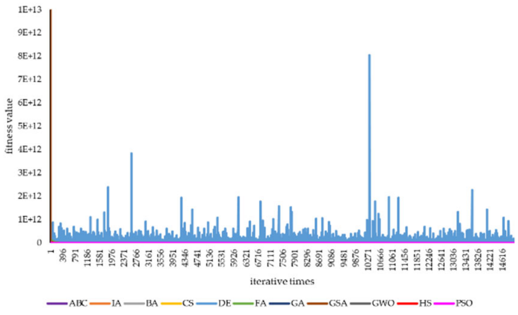

Figure S93. The fitness change curves on F3 under D=50 for parameters II of compared NIOAs

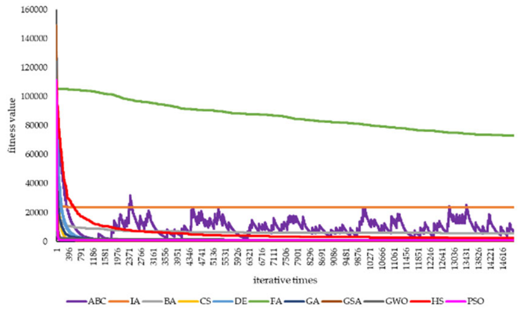

Figure S94. The fitness change curves on F4 under D=50 for parameters II of compared NIOAs

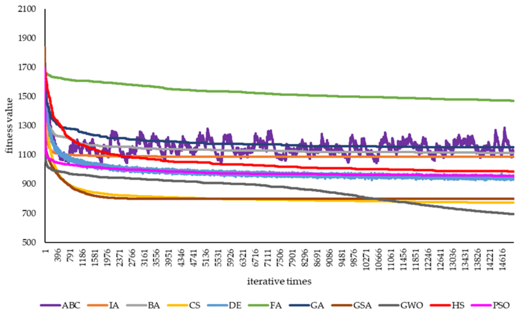

Figure S95. The fitness change curves on F5 under D=50 for parameters II of compared NIOAs

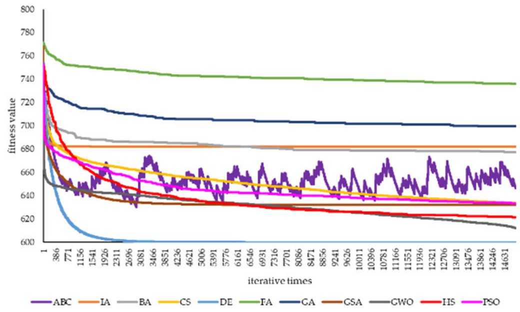

Figure S96. The fitness change curves on F6 under D=50 for parameters II of compared NIOAs

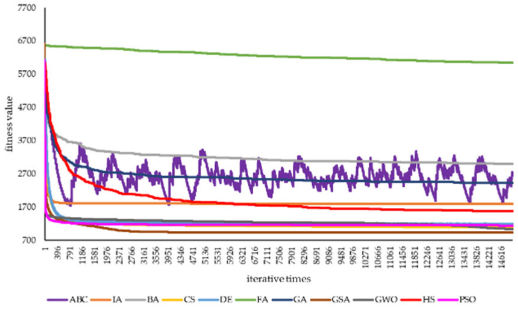

Figure S97. The fitness change curves on F7 under D=50 for parameters II of compared NIOAs

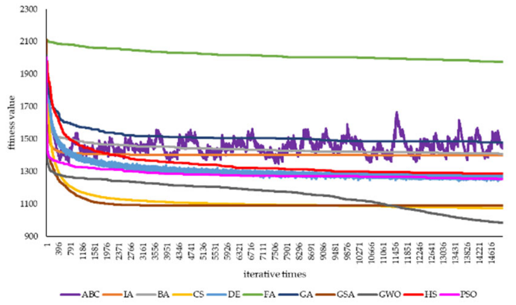

Figure S98. The fitness change curves on F8 under D=50 for parameters II of compared NIOAs

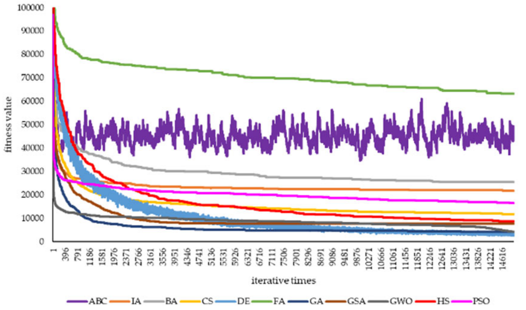

Figure S99. The fitness change curves on F9 under D=50 for parameters II of compared NIOAs

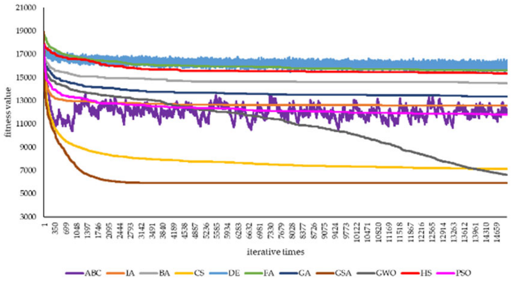

Figure S100. The fitness change curves on F10 under D=50 for parameters II of compared NIOAs

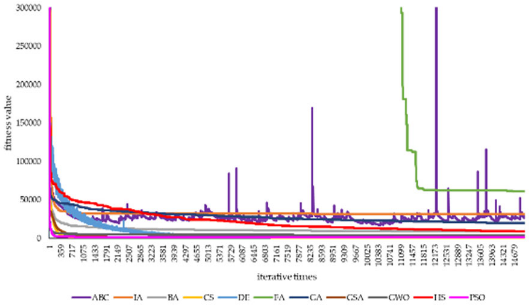

Figure S101. The fitness change curves on F11 under D=50 for parameters II of compared NIOAs

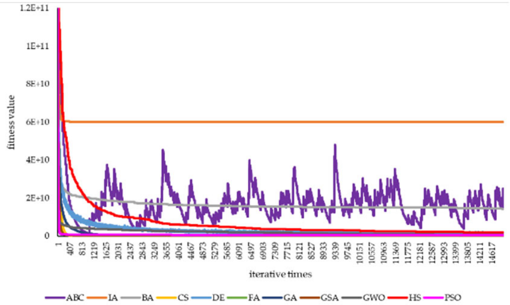

Figure S102. The fitness change curves on F12 under D=50 for parameters II of compared NIOAs

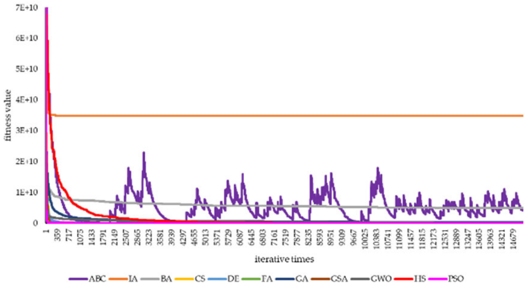

Figure S103. The fitness change curves on F13 under D=50 for parameters II of compared NIOAs

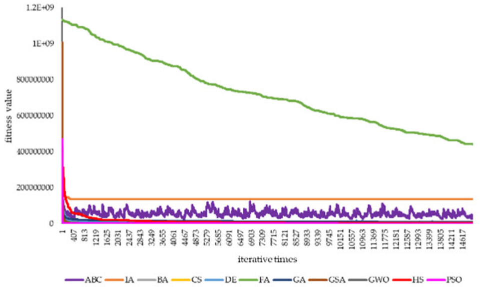

Figure S104. The fitness change curves on F14 under D=50 for parameters II of compared NIOAs

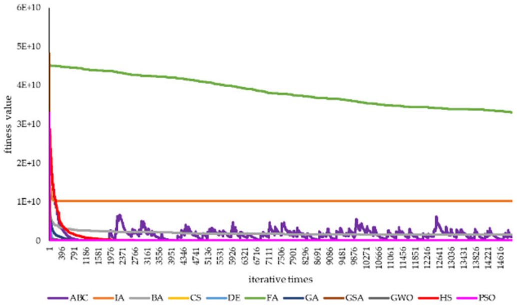

Figure S105. The fitness change curves on F15 under D=50 for parameters II of compared NIOAs

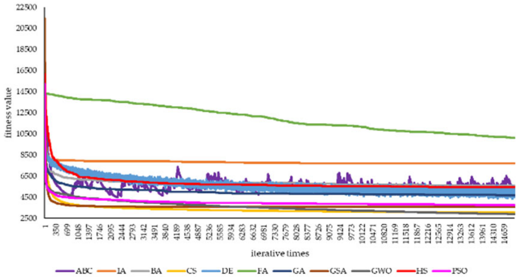

Figure S106. The fitness change curves on F16 under D=50 for parameters II of compared NIOAs

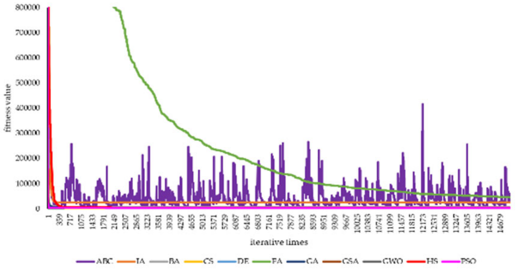

Figure S107. The fitness change curves on F17 under D=50 for parameters II of compared NIOAs

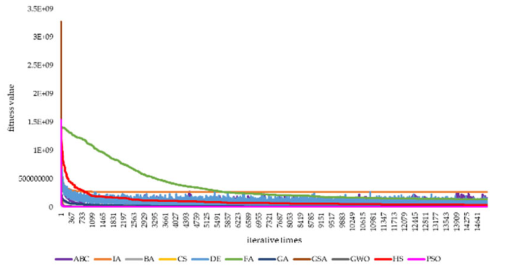

Figure S108. The fitness change curves on F18 under D=50 for parameters II of compared NIOAs

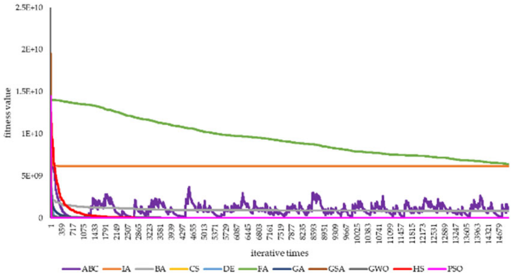

Figure S109. The fitness change curves on F19 under D=50 for parameters II of compared NIOAs

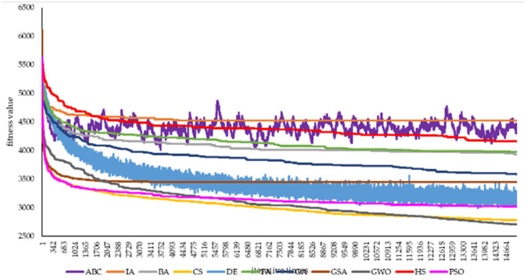

Figure S110. The fitness change curves on F20 under D=50 for parameters II of compared NIOAs

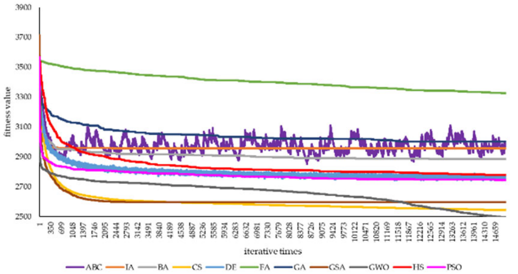

Figure S111. The fitness change curves on F21 under D=50 for parameters II of compared NIOAs

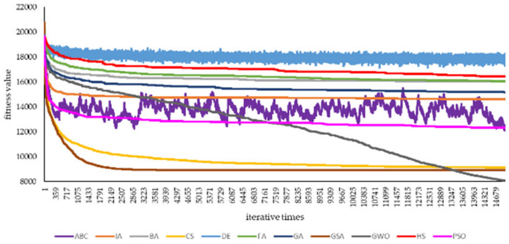

Figure S112. The fitness change curves on F22 under D=50 for parameters II of compared NIOAs

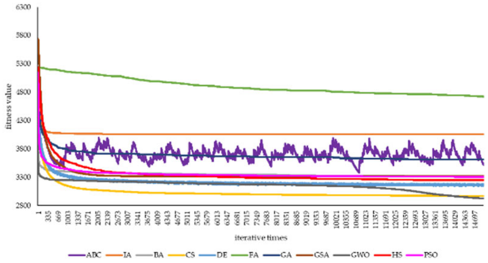

Figure S113. The fitness change curves on F23 under D=50 for parameters II of compared NIOAs

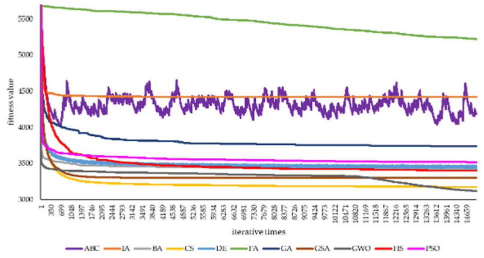

Figure S114. The fitness change curves on F24 under D=50 for parameters II of compared NIOAs

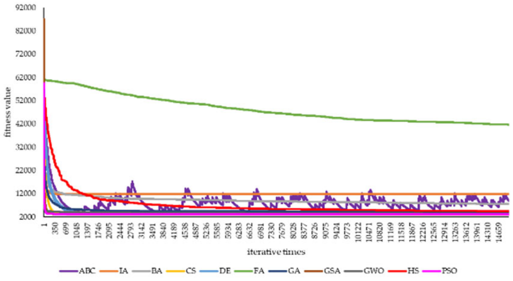

Figure S115. The fitness change curves on F25 under D=50 for parameters II of compared NIOAs

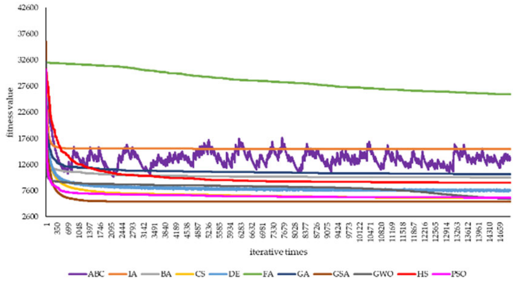

Figure S116. The fitness change curves on F26 under D=50 for parameters II of compared NIOAs

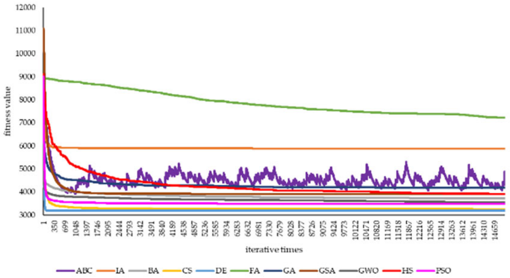

Figure S117. The fitness change curves on F27 under D=50 for parameters II of compared NIOAs

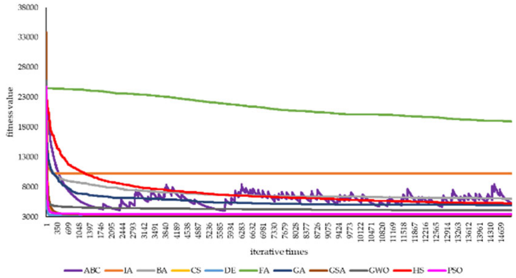

Figure S118. The fitness change curves on F28 under D=50 for parameters II of compared NIOAs

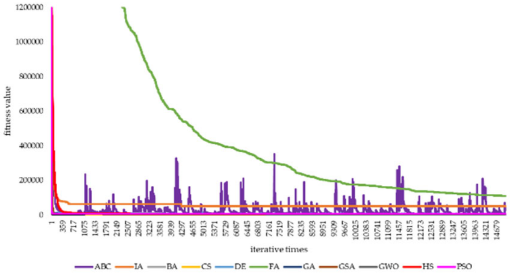

Figure S119. The fitness change curves on F29 under D=50 for parameters II of compared NIOAs

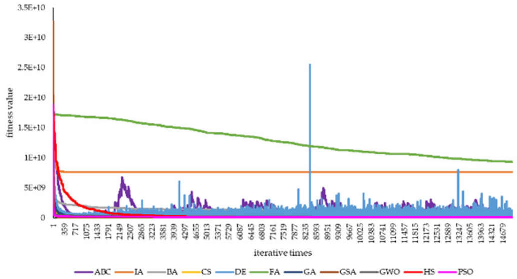

Figure S120. The fitness change curves on F30 under D=50 for parameters II of compared NIOAs

Supplementary Material J

Table S26. The times of obtained the optimal results for 11 compared NIOAs

Criteria / NIOAs		Parameters I		Parameters II	
		**BEST**	**STD**	**BEST**	**STD**
DE	D=10	**28** (F1, F2, F4, F5, F6, F7, F8, F9, F10, F11, F13, F14, F15, F16, F17, F18, F19, F20, F21, F22, F23, F24, F25, F26, F27, F28, F29, F30)	**23** (F2, F4, F5, F6, F7, F8, F9, F10, F11, F15, F16, F17, F19, F20, F21, F22, F23, F24, F25, F26, F27, F28, F29)	**28** (F1, F2, F4, F5, F6, F7, F8, F9, F10, F11, F13, F14, F15, F16, F17, F18, F19, F20, F21, F22, F23, F24, F25, F26, F27, F28, F29, F30)	**23** (F2, F4, F5, F6, F7, F8, F9, F10, F11, F14, F15, F16, F17, F18, F19, F20, F21, F22, F23, F24, F25, F26, F27, F28, F29)
	D=50	**7** (F4, F6, F9, F11, F20, F27, F28)	**12** (F4, F6, F7, F8, F20, F23, F24, F25, F26, F27, F28, F29)	**6** (F4, F6, F9, F11, F27, F28)	**12** (F4, F6, F7, F8, F20, F23, F24, F25, F26, F27, F28, F29)
CS	D=10	**26** (F2, F3, F4, F5, F6, F7, F8, F9, F11, F13, F14, F15, F16, F17, F18, F19, F20, F21, F22, F23, F24, F25, F26, F27, F28, F29)	**26** (F2, F3, F4, F5, F6, F7, F8, F9, F10, F11, F13, F14, F15, F16, F17, F19, F20, F21, F22, F23, F24, F25, F26, F27, F28, F29)	**27** (F1, F2, F3, F4, F5, F6, F7, F8, F9, F11, F13, F14, F15, F16, F17, F18, F19, F20, F21, F22, F23, F24, F25, F26, F27, F28, F29)	**26** (F2, F3, F4, F5, F6, F7, F8, F9, F10, F11, F13, F14, F15, F16, F17, F19, F20, F21, F22, F23, F24, F25, F26, F27, F28, F29)
	D=50	**7** (F1, F2, F4, F6, F11, F19, F28)	**13** (F2, F4, F6, F11, F16, F17, F20, F23, F24, F25, F27, F28, F29)	**6** (F2, F4, F6, F11, F19, F28)	**12** (F2, F4, F6, F11, F17, F20, F23, F24, F25, F27, F28, F29)
HS	D=10	**20** (F4, F5, F6, F7, F8, F9, F14, F15, F16, F17, F19, F20, F21, F22, F23, F24, F25, F27, F28, F29)	**19** (F4, F5, F6, F7, F8, F9, F10, F11, F16, F17, F20, F21, F22, F23, F24, F25, F27, F28, F29)	**19** (F4, F5, F6, F7, F8, F9, F14, F16, F17, F19, F20, F21, F22, F23, F24, F25, F27, F28, F29)	**17** (F4, F5, F6, F7, F8, F9, F16, F17, F20, F21, F22, F23, F24, F25, F27, F28, F29)
	D=50	**1** (F6)	**10** (F4, F6, F8, F20, F23, F24, F25, F26, F27, F28)	**1** (F6)	**7** (F6, F8, F20, F23, F24, F26, F27)
GSA	D=10	**21** (F1, F2, F4, F5, F6, F7, F8, F9, F11, F16, F17, F20, F21, F22, F23, F24, F25, F26, F27, F28, F29)	**20** (F1, F2, F4, F5, F6, F7, F8, F9, F11, F16, F17, F20, F21, F22, F23, F24, F25, F27, F28, F29)	**21** (F1, F2, F4, F5, F6, F7, F8, F9, F11 F16, F17, F20, F21, F22, F23, F24, F25, F26, F27, F28, F29)	**19** (F2, F4, F5, F6, F7, F8, F9, F11, F16, F17, F20, F21, F22, F23, F24, F25, F27, F28, F29)
	D=50	**8** (F1, F2, F4, F6, F7, F11, F26, F28)	**13** (F2, F4, F6, F7, F8, F11, F20, F23, F24, F25, F26, F28, F29)	**7** (F2, F4, F6, F7, F11, F26, F28)	**11** (F2, F4, F6, F7, F8, F11, F23, F24, F25, F28, F29)
GWO	D=10	**20** (F3, F4, F5, F6, F7, F8, F9, F10, F11, F14, F15, F16, F17, F19, F20, F21, F22, F23, F24, F25, F26, F27, F28, F29)	**20** (F4, F5, F6, F7, F8, F9, F10, F11, F16, F17, F20, F21, F22, F23, F24, F25, F26, F27, F28, F29)	**21** (F2, F3, F4, F5, F6, F7, F8, F9, F10, F11, F14, F15, F16, F17, F19, F20, F21, F22, F23, F24, F25, F26, F27, F28, F29)	**21** (F2, F4, F5, F6, F7, F8, F9, F10, F11, F16, F17, F20, F21, F22, F23, F24, F25, F26, F27, F28, F29)
	D=50	**2** (F6, F20)	**9** (F6, F17, F20, F23, F24, F25, F27, F28, F29)	**2** (F6, F20)	**8** (F6, F17, F20, F23, F24, F25, F27, F29)

ABC	D=10	**25** (F2, F4, F5, F6, F7, F8, F9, F10, F11, F13, F14, F15, F16, F17, F19, F20, F21, F22, F23, F24, F25, F26, F27, F28, F29)	**23** (F2, F4, F5, F6, F7, F8, F9, F10, F11, F14, F15, F16, F17, F20, F21, F22, F23, F24, F25, F26, F27, F28, F29)	**25** (F2, F4, F5, F6, F7, F8, F9, F10, F11, F13, F14, F15, F16, F17, F19, F20, F21, F22, F23, F24, F25, F26, F27, F28, F29)	**20** (F4, F5, F6, F7, F8, F9, F10, F11, F16, F17, F20, F21, F22, F23, F24, F25, F26, F27, F28, F29)
	D=50	**1** (F6)	**12** (F4, F6, F7, F8, F17, F20, F23, F24, F25, F27, F28, F29)	**1** (F6)	**12** (F4, F6, F7, F8, F17, F20, F23, F24, F25, F27, F28, F29)
PSO	D=10	**19** (F5, F6, F7, F8, F11, F14, F16, F17, F20, F21, F22, F23, F24, F25, F27, F28, F29)	**15** (F5, F6, F7, F8, F16, F17, F20, F21, F22, F23, F24, F25, F27, F28, F29)	**21** (F2, F3, F4, F5, F6, F7, F8, F9, F11, F14, F16, F17, F19, F20, F21, F22, F24, F25, F27, F28, F29)	**19** (F2, F3, F4, F5, F6, F7, F8, F9, F16, F17, F20, F21, F22, F23, F24, F25, F27, F28, F29)
	D=50	**1** (F6)	**6** (F6, F20, F23, F24, F25, F27)	**1** (F6)	**8** (F6, F8, F20, F23, F24, F25, F27, F28)
FA	D=10	**18** (F5, F6, F7, F8, F14, F15, F16, F17, F19, F20, F21, F22, F23, F24, F25, F27, F28, F29)	**17** (F5, F6, F7, F8, F10, F14, F16, F17, F20, F21, F22, F23, F24, F25, F27, F28, F29)	**16** (F5, F6, F7, F8, F14, F17, F19, F20, F21, F22, F23, F24, F25, F27, F28, F29)	**17** (F5, F6, F7, F8, F10, F14, F16, F17, F20, F21, F22, F23, F24, F25, F27, F28, F29)
	D=50	**1** (F6)	**5** (F6, F20, F23, F24, F25)	**1** (F6)	**5** (F6, F20, F23, F24, F25)
BA	D=10	**22** (F4, F5, F6, F7, F8, F9, F11, F14, F15, F16, F17, F19, F20, F21, F22, F23, F24, F25, F26, F27, F28, F29)	**21** (F4, F5, F6, F7, F8, F9, F11, F14, F15, F16, F17, F19, F20, F21, F22, F23, F24, F25, F27, F28, F29)	**22** (F4, F5, F6, F7, F8, F9, F11, F14, F15, F16, F17, F19, F20, F21, F22, F23, F24, F25, F26, F27, F28, F29)	**19** (F4, F5, F6, F7, F8, F11, F14, F16, F17, F19, F20, F21, F22, F23, F24, F25, F27, F28, F29)
	D=50	**1** (F6)	**8** (F6, F20, F23, F24, F25, F26, F27, F28)	**1** (F6)	**5** (F6, F21, F23, F24, F27)
GA	D=10	**23** (F4, F5, F6, F7, F8, F9, F10, F11, F13, F14, F15, F16, F17, F20, F21, F22, F23, F24, F25, F26, F27, F28, F29)	**18** (F4, F5, F6, F7, F8, F9, F16, F17, F20, F21, F22, F23, F24, F25, F26, F27, F28, F29)	**23** (F4, F5, F6, F7, F8, F9, F10, F11, F13, F14, F15, F16, F17, F20, F21, F22, F23, F24, F25, F26, F27, F28, F29)	**17** (F4, F5, F6, F7, F8, F16, F17, F20, F21, F22, F23, F24, F25, F26, F27, F28, F29)
	D=50	**1** (F6)	**9** (F6, F8, F20, F23, F24, F25, F27, F28, F29)	**1** (F6)	**7** (F6, F8, F20, F23, F24, F27, F29)
IA	D=10	**16** (F5, F6, F7, F8, F16, F17, F19, F20, F21, F22, F23, F24, F25, F27, F28, F29)	**15** (F5, F6, F7, F8, F16, F17, F20, F21, F22, F23, F24, F25, F27, F28, F29)	**14** (F5, F6, F7, F8, F17, F20, F21, F22, F23, F24, F25, F27, F28, F29)	**15** (F5, F6, F7, F8, F16, F17, F20, F21, F22, F23, F24, F25, F27, F28, F29)
	D=50	**1** (F6)	**5** (F6, F20, F23, F24, F25)	**1** (F6)	**4** (F6, F20, F23, F24)

Supplementary Material K

Table S27. BBOB functions comparison of mean error(AVERAGE±STD) under D=10 on parameters I for 11 compared NIOAs

Problems	GA	PSO	ABC	BA	IA	FA	CS	DE	GSA	GWO	HS
F1	4.25E+05	4.06E+09	2.35E+06	6.76E+08	9.13E+09	1.58E+10	4.08E+03	5.95E+03	**2.46E+02**	1.46E+05	3.37E+08
	±3.58E+05	±1.31E+09	±2.79E+06	±1.89E+08	±4.60E+09	±5.63E+09	±3.46E+03	±5.28E+03	**±2.02E+02**	±5.07E+05	±2.71E+08
F2	3.40E+02	1.33E+03	2.28E+02	4.05E+02	5.10E+03	7.39E+03	**2.00E+02**	2.00E+02	2.11E+02	2.95E+02	7.39E+02
	±7.24E+01	±4.25E+02	±2.93E+01	±6.06E+01	±2.85E+03	±2.93E+03	**±0.00E+00**	±3.97E-01	±7.71E+00	±1.02E+02	±3.25E+02
F3	2.55E+04	2.83E+04	8.63E+03	2.55E+03	2.76E+04	1.73E+04	**3.00E+02**	8.35E+03	7.57E+03	9.81E+02	2.45E+04
	±1.38E+04	±1.13E+04	±2.72E+03	±7.32E+02	±1.86E+04	±4.13E+03	**±0.00E+00**	±2.70E+03	±1.11E+03	±1.11E+03	±7.95E+03
F4	4.29E+02	6.58E+02	4.06E+02	4.33E+02	1.23E+03	1.98E+03	**4.00E+02**	4.04E+02	4.06E+02	4.14E+02	4.38E+02
	±2.65E+01	±7.64E+01	±2.08E+00	±8.65E+00	±4.72E+02	±7.13E+02	**±1.66E-01**	±1.45E+00	±2.38E-01	±1.79E+01	±1.91E+01
F5	5.50E+02	5.76E+02	5.16E+02	5.37E+02	5.93E+02	6.14E+02	5.14E+02	**5.07E+02**	5.53E+02	5.10E+02	5.43E+02
	±6.67E+00	±5.66E+00	±4.99E+00	±4.54E+00	±2.32E+01	±1.18E+01	±3.64E+00	**±1.70E+00**	±6.52E+00	±5.34E+00	±6.85E+00
F6	6.28E+02	6.47E+02	6.01E+02	6.20E+02	6.54E+02	6.58E+02	6.05E+02	**6.00E+02**	6.18E+02	6.01E+02	6.06E+02
	±4.85E+00	±7.07E+00	±3.11E-01	±5.55E+00	±1.49E+01	±5.08E+00	±1.63E+00	**±0.00E+00**	±1.00E+01	±1.03E+00	±2.25E+00
F7	7.89E+02	9.57E+02	7.34E+02	7.93E+02	8.47E+02	1.07E+03	7.28E+02	7.18E+02	**7.12E+02**	7.27E+02	7.67E+02
	±2.13E+01	±4.57E+01	±6.40E+00	±1.23E+01	±1.77E+01	±4.69E+01	±3.12E+00	±1.91E+00	**±9.79E-01**	±8.06E+00	±8.21E+00
F8	8.59E+02	8.93E+02	8.16E+02	8.39E+02	8.82E+02	8.89E+02	8.17E+02	**8.07E+02**	8.20E+02	8.11E+02	8.42E+02
	±1.15E+01	±1.17E+01	±5.30E+00	±8.07E+00	±1.59E+01	±1.04E+01	±4.08E+00	**±1.98E+00**	±3.84E+00	±5.11E+00	±9.04E+00
F9	9.95E+02	2.52E+03	9.22E+02	1.09E+03	2.10E+03	2.15E+03	9.34E+02	**9.00E+02**	**9.00E+02**	9.10E+02	9.83E+02
	±1.39E+02	±6.12E+02	±2.23E+01	±4.09E+01	±7.32E+02	±1.94E+02	±1.80E+01	**±0.00E+00**	**±0.00E+00**	±1.96E+01	±3.70E+01
F10	1.73E+03	2.24E+03	1.51E+03	2.31E+03	3.13E+03	2.42E+03	1.53E+03	**1.41E+03**	2.76E+03	1.47E+03	2.93E+03
	±2.62E+02	±2.81E+02	±1.59E+02	±2.10E+02	±3.20E+02	±9.35E+01	±8.20E+01	**±9.62E+01**	±3.51E+02	±2.44E+02	±1.69E+02
F11	1.68E+03	1.95E+03	1.13E+03	1.23E+03	9.11E+03	2.26E+03	**1.10E+03**	1.10E+03	1.13E+03	1.12E+03	2.33E+03

	±5.48E+02	±4.84E+02	±2.21E+01	±5.18E+01	±6.69E+03	±6.17E+02	**±7.75E-01**	±1.25E+00	±9.34E+00	±1.01E+01	±8.51E+02
F12	1.27E+06	4.98E+07	7.59E+05	1.18E+07	6.25E+08	1.23E+09	**5.13E+03**	2.07E+05	4.91E+05	4.84E+05	1.74E+07
	±1.56E+06	±4.80E+07	±7.08E+05	±6.32E+06	±3.95E+08	±6.33E+08	**±1.60E+03**	±1.40E+05	±2.74E+05	±7.38E+05	±1.51E+07
F13	1.39E+04	1.87E+05	5.27E+03	5.15E+04	7.58E+07	1.78E+07	**1.32E+03**	2.84E+03	1.15E+04	9.48E+03	1.07E+05
	±1.03E+04	±1.68E+05	±3.52E+03	±3.79E+04	±7.38E+07	±2.74E+07	**±4.97E+00**	±1.52E+03	±1.78E+03	±6.11E+03	±2.08E+05
F14	7.30E+03	4.86E+03	1.63E+03	1.58E+03	1.94E+06	1.51E+03	**1.42E+03**	1.42E+03	5.75E+03	2.17E+03	6.63E+03
	±7.71E+03	±8.25E+03	±1.58E+02	±5.69E+01	±3.45E+06	±4.26E+01	**±4.06E+00**	±1.48E+01	±9.87E+02	±1.45E+03	±4.51E+03
F15	1.09E+04	2.48E+04	2.08E+03	2.16E+03	5.60E+06	2.73E+03	**1.51E+03**	1.51E+03	1.80E+04	2.69E+03	1.01E+04
	±8.66E+03	±2.92E+04	±6.01E+02	±3.64E+02	±1.32E+07	±5.99E+02	**±1.49E+00**	±1.46E+01	±3.49E+03	±1.48E+03	±1.20E+04
F16	1.71E+03	1.81E+03	1.65E+03	1.76E+03	2.43E+03	2.08E+03	**1.60E+03**	1.62E+03	2.16E+03	1.70E+03	1.91E+03
	±7.47E+01	±1.56E+02	±5.83E+01	±1.04E+02	±1.41E+02	±6.58E+01	**±1.15E+00**	±1.32E+01	±8.99E+01	±1.16E+02	±1.18E+02
F17	1.85E+03	1.91E+03	1.72E+03	1.81E+03	2.16E+03	1.87E+03	1.73E+03	**1.72E+03**	1.82E+03	1.74E+03	1.79E+03
	±8.79E+01	±5.75E+01	±1.01E+01	±4.97E+01	±1.67E+02	±3.31E+01	±2.46E+00	**±8.80E+00**	±9.58E+01	±1.40E+01	±5.08E+01
F18	2.46E+04	2.11E+05	7.19E+03	6.45E+04	1.65E+08	1.85E+08	**1.93E+03**	2.35E+03	7.81E+03	3.03E+04	2.05E+05
	±1.24E+04	±1.66E+05	±3.41E+03	±3.23E+04	±1.28E+08	±1.89E+08	**±5.02E+01**	±5.44E+02	±3.09E+03	±1.47E+04	±3.58E+05
F19	2.24E+04	4.70E+04	2.63E+03	2.45E+03	9.67E+06	4.09E+03	1.91E+03	**1.90E+03**	3.18E+04	4.79E+03	6.96E+03
	±1.39E+04	±3.51E+04	±1.15E+03	±4.33E+02	±1.47E+07	±3.33E+03	±9.63E-01	**±3.89E+00**	±9.06E+03	±5.07E+03	±5.24E+03
F20	2.06E+03	2.17E+03	2.02E+03	2.13E+03	2.36E+03	2.14E+03	2.02E+03	**2.01E+03**	2.26E+03	2.04E+03	2.04E+03
	±1.86E+01	±4.67E+01	±9.44E+00	±6.86E+01	±7.07E+01	±2.22E+01	±3.44E+00	**±7.21E+00**	±4.70E+01	±2.75E+01	±1.23E+01
F21	2.32E+03	2.31E+03	2.22E+03	2.28E+03	2.37E+03	2.37E+03	**2.20E+03**	2.23E+03	2.35E+03	2.29E+03	2.35E+03
	±4.55E+01	±7.76E+01	±7.47E+00	±6.79E+01	±4.73E+01	±3.20E+01	**±7.03E-01**	±1.68E+01	±1.54E+01	±5.04E+01	±7.17E+00
F22	2.43E+03	2.77E+03	2.28E+03	2.38E+03	3.08E+03	3.54E+03	**2.26E+03**	2.27E+03	2.30E+03	2.30E+03	2.34E+03
	±1.70E+02	±1.79E+02	±3.26E+01	±2.27E+01	±3.29E+02	±3.23E+02	**±2.90E+01**	±2.04E+01	±0.00E+00	±2.13E+01	±1.55E+01
F23	2.65E+03	2.66E+03	**2.61E+03**	2.64E+03	2.76E+03	2.79E+03	2.61E+03	2.62E+03	2.72E+03	2.61E+03	2.64E+03
	±9.79E+00	±1.41E+01	**±7.15E+01**	±5.63E+00	±3.22E+01	±3.67E+01	±2.43E+00	±2.37E+00	±3.56E+01	±7.87E+00	±7.59E+00
F24	2.74E+03	2.79E+03	2.56E+03	2.75E+03	2.89E+03	2.95E+03	**2.51E+03**	2.65E+03	2.53E+03	2.74E+03	2.77E+03

	±3.84E+01	±2.67E+01	±7.38E+01	±5.79E+01	±4.57E+01	±5.18E+01	**±2.33E+01**	±6.41E+01	±9.39E+01	±1.34E+01	±2.09E+01
F25	2.97E+03	3.13E+03	2.91E+03	2.96E+03	3.41E+03	3.98E+03	**2.70E+03**	2.90E+03	2.94E+03	2.93E+03	2.97E+03
	±2.52E+01	±5.52E+01	±5.95E+01	±1.10E+01	±1.38E+02	±3.86E+02	**±1.06E+02**	±1.83E+00	±1.60E-02	±1.93E+01	±1.17E+01
F26	3.12E+03	3.41E+03	2.92E+03	3.24E+03	4.08E+03	4.44E+03	**2.69E+03**	2.84E+03	3.09E+03	2.91E+03	3.68E+03
	±1.17E+02	±3.70E+02	±9.46E+01	±4.09E+02	±3.11E+02	±2.95E+02	**±9.87E+01**	±1.13E+02	±5.55E+02	±5.16E+01	±4.27E+02
F27	3.12E+03	3.12E+03	3.10E+03	3.11E+03	3.27E+03	3.33E+03	3.09E+03	**3.07E+03**	3.23E+03	3.09E+03	3.11E+03
	±9.06E+00	±2.18E+01	±5.30E+00	±2.12E+01	±6.35E+01	±4.40E+01	±1.71E+00	**±3.52E+00**	±2.36E+01	±3.34E+00	±8.09E+00
F28	3.50E+03	3.40E+03	3.23E+03	3.32E+03	3.83E+03	3.91E+03	**3.12E+03**	3.27E+03	3.38E+03	3.34E+03	3.52E+03
	±5.07E+01	±1.02E+02	±1.34E+02	±1.04E+02	±1.55E+02	±1.61E+02	**±2.50E+01**	±1.62E+00	±3.08E+01	±1.01E+02	±5.45E+01
F29	3.21E+03	3.28E+03	3.19E+03	3.24E+03	3.63E+03	3.43E+03	3.17E+03	3.17E+03	3.36E+03	**3.17E+03**	3.34E+03
	±1.58E+01	±8.15E+01	±2.52E+01	±7.68E+01	±1.70E+02	±5.82E+01	±9.61E+00	±9.81E+00	±7.98E+01	**±1.64E+01**	±5.63E+01
F30	1.08E+06	1.68E+06	1.79E+05	6.55E+05	4.17E+07	3.10E+07	1.12E+04	**3.80E+03**	7.17E+05	4.19E+05	5.75E+06
	±8.73E+05	±2.49E+06	±1.88E+05	±4.72E+05	±4.35E+07	±1.41E+07	±6.75E+03	**±5.66E+02**	±1.10E+05	±6.44E+05	±2.37E+06

Table S28. BBOB functions comparison of mean error(AVERAGE ± STD) under D=50 on parameters I for 11 compared NIOAs

Problems	GA	PSO	ABC	BA	IA	FA	CS	DE	GSA	GWO	HS
F1	3.52E+09	1.76E+11	5.15E+07	3.17E+10	8.84E+10	1.94E+11	**1.14E+03**	5.59E+03	1.27E+03	4.82E+09	1.87E+07
	±3.58E+09	±1.95E+10	±2.11E+07	±4.45E+09	±1.44E+10	±9.01E+09	**±1.55E+03**	±4.26E+03	±1.26E+03	±2.38E+09	±2.76E+06
F2	1.16E+03	4.39E+04	3.98E+02	6.76E+03	2.91E+04	6.68E+04	2.00E+02	1.58E+03	**2.00E+02**	1.42E+03	9.63E+02
	±6.38E+02	±1.32E+04	±4.60E+01	±7.06E+02	±1.24E+04	±1.20E+04	±1.95E-04	±5.75E+02	**±5.34E-05**	±3.22E+02	±1.94E+02
F3	3.57E+05	5.08E+05	2.22E+05	7.55E+04	2.06E+05	2.24E+05	**3.06E+04**	1.78E+05	1.42E+05	6.32E+04	3.10E+05
	±8.70E+04	±1.07E+05	±2.17E+04	±1.27E+04	±1.96E+04	±1.57E+04	**±3.23E+03**	±6.76E+04	±1.06E+04	±1.60E+04	±4.41E+04
F4	7.76E+02	3.87E+04	6.03E+02	2.81E+03	2.25E+04	6.65E+04	**4.44E+02**	4.46E+02	5.18E+02	8.27E+02	6.15E+02
	±6.52E+01	±6.76E+03	±3.53E+01	±5.39E+02	±6.26E+03	±4.49E+03	**±2.19E+01**	±8.84E-01	±4.14E+01	±1.50E+02	±3.44E+01
F5	1.03E+03	1.47E+03	7.58E+02	1.02E+03	1.11E+03	1.42E+03	7.83E+02	6.98E+02	8.23E+02	**6.68E+02**	6.76E+02

	±2.71E+01	±6.36E+01	±3.06E+01	±3.37E+01	±5.07E+01	±2.66E+01	±2.63E+01	±1.89E+01	±1.73E+01	**±1.89E+01**	±8.74E+01
F6	6.87E+02	7.19E+02	6.03E+02	6.61E+02	6.82E+02	7.09E+02	6.48E+02	**6.00E+02**	6.50E+02	6.08E+02	6.03E+02
	±5.76E+00	±5.51E+00	±9.36E-01	±5.51E+00	±9.33E+00	±2.29E+00	±6.21E+00	**±0.00E+00**	±4.18E+00	±3.41E+00	±1.31E+00
F7	1.70E+03	5.17E+03	1.12E+03	2.08E+03	1.85E+03	4.87E+03	1.02E+03	9.58E+02	**7.89E+02**	9.75E+02	1.15E+03
	±1.68E+02	±2.95E+02	±2.73E+01	±1.21E+02	±1.08E+02	±1.33E+02	±3.31E+01	±2.62E+01	**±1.05E+01**	±3.74E+01	±4.79E+01
F8	1.36E+03	1.75E+03	1.06E+03	1.32E+03	1.41E+03	1.74E+03	1.09E+03	1.00E+03	1.14E+03	9.82E+02	**9.25E+02**
	±2.13E+01	±3.71E+01	±2.48E+01	±3.10E+01	±4.56E+01	±3.06E+01	±3.42E+01	±2.15E+01	±1.48E+01	±3.67E+01	**±9.21E+01**
F9	2.26E+03	6.44E+04	1.24E+04	1.54E+04	2.15E+04	4.36E+04	1.41E+04	**1.36E+03**	4.59E+03	4.47E+03	1.85E+03
	±7.15E+02	±1.21E+04	±2.07E+03	±3.47E+03	±2.80E+03	±2.66E+03	±2.85E+03	**±7.48E+02**	±6.52E+02	±2.26E+03	±4.01E+02
F10	1.28E+04	1.39E+04	6.52E+03	1.42E+04	1.24E+04	1.41E+04	7.07E+03	9.19E+03	7.39E+03	**6.24E+03**	1.49E+04
	±2.99E+02	±6.68E+02	±4.16E+02	±4.69E+02	±6.41E+02	±2.61E+02	±2.31E+02	±5.28E+02	±6.37E+02	**±5.87E+02**	±4.46E+02
F11	1.65E+04	3.92E+04	4.45E+03	4.87E+03	2.64E+04	2.58E+04	1.23E+03	**1.21E+03**	1.22E+03	3.00E+03	6.22E+03
	±8.96E+03	±1.13E+04	±1.44E+03	±9.35E+02	±8.97E+03	±3.18E+03	±1.46E+01	**±4.00E+01**	±1.08E+01	±1.58E+03	±2.89E+03
F12	3.15E+07	4.38E+10	2.99E+07	7.21E+09	4.86E+10	8.78E+10	**6.72E+05**	2.64E+07	8.56E+05	2.94E+08	3.47E+07
	±1.98E+07	±8.67E+09	±9.92E+06	±1.08E+09	±1.52E+10	±1.26E+10	**±2.50E+05**	±1.68E+07	±2.26E+05	±3.44E+08	±1.78E+07
F13	1.47E+08	1.70E+10	1.36E+06	2.55E+09	3.82E+10	4.44E+10	**9.68E+03**	2.12E+04	1.79E+04	1.35E+08	8.43E+05
	±3.87E+08	±6.10E+09	±7.65E+05	±4.67E+08	±8.70E+09	±4.61E+09	**±7.47E+03**	±1.59E+04	±2.38E+03	±1.79E+08	±1.42E+05
F14	6.61E+06	1.56E+07	1.34E+06	9.07E+05	9.63E+07	2.17E+07	**1.88E+03**	5.31E+05	1.98E+04	1.92E+05	2.01E+06
	±4.16E+06	±1.88E+07	±6.56E+05	±3.74E+05	±4.64E+07	±1.12E+07	**±1.86E+02**	±3.06E+05	±7.27E+03	±1.38E+05	±2.18E+06
F15	3.61E+05	3.12E+09	4.63E+05	6.61E+08	1.14E+10	1.06E+10	**2.71E+03**	1.60E+04	1.22E+04	4.65E+06	1.48E+05
	±6.76E+05	±1.55E+09	±3.06E+05	±2.05E+08	±4.42E+09	±2.11E+09	**±8.10E+02**	±1.08E+04	±3.48E+03	±8.68E+06	±2.85E+04
F16	3.61E+03	6.88E+03	3.16E+03	5.00E+03	7.12E+03	8.97E+03	3.19E+03	**2.84E+03**	3.52E+03	2.84E+03	4.84E+03
	±4.60E+02	±4.84E+02	±3.00E+02	±2.68E+02	±1.16E+03	±4.57E+02	±1.27E+02	**±2.51E+02**	±3.25E+02	±2.57E+02	±3.23E+02
F17	3.22E+03	2.29E+04	3.06E+03	4.73E+03	2.28E+04	2.20E+04	2.81E+03	2.67E+03	3.56E+03	**2.62E+03**	3.73E+03
	±3.94E+02	±1.77E+04	±1.26E+02	±3.80E+02	±2.01E+04	±9.93E+03	±1.57E+02	±2.27E+02	±3.37E+02	**±1.68E+02**	±3.73E+02
F18	1.17E+07	6.57E+07	2.69E+06	1.03E+07	1.73E+08	7.47E+07	**1.41E+05**	1.34E+06	1.84E+05	2.19E+06	1.82E+07

	±8.47E+06	±5.63E+07	±1.23E+06	±4.51E+06	±1.08E+08	±2.07E+07	**±3.36E+04**	±4.95E+05	±4.90E+04	±3.67E+06	±9.76E+06
F19	9.53E+05	2.30E+09	2.03E+05	3.21E+08	5.62E+09	4.72E+09	**2.35E+03**	7.57E+03	1.69E+04	1.60E+06	7.79E+04
	±2.05E+06	±1.02E+09	±8.59E+04	±8.09E+07	±2.64E+09	±1.14E+09	**±3.39E+02**	±1.73E+03	±2.60E+03	±2.94E+06	±2.10E+04
F20	3.50E+03	4.21E+03	2.94E+03	3.75E+03	4.46E+03	3.85E+03	2.90E+03	**2.49E+03**	3.44E+03	2.68E+03	4.00E+03
	±1.87E+02	±2.28E+02	±2.10E+02	±1.96E+02	±2.52E+02	±8.99E+01	±1.19E+02	**±1.25E+02**	±2.69E+02	±2.19E+02	±1.94E+02
F21	2.93E+03	3.20E+03	2.58E+03	2.80E+03	2.93E+03	3.27E+03	2.56E+03	2.52E+03	2.69E+03	**2.47E+03**	2.57E+03
	±2.52E+01	±4.86E+01	±2.27E+01	±4.07E+01	±7.28E+01	±3.78E+01	±3.30E+01	±1.87E+01	±3.10E+01	**±2.46E+01**	±9.11E+01
F22	1.45E+04	1.53E+04	8.22E+03	1.58E+04	1.46E+04	1.58E+04	8.96E+03	1.08E+04	1.11E+04	**8.20E+03**	1.63E+04
	±5.08E+02	±7.23E+02	±5.14E+02	±4.26E+02	±8.57E+02	±2.88E+02	±2.31E+02	±1.55E+03	±5.02E+02	**±6.51E+02**	±5.03E+02
F23	3.44E+03	3.88E+03	3.05E+03	3.25E+03	4.00E+03	4.59E+03	2.99E+03	2.90E+03	4.02E+03	2.91E+03	**2.83E+03**
	±4.88E+01	±1.16E+02	±3.73E+01	±2.64E+01	±1.48E+02	±1.03E+02	±2.40E+01	±2.01E+01	±1.30E+02	±3.28E+01	**±1.82E+01**
F24	3.58E+03	3.88E+03	3.58E+03	3.37E+03	4.38E+03	5.11E+03	3.15E+03	3.28E+03	3.54E+03	**3.08E+03**	3.28E+03
	±3.69E+01	±1.38E+02	±8.35E+01	±3.27E+01	±1.36E+02	±1.16E+02	±2.34E+01	±3.74E+01	±5.55E+01	**±8.31E+01**	±1.82E+01
F25	3.61E+03	3.60E+04	3.11E+03	4.98E+03	1.27E+04	3.70E+04	2.98E+03	**2.93E+03**	3.05E+03	3.33E+03	3.11E+03
	±9.51E+01	±5.90E+03	±2.66E+01	±4.14E+02	±2.20E+03	±2.30E+03	±2.19E+01	**±3.67E-02**	±3.06E+01	±1.58E+02	±5.21E+01
F26	8.99E+03	1.59E+04	6.85E+03	9.17E+03	1.51E+04	2.40E+04	4.85E+03	5.33E+03	**2.90E+03**	5.83E+03	4.73E+03
	±3.53E+02	±1.46E+03	±1.06E+03	±3.03E+02	±1.23E+03	±9.12E+02	±1.59E+03	±1.90E+02	**±0.00E+00**	±3.53E+02	±2.16E+02
F27	3.87E+03	4.34E+03	3.45E+03	3.56E+03	5.99E+03	6.77E+03	3.29E+03	**3.20E+03**	5.37E+03	3.47E+03	3.46E+03
	±6.97E+01	±2.94E+02	±3.54E+01	±9.19E+01	±4.64E+02	±2.72E+02	±3.75E+01	**±2.19E-04**	±3.42E+02	±7.03E+01	±5.56E+01
F28	3.94E+03	1.24E+04	3.37E+03	4.36E+03	1.05E+04	1.82E+04	**3.29E+03**	3.30E+03	3.30E+03	3.95E+03	3.63E+03
	±1.17E+02	±1.38E+03	±3.32E+01	±2.06E+02	±1.19E+03	±9.98E+02	**±1.97E+01**	±2.69E-04	±1.44E+01	±2.78E+02	±1.28E+02
F29	4.23E+03	9.70E+03	4.14E+03	6.16E+03	3.37E+04	4.13E+04	4.13E+03	**3.98E+03**	5.00E+03	4.11E+03	4.06E+03
	±2.49E+02	±1.73E+03	±1.76E+02	±4.68E+02	±3.26E+04	±1.61E+04	±8.21E+01	**±2.52E+02**	±2.32E+02	±3.11E+02	±4.95E+02
F30	2.88E+06	2.85E+09	1.69E+06	6.41E+08	6.59E+09	7.51E+09	1.08E+06	**1.32E+05**	7.06E+06	7.14E+07	4.01E+06
	±7.01E+05	±1.31E+09	±4.33E+05	±1.36E+08	±2.96E+09	±1.10E+09	±1.94E+05	**±9.94E+04**	±4.02E+05	±3.56E+07	±6.01E+05

Table S29. BBOB functions comparison of mean error(AVERAGE ± STD) under D=10 on parameters II for 11 compared NIOAs

Problems	GA	PSO	ABC	BA	IA	FA	CS	DE	GSA	GWO	HS
F1	2.20E+06	1.86E+07	4.79E+06	1.54E+09	1.07E+10	2.13E+10	5.18E+02	9.75E+02	**3.32E+02**	2.51E+07	3.71E+08
	±3.66E+06	±4.18E+06	±3.61E+06	±4.98E+08	±4.59E+09	±6.80E+09	±2.88E+02	±1.31E+03	**±3.56E+02**	±1.04E+08	±2.03E+08
F2	3.79E+02	2.11E+02	2.70E+02	6.03E+02	6.86E+03	1.03E+04	**2.00E+02**	2.00E+02	2.00E+02	2.83E+02	7.39E+02
	±9.02E+01	±4.14E+00	±5.21E+01	±9.36E+01	±5.85E+03	±4.78E+03	**±0.00E+00**	±6.46E-03	±6.82E-05	±3.54E+01	±2.98E+02
F3	2.26E+04	3.52E+02	1.30E+04	6.34E+03	3.11E+04	2.27E+04	**3.00E+02**	5.02E+03	3.76E+03	2.08E+03	2.16E+04
	±1.27E+04	±1.35E+01	±4.00E+03	±2.10E+03	±1.93E+04	±4.19E+03	**±0.00E+00**	±2.24E+03	±1.14E+03	±1.98E+03	±5.04E+03
F4	4.44E+02	4.11E+02	4.12E+02	4.71E+02	1.47E+03	2.51E+03	**4.00E+02**	4.05E+02	4.05E+02	4.23E+02	4.43E+02
	±2.58E+01	±1.56E+01	±1.45E+01	±2.32E+01	±6.68E+02	±1.13E+03	**±7.82E-02**	±8.42E-01	±2.94E-01	±2.32E+01	±2.14E+01
F5	5.58E+02	5.37E+02	5.20E+02	5.48E+02	5.94E+02	6.23E+02	5.13E+02	**5.08E+02**	5.58E+02	5.17E+02	5.45E+02
	±1.27E+01	±7.23E+00	±5.21E+00	±3.27E+00	±1.90E+01	±1.18E+01	±3.79E+00	**±2.30E+00**	±9.32E+00	±8.87E+00	±5.93E+00
F6	6.36E+02	6.06E+02	6.02E+02	6.29E+02	6.60E+02	6.64E+02	6.02E+02	**6.00E+02**	6.17E+02	6.00E+02	6.08E+02
	±7.07E+00	±2.33E+00	±1.02E+00	±7.86E+00	±1.39E+01	±4.90E+00	±1.11E+00	**±0.00E+00**	±9.73E+00	±3.68E-01	±2.93E+00
F7	8.24E+02	7.39E+02	7.42E+02	8.33E+02	8.43E+02	1.09E+03	7.25E+02	7.20E+02	**7.13E+02**	7.24E+02	7.80E+02
	±3.65E+01	±3.86E+00	±8.49E+00	±2.30E+01	±2.06E+01	±5.87E+01	±4.53E+00	±2.70E+00	**±1.30E+00**	±4.83E+00	±1.23E+01
F8	8.65E+02	8.28E+02	8.21E+02	8.56E+02	8.93E+02	8.97E+02	8.13E+02	**8.08E+02**	8.22E+02	8.15E+02	8.47E+02
	±1.44E+01	±6.97E+00	±5.85E+00	±5.54E+00	±1.45E+01	±8.93E+00	±3.31E+00	**±1.55E+00**	±4.76E+00	±5.00E+00	±6.53E+00
F9	1.12E+03	9.06E+02	9.78E+02	1.43E+03	2.32E+03	2.37E+03	9.04E+02	**9.00E+02**	**9.00E+02**	9.05E+02	1.07E+03
	±2.53E+02	±1.48E+00	±8.45E+01	±1.38E+02	±6.84E+02	±2.44E+02	±4.04E+00	**±0.00E+00**	**±0.00E+00**	±9.10E+00	±7.87E+01
F10	2.06E+03	1.95E+03	1.60E+03	2.33E+03	3.11E+03	2.57E+03	1.63E+03	1.51E+03	2.72E+03	**1.48E+03**	2.85E+03
	±2.02E+02	±2.00E+02	±2.16E+02	±2.69E+02	±2.66E+02	±1.16E+02	±1.35E+02	±1.25E+02	±2.96E+02	**±2.73E+02**	±1.66E+02
F11	2.17E+03	1.13E+03	1.17E+03	1.35E+03	8.35E+03	4.26E+03	**1.10E+03**	1.10E+03	1.13E+03	1.13E+03	1.97E+03
	±8.71E+02	±9.80E+00	±6.10E+01	±8.82E+01	±6.25E+03	±2.48E+03	**±5.53E-01**	±1.04E+00	±6.13E+00	±3.97E+01	±1.11E+03

F12	1.76E+06 ±2.17E+06	1.70E+06 ±1.50E+06	1.39E+06 ±1.49E+06	3.02E+07 ±1.73E+07	8.07E+08 ±5.91E+08	1.60E+09 ±6.94E+08	**4.26E+03 ±1.96E+03**	1.74E+05 ±1.23E+05	2.29E+05 ±2.51E+05	5.72E+05 ±6.24E+05	2.17E+07 ±1.61E+07
F13	1.68E+04 ±1.86E+04	1.13E+04 ±5.38E+03	1.07E+04 ±8.00E+03	1.82E+05 ±1.68E+05	1.16E+08 ±1.34E+08	6.50E+07 ±7.11E+07	**1.31E+03 ±5.79E+00**	1.80E+03 ±5.15E+02	1.01E+04 ±2.31E+03	1.01E+04 ±8.25E+03	2.63E+04 ±5.04E+04
F14	1.17E+04 ±8.70E+03	1.50E+03 ±5.53E+01	1.94E+03 ±5.39E+02	1.73E+03 ±1.60E+02	5.08E+06 ±1.17E+07	1.62E+03 ±2.02E+02	**1.42E+03 ±4.79E+00**	1.42E+03 ±6.47E+00	6.47E+03 ±1.95E+03	1.83E+03 ±1.08E+03	8.67E+03 ±8.00E+03
F15	9.22E+03 ±7.32E+03	2.08E+03 ±4.45E+02	3.00E+03 ±1.80E+03	3.61E+03 ±1.88E+03	9.80E+06 ±1.56E+07	3.63E+03 ±1.42E+03	1.50E+03 ±1.68E+00	**1.50E+03 ±7.57E-01**	1.49E+04 ±4.56E+03	3.31E+03 ±2.47E+03	7.18E+03 ±5.54E+03
F16	1.76E+03 ±8.28E+01	1.84E+03 ±9.32E+01	1.74E+03 ±8.32E+01	1.78E+03 ±5.14E+01	2.52E+03 ±1.66E+02	2.19E+03 ±1.15E+02	**1.60E+03 ±4.03E+00**	1.62E+03 ±1.77E+01	2.12E+03 ±1.08E+02	1.66E+03 ±4.72E+01	1.85E+03 ±1.68E+02
F17	1.88E+03 ±9.39E+01	1.77E+03 ±2.62E+01	1.74E+03 ±2.05E+01	1.83E+03 ±5.62E+01	2.25E+03 ±1.74E+02	1.96E+03 ±7.76E+01	1.73E+03 ±4.30E+00	**1.72E+03 ±7.53E+00**	1.89E+03 ±1.12E+02	1.74E+03 ±1.43E+01	1.78E+03 ±4.95E+01
F18	3.99E+04 ±3.01E+04	1.74E+04 ±1.35E+04	1.38E+04 ±1.26E+04	1.47E+05 ±8.19E+04	1.80E+08 ±1.73E+08	3.57E+08 ±2.88E+08	1.91E+03 ±3.64E+01	**1.82E+03 ±3.23E+00**	7.92E+03 ±4.77E+03	3.19E+04 ±1.45E+04	2.95E+05 ±8.14E+05
F19	2.50E+04 ±2.31E+04	3.77E+03 ±6.89E+03	3.11E+03 ±1.31E+03	6.20E+03 ±4.61E+03	3.46E+07 ±3.59E+07	1.30E+06 ±5.61E+06	1.90E+03 ±1.22E+00	**1.90E+03 ±7.58E-01**	9.64E+03 ±2.56E+03	7.67E+03 ±5.64E+03	8.90E+03 ±6.50E+03
F20	2.09E+03 ±1.84E+01	2.11E+03 ±5.87E+01	2.03E+03 ±1.33E+01	2.14E+03 ±5.09E+01	2.40E+03 ±1.10E+02	2.17E+03 ±3.80E+01	2.03E+03 ±4.12E+00	**2.01E+03 ±8.38E+00**	2.30E+03 ±1.05E+02	2.06E+03 ±4.68E+01	2.04E+03 ±1.03E+01
F21	2.33E+03 ±4.53E+01	2.31E+03 ±5.52E+01	2.24E+03 ±3.03E+01	2.29E+03 ±7.01E+01	2.38E+03 ±5.03E+01	2.39E+03 ±4.25E+01	**2.20E+03 ±1.74E+00**	2.26E+03 ±4.04E+01	2.35E+03 ±1.34E+01	2.31E+03 ±3.89E+00	2.35E+03 ±9.54E+00
F22	2.49E+03 ±2.86E+02	2.30E+03 ±2.52E+01	2.29E+03 ±2.66E+01	2.53E+03 ±2.86E+02	3.11E+03 ±3.51E+02	3.75E+03 ±3.01E+02	**2.27E+03 ±4.12E+01**	2.29E+03 ±2.20E+01	2.30E+03 ±7.70E-02	2.30E+03 ±2.11E+01	2.35E+03 ±1.58E+01
F23	2.67E+03 ±1.19E+01	2.66E+03 ±2.26E+01	2.63E+03 ±8.38E+00	2.64E+03 ±7.64E+00	2.79E+03 ±4.44E+01	2.82E+03 ±4.04E+01	2.61E+03 ±4.21E+00	**2.60E+03 ±6.14E+01**	2.75E+03 ±6.52E+01	2.62E+03 ±8.39E+00	2.65E+03 ±7.61E+00
F24	2.79E+03 ±2.03E+01	2.71E+03 ±1.09E+02	2.65E+03 ±1.00E+02	2.77E+03 ±3.52E+01	2.90E+03 ±4.84E+01	2.98E+03 ±7.00E+01	**2.54E+03 ±6.57E+01**	2.71E+03 ±5.87E+01	2.58E+03 ±1.37E+02	2.75E+03 ±1.18E+01	2.78E+03 ±8.25E+00

F25	3.02E+03	2.94E+03	2.91E+03	3.01E+03	3.44E+03	4.30E+03	**2.83E+03**	2.90E+03	2.93E+03	2.93E+03	2.97E+03
	±6.42E+01	±2.01E+01	±7.12E+01	±2.36E+01	±2.54E+02	±5.22E+02	**±1.09E+02**	±6.35E+00	±1.98E+01	±1.64E+01	±1.01E+01
F26	3.29E+03	3.01E+03	2.99E+03	3.36E+03	4.32E+03	4.67E+03	**2.78E+03**	2.93E+03	3.47E+03	2.98E+03	3.44E+03
	±2.86E+02	±2.68E+02	±8.11E+01	±4.73E+02	±3.85E+02	±3.49E+02	**±8.39E+01**	±1.03E+02	±6.01E+02	±2.38E+02	±4.58E+02
F27	3.13E+03	3.13E+03	3.10E+03	3.10E+03	3.30E+03	3.34E+03	3.09E+03	**3.08E+03**	3.22E+03	3.09E+03	3.11E+03
	±1.66E+01	±3.48E+01	±9.00E+00	±1.39E+00	±1.09E+02	±8.94E+01	±1.31E+00	**±7.34E+00**	±3.02E+01	±2.36E+00	±7.44E+00
F28	3.48E+03	3.30E+03	3.24E+03	3.40E+03	3.91E+03	4.07E+03	**3.09E+03**	3.27E+03	3.34E+03	3.39E+03	3.49E+03
	±1.07E+02	±1.37E+02	±6.90E+01	±1.02E+02	±2.20E+02	±1.88E+02	**±8.56E+01**	±1.68E+00	±9.42E+01	±6.91E+01	±2.49E+01
F29	3.23E+03	3.25E+03	3.21E+03	3.25E+03	3.55E+03	3.60E+03	3.18E+03	**3.17E+03**	3.36E+03	3.20E+03	3.35E+03
	±2.80E+01	±5.17E+01	±2.73E+01	±4.86E+01	±1.67E+02	±1.45E+02	±9.87E+00	**±1.20E+01**	±1.21E+02	±4.80E+01	±6.79E+01
F30	1.78E+06	5.13E+05	4.59E+05	5.25E+05	6.03E+07	4.69E+07	2.11E+04	**3.79E+03**	2.86E+05	3.67E+05	5.32E+06
	±1.46E+06	±6.79E+05	±4.01E+05	±4.80E+05	±4.59E+07	±2.08E+07	±1.97E+04	**±6.05E+02**	±6.85E+04	±8.11E+05	±2.88E+06

Table S30. BBOB functions comparison of mean error(AVERAGE±STD) under D=50 on parameters II for 11 compared NIOAs

Problems	GA	PSO	ABC	BA	IA	FA	CS	DE	GSA	GWO	HS
F1	1.69E+10	1.63E+09	1.82E+08	5.72E+10	8.72E+10	2.12E+11	**2.56E+03**	2.05E+05	5.60E+03	6.91E+09	1.59E+10
	±1.54E+10	±1.03E+09	±7.86E+07	±8.91E+09	±8.40E+09	±1.12E+10	**±2.95E+03**	±5.27E+05	±8.11E+03	±3.22E+09	±2.99E+09
F2	1.54E+03	5.98E+02	5.70E+02	1.31E+04	3.24E+04	8.62E+04	**2.00E+02**	1.27E+04	4.06E+02	1.73E+03	1.28E+04
	±5.95E+02	±1.28E+02	±8.94E+01	±1.75E+03	±1.34E+04	±1.34E+04	**±7.90E-04**	±7.90E+03	±2.46E+02	±5.70E+02	±2.60E+03
F3	3.60E+05	**2.75E+03**	2.34E+05	1.37E+05	1.87E+05	2.46E+05	2.02E+04	3.43E+05	1.86E+05	7.71E+04	3.01E+05
	±7.89E+04	**±5.64E+02**	±2.50E+04	±2.84E+04	±3.03E+04	±2.12E+04	±2.54E+03	±1.40E+05	±2.90E+04	±1.76E+04	±4.60E+04
F4	1.14E+03	6.77E+02	6.66E+02	5.72E+03	2.37E+04	7.31E+04	**4.38E+02**	4.44E+02	5.13E+02	1.02E+03	2.36E+03
	±2.44E+02	±5.04E+01	±3.99E+01	±1.41E+03	±6.21E+03	±7.54E+03	**±4.42E+01**	±2.06E+00	±6.26E+01	±2.42E+02	±2.65E+02
F5	1.15E+03	9.56E+02	8.24E+02	1.11E+03	1.09E+03	1.47E+03	7.72E+02	8.26E+02	7.98E+02	**6.94E+02**	9.86E+02
	±2.61E+01	±3.35E+01	±2.27E+01	±3.43E+01	±5.45E+01	±3.26E+01	±4.03E+01	±3.40E+01	±4.72E+01	**±3.08E+01**	±2.11E+01

F6	7.00E+02	6.33E+02	6.07E+02	6.78E+02	6.82E+02	7.36E+02	6.33E+02	**6.00E+02**	6.32E+02	6.12E+02	6.22E+02
	±6.03E+00	±1.91E+01	±1.77E+00	±7.43E+00	±6.94E+00	±7.17E+00	±9.06E+00	**±0.00E+00**	±7.62E+00	±4.20E+00	±2.26E+00
F7	2.43E+03	1.14E+03	1.19E+03	3.00E+03	1.80E+03	6.05E+03	1.09E+03	1.06E+03	**9.41E+02**	1.04E+03	1.58E+03
	±2.14E+02	±1.99E+01	±4.66E+01	±3.40E+02	±1.16E+02	±4.25E+02	±6.44E+01	±3.21E+01	**±4.21E+01**	±8.15E+01	±5.16E+01
F8	1.48E+03	1.25E+03	1.12E+03	1.41E+03	1.40E+03	1.97E+03	1.07E+03	1.13E+03	1.09E+03	**9.84E+02**	1.29E+03
	±4.09E+01	±3.27E+01	±4.33E+01	±5.15E+01	±5.53E+01	±6.15E+01	±2.87E+01	±3.40E+01	±4.58E+01	**±2.31E+01**	±2.13E+01
F9	4.20E+03	1.64E+04	1.52E+04	2.54E+04	2.18E+04	6.32E+04	1.18E+04	**2.18E+03**	7.75E+03	4.25E+03	8.66E+03
	±7.17E+02	±7.58E+03	±2.98E+03	±4.75E+03	±3.33E+03	±6.65E+03	±4.16E+03	**±3.02E+03**	±2.61E+03	±1.13E+03	±1.15E+03
F10	1.34E+04	1.18E+04	7.68E+03	1.45E+04	1.26E+04	1.56E+04	7.12E+03	1.25E+04	**5.91E+03**	6.63E+03	1.53E+04
	±2.43E+02	±7.34E+02	±5.32E+02	±4.68E+02	±9.97E+02	±3.46E+02	±3.39E+02	±3.54E+02	**±7.43E+02**	±7.19E+02	±4.86E+02
F11	1.97E+04	1.59E+03	6.42E+03	8.78E+03	3.19E+04	6.12E+04	**1.22E+03**	1.27E+03	1.27E+03	3.89E+03	8.99E+03
	±1.11E+04	±6.96E+01	±2.64E+03	±2.17E+03	±1.12E+04	±1.34E+04	**±2.55E+01**	±6.58E+01	±3.82E+01	±2.03E+03	±3.10E+03
F12	4.25E+07	5.52E+08	8.85E+07	1.43E+10	6.02E+10	1.58E+11	**2.25E+05**	3.42E+08	1.99E+08	6.46E+08	1.95E+09
	±2.09E+07	±6.02E+08	±3.05E+07	±2.46E+09	±1.89E+10	±2.95E+10	**±2.39E+05**	±2.86E+08	±6.02E+08	±7.96E+08	±4.67E+08
F13	3.07E+08	1.01E+08	5.91E+06	4.92E+09	3.49E+10	1.07E+11	**4.24E+03**	1.58E+06	5.32E+04	8.87E+07	9.49E+06
	±3.25E+08	±4.85E+07	±3.67E+06	±1.23E+09	±1.21E+10	±1.89E+10	**±2.49E+03**	±3.40E+06	±2.18E+04	±9.98E+07	±7.63E+06
F14	8.47E+06	9.75E+04	2.22E+06	1.88E+06	1.34E+08	4.42E+08	**1.58E+03**	6.47E+05	2.74E+05	4.29E+05	2.65E+06
	±5.64E+06	±4.83E+04	±1.12E+06	±5.81E+05	±7.08E+07	±2.27E+08	**±3.17E+01**	±2.74E+05	±4.13E+05	±4.31E+05	±1.85E+06
F15	1.89E+06	1.89E+07	1.28E+06	1.47E+09	1.03E+10	3.31E+10	**1.96E+03**	2.25E+05	1.40E+04	5.88E+06	4.85E+05
	±4.94E+06	±6.14E+06	±9.12E+05	±4.69E+08	±4.55E+09	±9.60E+09	**±1.95E+02**	±4.83E+05	±7.76E+03	±7.36E+06	±1.78E+05
F16	4.69E+03	3.75E+03	3.48E+03	5.58E+03	7.68E+03	1.01E+04	3.04E+03	3.62E+03	3.58E+03	**2.90E+03**	5.46E+03
	±3.53E+02	±2.84E+02	±2.20E+02	±3.45E+02	±1.23E+03	±7.17E+02	±3.02E+02	±5.36E+02	±4.57E+02	**±3.53E+02**	±2.08E+02
F17	3.59E+03	3.07E+03	3.32E+03	5.68E+03	2.47E+04	4.58E+04	2.78E+03	2.79E+03	3.46E+03	**2.59E+03**	4.17E+03
	±3.74E+02	±1.92E+02	±2.10E+02	±3.68E+02	±1.58E+04	±2.53E+04	±1.84E+02	±2.01E+02	±3.01E+02	**±2.67E+02**	±2.15E+02
F18	9.46E+06	1.11E+06	5.84E+06	1.78E+07	2.70E+08	1.36E+08	**3.32E+04**	2.74E+06	2.35E+05	2.15E+06	4.16E+07
	±5.06E+06	±6.13E+05	±3.35E+06	±8.84E+06	±1.27E+08	±5.35E+07	**±1.63E+04**	±1.46E+06	±1.04E+05	±1.70E+06	±2.24E+07

F19	3.41E+06	1.07E+07	8.90E+05	8.45E+08	6.18E+09	6.40E+09	**2.15E+03**	8.60E+03	1.89E+04	5.54E+06	2.82E+05
	±6.33E+06	±2.79E+06	±5.24E+05	±2.89E+08	±1.97E+09	±1.46E+09	**±1.87E+02**	±3.12E+03	±1.15E+04	±2.22E+07	±8.04E+04
F20	3.58E+03	3.02E+03	3.20E+03	3.93E+03	4.52E+03	3.98E+03	2.78E+03	2.72E+03	3.45E+03	**2.70E+03**	4.15E+03
	±2.24E+02	±1.73E+02	±1.50E+02	±1.91E+02	±2.47E+02	±7.84E+01	±2.08E+02	±1.43E+02	±3.15E+02	**±2.17E+02**	±1.96E+02
F21	3.00E+03	2.75E+03	2.64E+03	2.88E+03	2.96E+03	3.33E+03	2.54E+03	2.63E+03	2.60E+03	**2.49E+03**	2.78E+03
	±3.52E+01	±3.99E+01	±4.34E+01	±2.91E+01	±4.96E+01	±3.02E+01	±3.77E+01	±3.35E+01	±6.68E+01	**±2.82E+01**	±1.88E+01
F22	1.52E+04	1.23E+04	8.42E+03	1.60E+04	1.46E+04	1.61E+04	9.12E+03	1.42E+04	8.93E+03	**8.09E+03**	1.65E+04
	±2.86E+02	±3.39E+03	±2.41E+03	±4.35E+02	±5.22E+02	±1.93E+02	±3.71E+02	±5.15E+02	±6.97E+02	**±5.14E+02**	±3.53E+02
F23	3.61E+03	3.30E+03	3.13E+03	3.32E+03	4.06E+03	4.72E+03	2.97E+03	3.04E+03	3.32E+03	**2.93E+03**	3.25E+03
	±7.60E+01	±8.55E+01	±4.43E+01	±3.81E+01	±2.33E+02	±1.37E+02	±4.12E+01	±3.70E+01	±1.98E+02	**±3.57E+01**	±2.87E+01
F24	3.74E+03	3.52E+03	3.66E+03	3.42E+03	4.42E+03	5.22E+03	3.17E+03	3.32E+03	3.30E+03	**3.12E+03**	3.40E+03
	±6.38E+01	±1.21E+02	±7.73E+01	±3.27E+01	±2.02E+02	±1.85E+02	±4.68E+01	±3.96E+01	±8.00E+01	**±1.04E+02**	±2.21E+01
F25	4.20E+03	3.14E+03	3.16E+03	7.62E+03	1.19E+04	4.17E+04	2.99E+03	**2.93E+03**	3.03E+03	3.42E+03	4.52E+03
	±3.07E+02	±3.28E+01	±3.94E+01	±1.53E+03	±1.22E+03	±2.90E+03	±3.01E+01	**±2.66E-02**	±3.98E+01	±2.03E+02	±2.86E+02
F26	1.07E+04	6.27E+03	7.55E+03	1.01E+04	1.56E+04	2.60E+04	6.14E+03	6.35E+03	**5.54E+03**	6.05E+03	9.10E+03
	±3.06E+02	±2.59E+03	±7.62E+02	±4.61E+02	±1.17E+03	±1.23E+03	±1.20E+03	±4.14E+02	**±2.84E+03**	±5.63E+02	±2.82E+02
F27	4.18E+03	3.48E+03	3.51E+03	3.72E+03	5.88E+03	7.22E+03	3.26E+03	**3.20E+03**	3.92E+03	3.54E+03	3.92E+03
	±5.24E+01	±1.24E+02	±5.31E+01	±9.70E+01	±4.19E+02	±3.30E+02	±2.86E+01	**±1.43E-04**	±2.15E+02	±7.92E+01	±6.57E+01
F28	4.97E+03	3.43E+03	3.42E+03	6.07E+03	1.02E+04	1.90E+04	**3.29E+03**	3.30E+03	3.34E+03	4.10E+03	5.28E+03
	±3.75E+02	±1.32E+02	±3.99E+01	±1.07E+03	±1.29E+03	±1.03E+03	**±1.61E+01**	±1.47E-04	±1.32E+02	±4.92E+02	±4.80E+02
F29	5.20E+03	5.07E+03	4.28E+03	7.04E+03	5.03E+04	1.10E+05	**4.06E+03**	4.62E+03	4.93E+03	4.32E+03	5.73E+03
	±2.35E+02	±3.18E+02	±1.88E+02	±4.70E+02	±9.37E+04	±6.06E+04	**±1.72E+02**	±4.78E+02	±3.44E+02	±3.01E+02	±3.32E+02
F30	6.89E+06	9.99E+07	2.96E+06	1.05E+09	7.63E+09	9.29E+09	**1.03E+06**	6.57E+06	2.38E+06	7.63E+07	2.48E+07
	±3.00E+06	±1.08E+07	±1.19E+06	±3.34E+08	±2.82E+09	±2.45E+09	**±3.13E+05**	±7.35E+06	±5.06E+05	±3.29E+07	±8.15E+06

Supplementary Material L

Table S31. The running time (seconds) of 11 compared NIOAs when D=10 and D=50

Functions	NIOAs	DE	CS	HS	GSA	GWO	ABC	PSO	FA	GA	BA	IA
F1	D=10	**4.6**	9.5	11.9	110.2	10.8	15.2	9.3	242.2	11.6	17.8	13.0
	D=50	73.2	**55.3**	144.1	960.7	334.1	173.7	179.2	1586.2	86.8	106.7	250.6
F2	D=10	**4.9**	9.8	12.2	109.9	11.0	15.5	9.5	247.0	11.3	18.0	13.4
	D=50	77.7	**61.8**	136.9	937.2	338.8	179.7	195.1	1659.3	94.0	111.7	265.3
F3	D=10	**4.7**	9.7	12.1	109.8	10.9	15.4	9.4	244.8	11.6	17.9	13.0
	D=50	73.0	**55.4**	136.6	914.1	338.6	178.6	188.9	1608.2	97.0	107.6	262.6
F4	D=10	**4.6**	9.6	12.0	109.3	10.7	15.3	9.3	241.4	10.7	17.8	12.9
	D=50	72.7	**55.2**	138.3	915.4	348.1	174.9	189.7	1599.1	89.2	106.3	249.6
F5	D=10	**4.7**	9.7	12.1	109.4	11.0	15.5	9.4	248.1	9.8	18.0	12.9
	D=50	75.1	**57.0**	139.2	917.0	307.0	178.9	191.3	1480.5	84.3	110.1	252.3
F6	D=10	**5.4**	10.3	12.6	110.5	11.4	16.2	9.9	255.5	10.0	18.9	13.4
	D=50	94.1	**74.1**	151.6	936.8	318.1	197.4	201.0	1510.6	93.7	131.2	283.4
F7	D=10	**4.9**	9.7	12.2	110.0	11.1	15.6	9.6	247.0	10.0	18.3	12.8
	D=50	75.7	**57.6**	140.3	910.9	340.0	179.4	191.4	1500.7	85.5	116.2	251.9
F8	D=10	**4.7**	9.6	12.1	111.0	11.0	15.5	9.5	245.6	9.6	18.1	12.8
	D=50	75.8	**57.4**	141.0	912.0	347.6	179.8	190.5	1494.1	84.6	122.6	252.6
F9	D=10	**5.0**	9.8	12.1	109.6	11.1	15.7	9.6	248.6	11.0	18.3	13.1
	D=50	63.2	**47.6**	133.1	908.3	291.3	184.5	136.2	1501.8	65.6	112.0	264.9
F10	D=10	**5.1**	9.9	12.3	7343.3	11.2	15.8	9.7	252.9	10.2	18.5	13.3
	D=50	86.6	**67.1**	147.8	905.3	352.4	188.6	196.2	1505.8	88.9	133.9	265.4
F11	D=10	**4.8**	9.6	12.2	109.5	11.0	15.6	9.6	248.8	10.6	18.2	13.2
	D=50	76.3	**57.8**	143.5	908.5	346.8	179.6	191.2	1500.2	87.1	122.9	265.1
F12	D=10	**4.9**	9.7	12.2	110.6	11.1	15.7	9.6	261.0	11.3	18.4	13.2
	D=50	81.6	**60.8**	144.6	906.4	350.4	182.9	193.6	1502.9	94.6	126.3	258.8
F13	D=10	**4.8**	9.7	12.1	111.1	11.1	15.6	9.6	256.0	10.6	18.2	13.3
	D=50	75.2	**58.0**	143.2	903.7	344.7	178.7	191.5	1500.4	87.2	122.2	255.4
F14	D=10	**5.0**	9.9	12.3	111.4	11.2	15.8	9.8	258.7	10.2	18.5	13.5
	D=50	81.6	**62.8**	143.6	910.3	349.4	186.6	194.3	1504.1	88.9	128.7	273.7
F15	D=10	**4.8**	9.7	12.1	110.5	11.0	15.6	9.6	255.3	10.4	18.2	13.2
	D=50	75.1	**57.6**	142.4	913.7	346.8	178.5	191.3	1496.5	86.3	120.9	252.9
F16	D=10	**5.0**	9.8	12.2	111.4	11.2	15.7	9.7	258.1	10.2	18.5	13.1
	D=50	78.0	**60.2**	144.3	901.5	348.7	182.8	191.9	1621.2	87.6	124.7	256.2
F17	D=10	**5.5**	10.4	12.7	110.8	11.5	16.1	10.0	266.6	10.5	19.1	13.6
	D=50	93.4	**74.4**	156.3	914.4	359.5	183.6	169.0	1696.4	96.6	144.8	287.8
F18	D=10	**4.9**	9.7	12.2	110.3	11.1	15.7	9.6	256.5	10.4	18.3	13.2

	D=50	76.0	**58.6**	145.3	901.8	347.8	181.7	197.9	1523.8	88.9	123.1	263.4
F19	D=10	**7.6**	12.5	13.7	111.4	12.5	17.1	11.0	292.5	11.5	20.9	15.9
	D=50	160.5	**133.8**	192.0	982.3	398.7	249.7	267.5	2773.8	135.4	218.7	383.6
F20	D=10	**5.6**	10.5	12.6	110.8	11.5	16.2	10.1	267.4	10.2	19.0	13.6
	D=50	96.9	**77.7**	158.9	928.6	360.2	201.5	236.0	1926.8	97.2	148.2	287.7
F21	D=10	**5.2**	10.1	12.3	116.1	11.3	15.9	9.8	260.5	9.9	18.4	13.3
	D=50	91.2	70.0	156.5	924.6	355.3	195.1	240.9	1770.6	92.3	140.5	2782.6
F22	D=10	**5.7**	10.5	12.7	110.3	11.6	16.1	10.0	266.6	10.6	19.0	13.7
	D=50	100.5	**78.8**	161.9	931.2	360.1	203.1	239.5	1744.4	97.6	153.3	288.6
F23	D=10	**5.7**	10.6	12.8	110.1	11.6	16.2	102.6	267.1	10.3	19.0	13.8
	D=50	104.7	**76.6**	164.8	930.3	364.2	205.8	239.8	1751.2	99.8	158.5	295.2
F24	D=10	**5.5**	10.3	12.5	110.1	11.5	16.0	9.9	264.0	10.2	18.7	13.5
	D=50	100.2	**67.8**	162.7	926.2	362.7	202.5	236.4	1583.3	98.0	136.5	293.0
F25	D=10	**5.4**	10.3	12.4	110.0	11.4	15.9	9.9	263.6	10.5	18.7	13.3
	D=50	98.0	**65.8**	159.2	915.4	360.5	199.6	234.4	1566.7	99.9	135.2	286.8
F26	D=10	**5.9**	10.8	12.8	110.2	11.7	16.2	10.2	270.9	10.7	19.3	13.8
	D=50	112.7	**78.0**	170.8	930.8	340.0	211.5	243.5	1812.6	104.7	150.3	306.6
F27	D=10	**6.1**	10.9	12.8	110.1	11.7	16.3	10.3	271.9	10.6	19.4	14.0
	D=50	121.3	**83.2**	173.6	940.1	329.9	197.4	247.5	1864.2	108.1	156.2	317.1
F28	D=10	**5.6**	10.5	12.6	110.4	11.5	16.1	10.1	266.0	10.4	18.9	13.5
	D=50	107.4	**74.3**	136.0	928.0	323.3	209.2	240.9	1711.3	103.5	143.3	299.6
F29	D=10	**6.1**	10.9	12.9	111.2	11.9	16.3	10.4	271.4	10.9	19.6	14.2
	D=50	109.6	**75.7**	136.3	933.5	316.8	211.8	236.6	1762.9	105.2	146.9	321.1
F30	D=10	**8.1**	12.9	13.9	111.8	12.8	17.4	11.3	296.5	13.2	21.5	16.3
	D=50	169.8	**130.0**	165.4	992.7	292.5	268.2	236.6	4522.6	140.4	213.0	407.1
